# Calcium orthophosphates (CaPO_4_): occurrence and properties

**DOI:** 10.1007/s40204-015-0045-z

**Published:** 2015-11-19

**Authors:** Sergey V. Dorozhkin

**Affiliations:** Kudrinskaja sq. 1-155, Moscow, 123242 Russia

**Keywords:** Calcium orthophosphates, Hydroxyapatite, Fluorapatite, Bones, Teeth, Antlers, Calcification, Crystallization, Biomimetics

## Abstract

The present overview is intended to point the readers’ attention to the important subject of calcium orthophosphates (CaPO_4_). This type of materials is of the special significance for the human beings because they represent the inorganic part of major normal (bones, teeth and antlers) and pathological (i.e., those appearing due to various diseases) calcified tissues of mammals. For example, atherosclerosis results in blood vessel blockage caused by a solid composite of cholesterol with CaPO_4_, while dental caries and osteoporosis mean a partial decalcification of teeth and bones, respectively, that results in replacement of a less soluble and harder biological apatite by more soluble and softer calcium hydrogenorthophosphates. Therefore, the processes of both normal and pathological calcifications are just an in vivo crystallization of CaPO_4_. Similarly, dental caries and osteoporosis might be considered as in vivo dissolution of CaPO_4_. In addition, natural CaPO_4_ are the major source of phosphorus, which is used to produce agricultural fertilizers, detergents and various phosphorus-containing chemicals. Thus, there is a great significance of CaPO_4_ for the humankind and, in this paper, an overview on the current knowledge on this subject is provided.

## Introduction

Due to the abundance in nature (as phosphate ores) and presence in living organisms (as bones, teeth, deer antlers and the majority of various pathological calcifications), calcium phosphates are the inorganic compounds of a special interest for human being. They were discovered in 1769 and have been investigated since then (Dorozhkin 2012a, 2013a). According to the databases of scientific literature (Web of knowledge, Scopus, Medline, etc.), the total amount of currently available publications on the subject exceeds 40,000 with the annual increase for, at least, 2000 papers. This is a clear confirmation of the importance.

Briefly, by definition, all known calcium phosphates consist of three major chemical elements: calcium (oxidation state +2), phosphorus (oxidation state +5) and oxygen (reduction state −2), as a part of the phosphate anions. These three chemical elements are present in abundance on the surface of our planet: oxygen is the most widespread chemical element of the earth’s surface (~47 mass %), calcium occupies the fifth place (~3.3–3.4 mass %) and phosphorus (~0.08–0.12 mass%) is among the first 20 of the chemical elements most widespread on our planet (Lide 2005). In addition, the chemical composition of many calcium phosphates includes hydrogen, as an acidic orthophosphate anion (for example, HPO_4_^2−^ or H_2_PO_4_^−^), hydroxide [for example, Ca_10_(PO_4_)_6_(OH)_2_] and/or incorporated water (for example, CaHPO_4_·2H_2_O). Regarding their chemical composition, diverse combinations of CaO and P_2_O_5_ oxides (both in the presence of water and without it) provide a large variety of calcium phosphates, which are differentiated by the type of the phosphate anion. Namely, ortho- (PO_4_^3−^), meta- (PO_3_^−^), pyro- (P_2_O_7_^4−^) and poly- ((PO_3_)_n_^n−^) phosphates are known. Furthermore, in the case of multi-charged anions (valid for orthophosphates and pyrophosphates), calcium phosphates are also differentiated by the number of hydrogen ions substituted by calcium ones. The examples comprise mono- (Ca(H_2_PO_4_)_2_), di- (CaHPO_4_), tri- (Ca_3_(PO_4_)_2_) and tetra- (Ca_2_P_2_O_7_) calcium phosphates (LeGeros 1991; Elliott 1994; Amjad 1997). However, to narrow the subject, calcium *ortho*phosphates (abbreviated as CaPO_4_) will be considered and discussed only. Their names, standard abbreviations, chemical formulae and solubility values are listed in Table 1 (Dorozhkin 2011, b). Since all of them belong to CaPO_4_, strictly speaking, all abbreviations in Table 1 are incorrect; however, they have been extensively used in literature for decades and, to avoid confusion, there is no need to modify them.

**Table 1:** Existing calcium orthophosphates and their major properties (Dorozhkin 2011, 2012b)

Ca/P molar ratio	Compound	Formula	Solubility at 25 °C, −log(K_s_)	Solubility at 25 °C (g/L)	pH stability range in aqueous solutions at 25 °C
0.5	Monocalcium phosphate monohydrate (MCPM)	Ca(H_2_PO_4_)_2_·H_2_O	1.14	~18	0.0–2.0
0.5	Monocalcium phosphate anhydrous (MCPA or MCP)	Ca(H_2_PO_4_)_2_	1.14	~17	^c^
1.0	Dicalcium phosphate dihydrate (DCPD), mineral brushite	CaHPO_4_·2H_2_O	6.59	~0.088	2.0–6.0
1.0	Dicalcium phosphate anhydrous (DCPA or DCP), mineral monetite	CaHPO_4_	6.90	~0.048	^c^
1.33	Octacalcium phosphate (OCP)	Ca_8_(HPO_4_)_2_(PO_4_)_4_·5H_2_O	96.6	~0.0081	5.5–7.0
1.5	α-Tricalcium phosphate (α-TCP)	α-Ca_3_(PO_4_)_2_	25.5	~0.0025	^a^
1.5	β-Tricalcium phosphate (β-TCP)	β-Ca_3_(PO_4_)_2_	28.9	~0.0005	^a^
1.2–2.2	Amorphous calcium phosphates (ACP)	Ca_*x*_H_*y*_(PO_4_)_*z*_·*n*H_2_O, *n* = 3–4.5; 15–20 % H_2_O	^b^	^b^	~5–12^d^
1.5–1.67	Calcium-deficient hydroxyapatite (CDHA or Ca-def HA)^e^	C−*x*(HPO_4_)_*x*_(PO_4_)_6−*x*_(OH)_2−*x*_ (0 < *x *< 1)	~85	~0.0094	6.5–9.5
1.67	Hydroxyapatite (HA, HAp or OHAp)	Ca_10_(PO_4_)_6_(OH)_2_	116.8	~0.0003	9.5–12
1.67	Fluorapatite (FA or FAp)	Ca_10_(PO_4_)_6_F_2_	120.0	~0.0002	7–12
1.67	Oxyapatite (OA, OAp or OXA)^f^, mineral voelckerite	Ca_10_(PO_4_)_6_O	~69	~0.087	^a^
2.0	Tetracalcium phosphate (TTCP or TetCP), mineral hilgenstockite	Ca_4_(PO_4_)_2_O	38–44	~0.0007	^a^

^a^These compounds cannot be precipitated from aqueous solutions

^b^Cannot be measured precisely. However, the following values were found: 25.7 ± 0.1 (pH = 7.40), 29.9 ± 0.1 (pH = 6.00), 32.7 ± 0.1 (pH = 5.28) (Ohura et al. 1996). The comparative extent of dissolution in acidic buffer is: ACP ≫ α-TCP ≫ β-TCP > CDHA ≫ HA > FA (Daculsi et al. 1997)

^c^Stable at temperatures above 100 °C

^d^Always metastable

^e^Occasionally, it is called “precipitated HA (PHA)”

^f^Existence of OA remains questionable

In general, the atomic arrangement of all CaPO_4_ is built up around a network of orthophosphate (PO_4_) groups, which stabilize the entire structure. Therefore, the majority of CaPO_4_ are sparingly soluble in water (Table 1); however, all of them are easily soluble in acids but insoluble in alkaline solutions. In addition, all chemically pure CaPO_4_ are colorless transparent crystals of moderate hardness but, as powders, they are of white color. Nevertheless, natural minerals of CaPO_4_ are always colored due the presence of impurities and dopants, such as ions of Fe, Mn and rare earth elements (Cantelar et al. 2001; Ribeiro et al. 2005). Biologically formed CaPO_4_ are the major component of all mammalian calcified tissues (Lowenstam and Weiner 1989), while the geologically formed ones are the major raw material to produce phosphorus-containing agricultural fertilizers, chemicals and detergents (McConnell 1973; Becker 1989; Rakovan and Pasteris 2015).

## Geological and biological occurrences

**Fig. 1:**
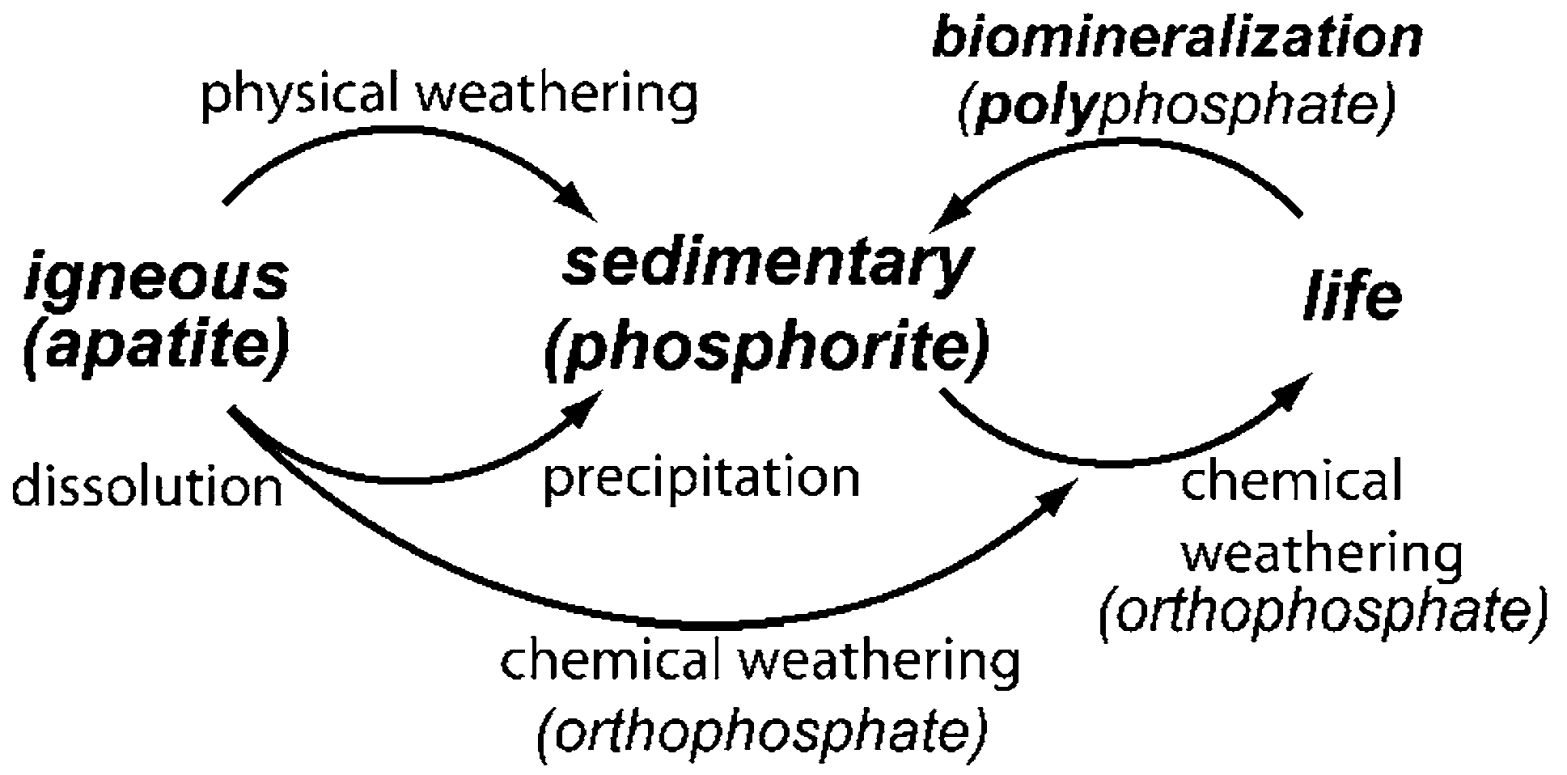
A simplified schematic of the phosphorus cycle from apatitic igneous rock to phosphorite sedimentary rock through chemical or physical weathering. Life forms accumulate soluble phosphorus species and can produce apatite through biomineralization. Reprinted from Ref. (Omelon and Grynpas 2008) with permission

Geologically, natural CaPO_4_ are found in different regions mostly as deposits of apatites, mainly as ion-substituted FA (igneous rocks), and phosphorites (sedimentary rocks) (Becker 1989; Rakovan and Pasteris 2015; Cook et al. 2005; Dumoulin et al. 2011). In addition, natural ion-substituted CDHA was also found (Mitchell et al. 1943) but it is a very rare mineral. Some types of sedimentary rocks can be formed by weathering of igneous rocks into smaller particles (Zhang et al. 2010a). Other types of sedimentary rocks can be composed of minerals precipitated from the dissolution products of igneous rocks or minerals produced by biomineralization (Fig. 1; Omelon and Grynpas 2008). Thus, due to a sedimentary origin, both a general appearance and a chemical composition of natural phosphorites vary a lot (Jarvis 1994; Glenn 1994). It is a common practice to consider francolite (or carbonate-hydroxyfluorapatite regarded as its synonym) as the basic phosphorite mineral (Cook et al. 2005; McClellan 1980; Mcarthur 1985; Zanin 2004; Lapin and Lyagushkin 2014). According to Henry (1850), the name francolite was given by Mr. Brooke and Mr. Nuttall to a mineral from Wheal Franco, Tavistock, Devon, some years prior to 1850. A cryptocrystalline (almost amorphous) variety of francolite (partly of a biological origin) is called collophane (synonyms: collophanit, collophanita, collophanite, grodnolite, kollophan), named in 1870 by Karl Ludwig Fridolin von Sandberger from the Greek roots κολλα (=glue) and φαινεσθαι (to appear) referring to the appearance of the mineral (Rogers 1922; Cao et al. 2013; http://www.mindat.org/min-10072.html). Francolite is found in natural phosphorites predominantly as fossil bones and phosphatized microbial pseudomorphs: phosphatic crusts of chasmolithic biofilms (or microstromatolites) and globular clusters with intra-particular porosities (Elorza et al. 1999; Hubert et al. 2005; Xiao et al. 1998, 2000). Natural phosphorites (therefore, francolite and collophane as well) occur in various forms, such as nodules, crystals or masses. Occasionally, other types of natural CaPO_4_ are found as minerals, for example clinohydroxylapatite (Chakhmouradian and Medici 2006), staffelite (synonyms: staffelit, staffelita) belonging to carbonate-rich fluorapatites (chemical formula: Ca_5_[(F,O)(PO_4_,CO_3_)_3_]) (Mason et al. 2009; http://www.mindat.org/gallery.php?min=9293) and DCPD (Klein 1901; Kaflak-Hachulska et al. 2000). Furthermore, CaPO_4_ were found in meteoric stones (Merrill 1917; McCubbin and Nekvasil 2008; McCubbin et al. 2014). The world deposits of natural CaPO_4_ are estimated to exceed 150 billion tons; from which approximately 85 % belong to phosphorites and the remaining ~15 % belong to apatites (Cook et al. 2005).

**Fig. 2:**
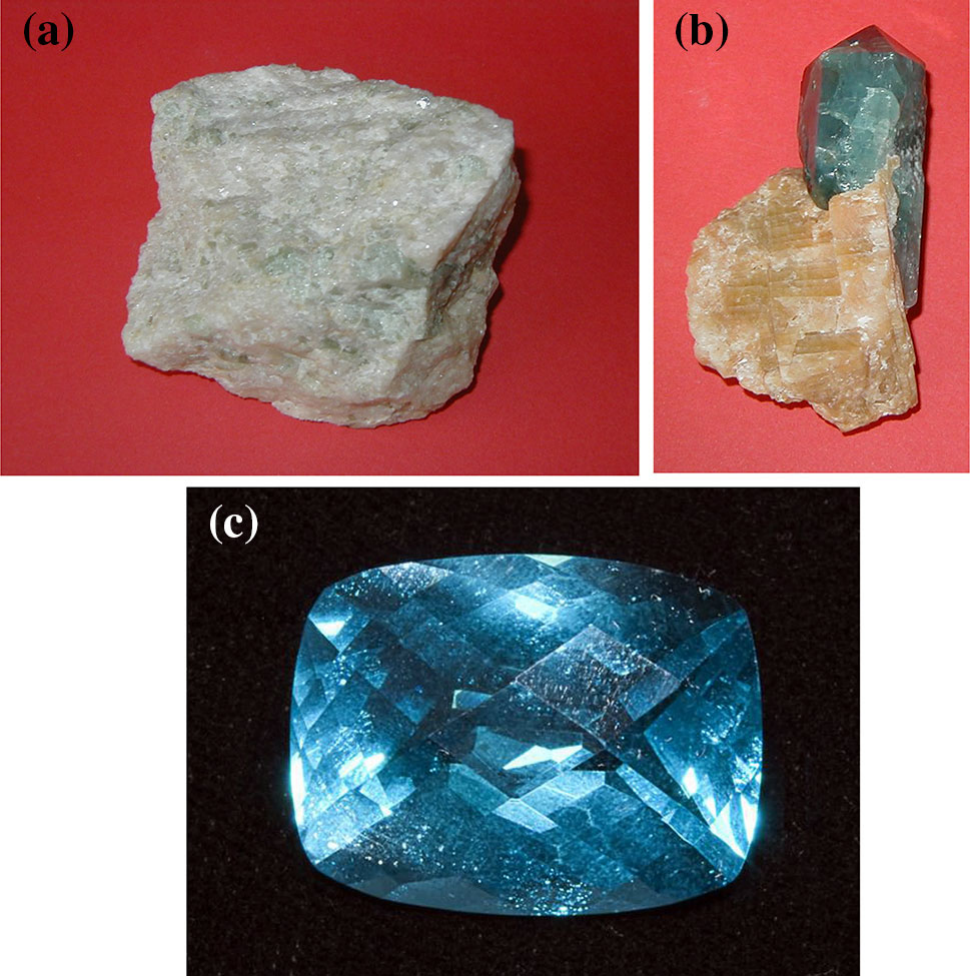
Samples of natural FA: (**a**) polycrystalline, (**b**) single-crystalline and (**c**) a gem. The *colors* are due to incorporated ions of transition metals

As minor constituents (<~5 %), natural CaPO_4_ (both apatites and phosphorites) occur in many geological environments. Concentrations sufficient for economic use (>15 %) are also available. Namely, the largest world deposits of natural apatites are located in Russia [the Khibiny and Kovdor massifs, Kola peninsula (Lapin and Lyagushkin 2014; Tyrrell 1938; Kogarko 1999)], Brazil and Zambia, while the largest world deposits of natural phosphorites are located in Morocco, Russia, Kazakhstan, USA (Florida, Tennessee), China and Australia (McConnell 1973; Becker 1989; Rakovan and Pasteris 2015; Cook et al. 2005). In addition, they are found at seabed and ocean floor (Baturin 2012). There is an opinion, that the marine phosphorites could be formed due to microbial activity (Schulz and Schulz 2005; Crosby and Bailey 2012). The majority of natural CaPO_4_ occur as small polycrystalline structures (spherulitic clusters). Larger crystals are rare (Ford 1917). They usually have the crystal structure of apatites (hexagonal system, space group *P*6_3_/m). Giant crystals including “a solid but irregular mass of green crystalline apatite, 15 feet long and 9 feet wide” (Hogarth 1974) and a single euhedral crystal from the Aetna mine measuring 2.1 × 1.2 m with an estimated weight of 6 tons (van Velthuizen 1992) were found. None of them is a pure compound; they always contain dopants of other elements. For example, ions of orthophosphate may be partly replaced by AsO_4_^3−^, CO_3_^2−^ and VO_4_^3−^ (Trueman 1966), ions of calcium might be partially replaced by Sr, Ba, Mg, Mn, K, Na, Fe, while ions of hydroxide, chloride, bromide, carbonate and oxide may to a certain extent substitute fluoride in the crystal lattice of natural apatites (Pan and Fleet 2002). Furthermore, organic compounds have been found in natural apatites (Gilinskaya 2010; Gilinskaya and Zanin 2012). In principle, the crystal structure of apatites can incorporate half the Periodic Chart of the elements in almost any valence state into its atomic arrangement. Namely, substituents such as the first row transition elements and the lanthanides (they act as activators and chromophores) impart colors and can lead to luminescence. Furthermore, crystal imperfections, such as site vacancies, vacancies with trapped electrons and point defect clusters, can likewise influence both color and luminescence (Rakovan and Pasteris 2015). The substitutions in apatites are usually in trace concentrations; however, for certain dopants (e.g., F^−^ and OH^−^) large concentrations and even complete solid solutions exist. To make things even more complicated, some ions in the crystal structure may be missing, leaving the crystallographic defects, which leads to formation of non-stoichiometric compounds, such as CDHA. Due to their affinity for chromophoric substituents and propensity for other defects, natural apatites are found in just about all colors of the rainbow. Ease of atomic substitution for apatite leaves this mineral open to a wide array of compositions. This might be related to the fact that the apatite structure type displays porous properties (White et al. 2005). In medicine, this property might be used as an antidote for heavy metal intoxication (Sánchez-Salcedo et al. 2010). Figure 2 shows some examples of natural FA.

Manufacturing of elementary phosphorus (white and red) (Jacob and Reynolds 1928; Emsley 2001), phosphoric acids (Becker 1989; Dorozhkin 1996, 1997, 1998; Gilmour 2014), various P-containing chemicals, agricultural fertilizers [namely, superphosphate (Copson et al. 1936; Newton and Copson 1936; Rossete et al. 2008), ammonium orthophosphates (Magda et al. 2008)] and detergents [principally sodium tripolyphosphate (Kijkowska et al. 2007)] are the major industrial applications of natural CaPO_4_. The annual consumption of a phosphate rock has approached ~150 million tons and about 95 % of this production is utilized in the fertilizer industry (Abouzeid 2007, 2008). However, the significance of CaPO_4_ to the society is by no means limited to their role as a source of phosphorus; all currently available applications have been summarized in Table 2 (Rakovan and Pasteris 2015).

**Table 2:** The principal technological and scientific uses of apatites and other calcium orthophosphates (Rakovan and Pasteris 2015)

Application	Properties utilized
Geology	
Petrogenetic indicator	Major- and trace-element composition
Geochronology (dating)	Radionuclide composition, fission tracks
Ore of phosphorus and rare earth elements	Composition (P and rare earth elements)
Environmental	
Heavy metal and phosphate sequestration	Elemental affinity, chemical stability, insolubility
Solid nuclear waste form	Thermal and chemical stability, annealing temperature, elemental affinity
Water treatment	Elemental affinity, deflocculant
Fertilizer	Constituent phosphate
Biology/medicine	
Orthopedics	Natural constituent of bones
Dentistry	Natural constituent of teeth
Nanoparticle drug delivery agent	Size, morphology, structure, solubility, biocompatibility
Prosthetic coating, bone and tooth replacement media	Compositional and structural similarity to mineral in bones and teeth
Materials	
Phosphors	Optical emission
Lasers	Optical emission and lasing behavior
Gems	Color, diaphaneity, chatoyancy

In biological systems, many organisms, ranging from bacteria and isolated cells to invertebrates and vertebrates, synthesize CaPO_4_ (Omelon and Grynpas 2008). Formation of solid CaPO_4_ in primitive organisms is believed to enable the storage and regulation of essential elements such as calcium, phosphorus and, possibly, magnesium. The morphology of precipitates in these organisms (small intracellular nodules of ACP often located in mitochondria) complies with the necessities for rapid mobilization and intracellular control of the concentration of these elements (Rey et al. 2006). In vertebrates CaPO_4_ occur as the principal inorganic constituent of normal (bones, teeth, fish enameloid, deer antlers and some species of shells) and pathological (dental and urinary calculus and stones, atherosclerotic lesions, etc.) calcifications (Lowenstam and Weiner 1989; O’Neill 2007; LeGeros 2001; Wopenka and Pasteris 2005; Pasteris et al. 2008; Sun and Hanley 2007). In addition, they are found in ganoid fish scales (in alligator gar and Senegal bichir), turtle shells, as well as in armadillo and alligator osteoderms (Currey 2010). In minute quantities CaPO_4_ exist in the brain (brain sand), without significantly affecting its function (Bocchi and Valdre 1993). Therefore, the expression “having sand in the head” is not without a reason. Except for small portions of the inner ear, all hard tissues of the human body are formed of CaPO_4_. Structurally, they occur mainly in the form of poorly crystalline, non-stoichiometric, Na-, K-, Mg- and carbonate-containing CDHA (Young 1975; Danilchenko 2013). It is often called “biological apatite” (Young 1975; Danilchenko 2013; Nakano et al. 2002a; Grynpas and Omelon 2007; Bazin et al. 2014a) [which might be abbreviated as BAp (Lee et al. 2009; Basaruddin and Takano 2014)], bioapatite (Eagle et al. 2010; Cherkinsky et al. 2013; Šupová 2014) or dahllite (Lowenstam and Weiner 1985; Fernandez et al. 1998). The latter was named in 1888 by Brögger and Bäckström (1890) after the Swedish mineralogist brothers Tellef and Johan Martin Dahll.

The main constituents of human bones are CaPO_4_ (~60–70 wt%), collagen (~20–30 wt%) and water (up to 10 wt%) (Bocchi and Valdre 1993; Skinner 2005; Daculsi et al. 1997). An interesting cautionary tale on the knowledge development about the structurally incorporated water in bone apatite is available in literature (Pasteris 2012). The detailed information on the chemical composition of the most important human normal calcified tissues is comprised in Table 3. One should note that the values mentioned in Table 3 are approximate; the main constituents can vary by a percent or more (Driessens and Verbeeck 1990). Due to the aforementioned effect of lattice flexibility, bones act as both the mineral reservoir of the body and the storage for toxic elements, thus fulfilling two of its essential physiological roles.

**Table 3:** Comparative composition and structural parameters of inorganic phases of adult human calcified tissues

	Enamel	Dentine	Cementum	Bone	HA
Composition (wt%)					
Calcium^a^	36.5	35.1	~35	34.8	39.6
Phosphorus (as P)^a^	17.7	16.9	~16	15.2	18.5
Ca/P (molar ratio)^a^	1.63	1.61	~1.65	1.71	1.67
Sodium^a^	0.5	0.6	^c^	0.9	–
Magnesium^a^	0.44	1.23	0.5–0.9	0.72	–
Potassium^a^	0.08	0.05	^c^	0.03	–
Carbonate (as CO_3_^2−^)^b^	3.5	5.6	^c^	7.4	–
Fluoride^a^	0.01	0.06	Up to 0.9	0.03	–
Chloride^a^	0.30	0.01	^c^	0.13	–
Pyrophosphate (as P_2_O_7_^4−^)^b^	0.022	0.10	^c^	0.07	–
Total inorganic^b^	97	70	60	65	100
Total organic^b^	1.5	20	25	25	–
Water^b^	1.5	10	15	10	–
Crystallographic properties: lattice parameters (±0.003 Å)					
*a*-axis (Å)	9.441	9.421	^c^	9.41	9.430
*c*-axis (Å)	6.880	6.887	^c^	6.89	6.891
Crystallinity index (HA = 100)	70–75	33–37	~30	33–37	100
Typical crystal sizes (nm) (Lowenstam and Weiner 1989; Weiner and Wagner 1998)	100 µm × 50 × 50	35 × 25 × 4	^c^	50 × 25 × 4	200–600
Ignition products (800 °C)	β-TCP + HA	β-TCP + HA	β-TCP + HA	β-TCP + HA	HA
Elastic modulus (GPa)	80	23.8 ± 3.7	15.0 ± 3.6	0.34–13.8	10
Tensile strength (MPa)	10	100	^c^	150	100

Due to the considerable variation found in biological samples, typical values are given in these cases (LeGeros 1991; Daculsi et al. 1997)

^a^Ashed samples

^b^Unashed samples

^c^Numerical values were not found in the literature but they should be similar to those for dentine

Finally, one should mention, that, in a dissolved state, CaPO_4_ are found in many biological liquids, such as blood serum (Floege et al. 2011), urine (Suller et al. 2005), sweat (Prompt et al. 1978), and milk (Holt 1982) (Lenton et al. 2015) and, therefore, in dairy products (Gaucheron 2012).

## The members of CaPO_4_ family

**Fig. 3:**
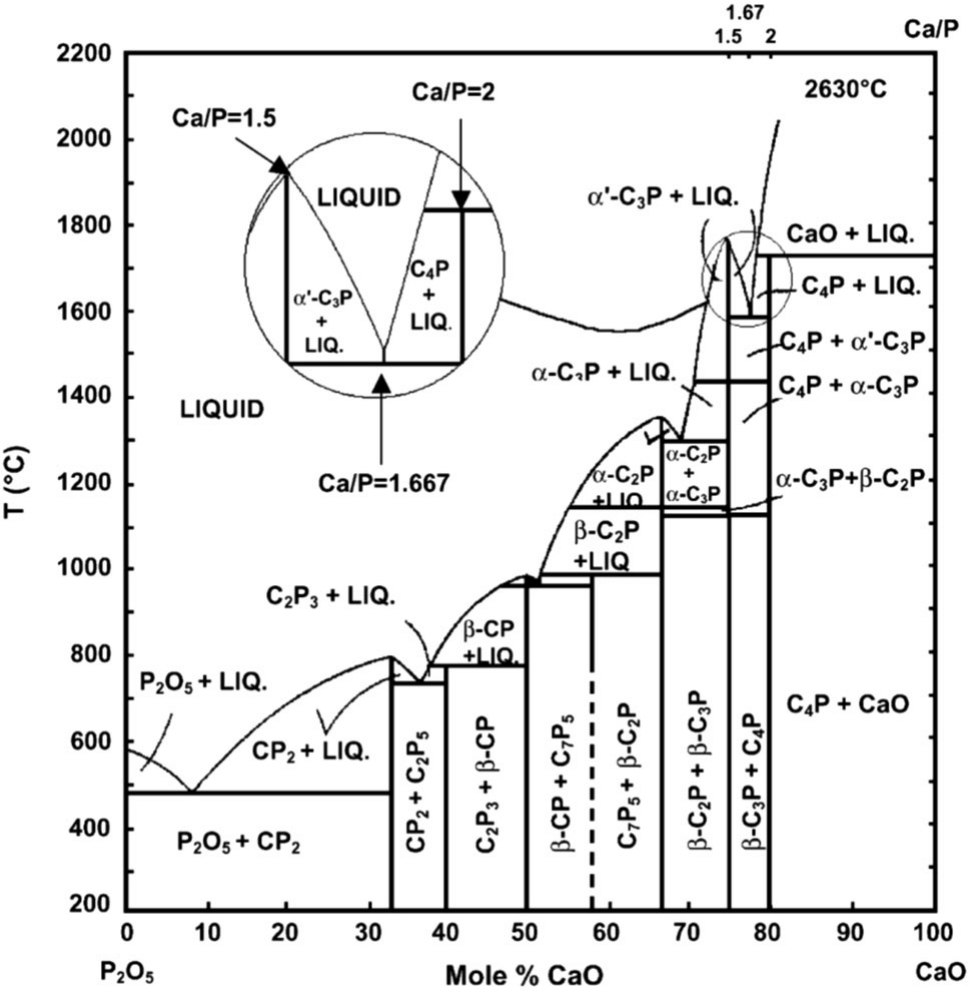
Phase diagram of the system CaO–P_2_O_5_ (C=CaO, P=P_2_O_5_) at elevated temperatures. Here: C_7_P_5_ means 7CaO·5P_2_O_5_; other abbreviations should be written out in the same manner. Reprinted from Refs. (Kreidler and Hummel 1967; Carayon and Lacout 2003) with permission

In the ternary aqueous system Ca(OH)_2_–H_3_PO_4_–H_2_O (or CaO–P_2_O_5_–H_2_O) (Clark 1931; Brown 1992; Martin and Brown 1997), there are twelve known non-ion-substituted CaPO_4_ with the Ca/P molar ratio ranging between 0.5 and 2.0 (Table 1). An anhydrous phase diagram CaO–P_2_O_5_ at temperatures within 200–2200 °C is shown in Fig. 3 (Kreidler and Hummel 1967; Carayon and Lacout 2003). Table 4 comprises crystallographic data of the existing CaPO_4_ (Elliott 1994; White and Dong 2003; Mathew and Takagi 2001). The most important parameters of CaPO_4_ are the ionic Ca/P ratio, basicity/acidity and solubility. All these parameters strongly correlate with the solution pH. The lower the Ca/P molar ratio is, the more acidic and water-soluble the CaPO_4_ is (LeGeros 1991; Elliott 1994; Amjad 1997). Therefore, the Ca/P ratio can be used as a fingerprint of the CaPO_4_ phases. One can see that the solubility ranges from high values for acidic compounds, such as MCPM, to very low values for basic compounds, such as apatites, which allowing CaPO_4_ to be dissolved, transported from one place to another and precipitated, when necessary. Crystallization, dissolution and phase transformation processes of different CaPO_4_ under various experimental conditions have been reviewed (Wang and Nancollas 2008). Regarding applications, some of them might be used in food industry and, according to the European classification of food additives, CaPO_4_ of food grade quality are known as E341 additive.

**Table 4:** Crystallographic data of calcium orthophosphates (Elliott 1994; White and Dong 2003; Mathew and Takagi 2001)

Compound	Space group	Unit cell parameters	*Z* ^a^	Density (g cm^−3^)
MCPM	Triclinic *P* $\bar{1}$	*a* = 5.6261 (5), *b* = 11.889 (2), *c* = 6.4731 (8) Å, *α* = 98.633 (6)º, *β* = 118.262 (6)º, *γ* = 83.344 (6)º	2	2.23
MCPA	Triclinic *P* $\bar{1}$	*a* = 7.5577 (5), *b* = 8.2531 (6), *c* = 5.5504 (3) Å, *α* = 109.87 (1)º, *β* = 93.68 (1)º, *γ* = 109.15 (1)º	2	2.58
DCPD	Monoclinic *I*a	*a* = 5.812 (2), *b* = 15.180(3), *c* = 6.239 (2) Å, *β* = 116.42 (3)º	4	2.32
DCPA	Triclinic *P* $\bar{1}$	*a* = 6.910 (1), *b* = 6.627 (2), *c* = 6.998 (2) Å, *α* = 96.34 (2)º, *β* = 103.82 (2)º, *γ* = 88.33 (2)º	4	2.89
OCP	Triclinic *P* $\bar{1}$	*a* = 19.692 (4), *b* = 9.523 (2), *c* = 6.835 (2) Å, *α* = 90.15 (2)º, *β* = 92.54 (2)º, *γ* = 108.65 (1)º	1	2.61
α-TCP	Monoclinic *P*2_1_/a	*a* = 12.887 (2), *b* = 27.280 (4), *c* = 15.219 (2) Å, *β* = 126.20 (1)º	24	2.86
β-TCP	Rhombohedral *R*3cH	*a* = *b* = 10.4183 (5), *c* = 37.3464 (23) Å, *γ* = 120°	21^b^	3.08
HA	Monoclinic *P*2_1_/b or hexagonal *P*6_3_/m	*a* = 9.84214 (8), *b* = 2*a*, *c* = 6.8814 (7) Å, *γ* = 120° (monoclinic) *a* = *b* = 9.4302 (5), *c* = 6.8911 (2) Å, *γ* = 120º (hexagonal)	4 2	3.16
FA	Hexagonal *P*6_3_/m	*a* = *b* = 9.367, *c* = 6.884 Å, *γ* = 120º	2	3.20
OA	Hexagonal *P* $\overline{6}$	*a* = *b* = 9.432, *c* = 6.881 Å, *α* = 90.3°, *β* = 90.0°, *γ* = 119.9°	1	~3.2
TTCP	Monoclinic *P*2_1_	*a* = 7.023 (1), *b* = 11.986 (4), *c* = 9.473 (2) Å, *β* = 90.90 (1)º	4	3.05

^a^Number of formula units per unit cell

^b^Per the hexagonal unit cell

**Fig. 4:**
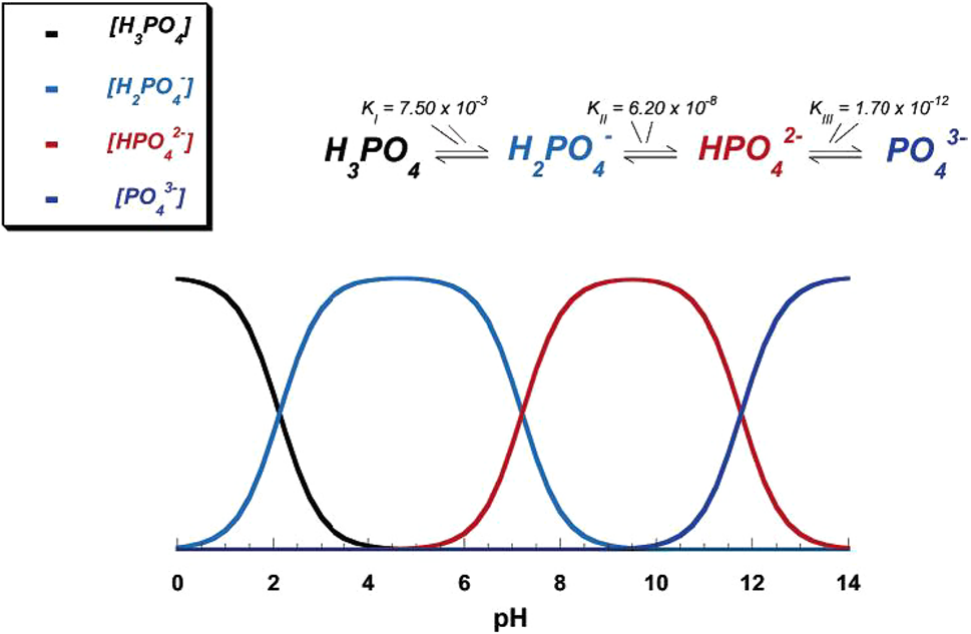
pH variation of ionic concentrations in triprotic equilibrium for orthophosphoric acid solutions. Reprinted from Ref. (Lynn and Bonfield 2005) with permission

**Fig. 5:**
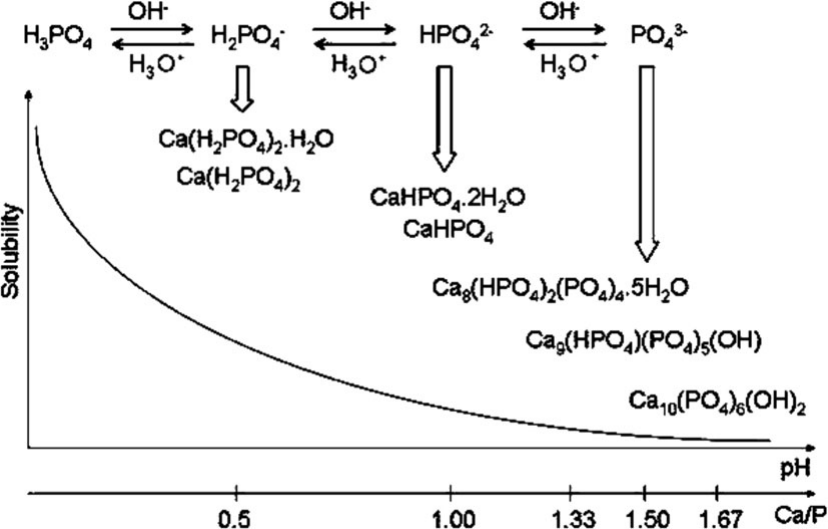
Various types of CaPO_4_ obtained by neutralizing of orthophosphoric acid by calcium hydroxide. The Ca/P values of the known types of CaPO_4_ (Table 1) are reported in the figure. The solubility of CaPO_4_ in water decreases drastically from *left* to *right*, HA being the most insoluble and stable phase. Reprinted from Ref. (León and Jansen 2009) with permission

**Fig. 6:**
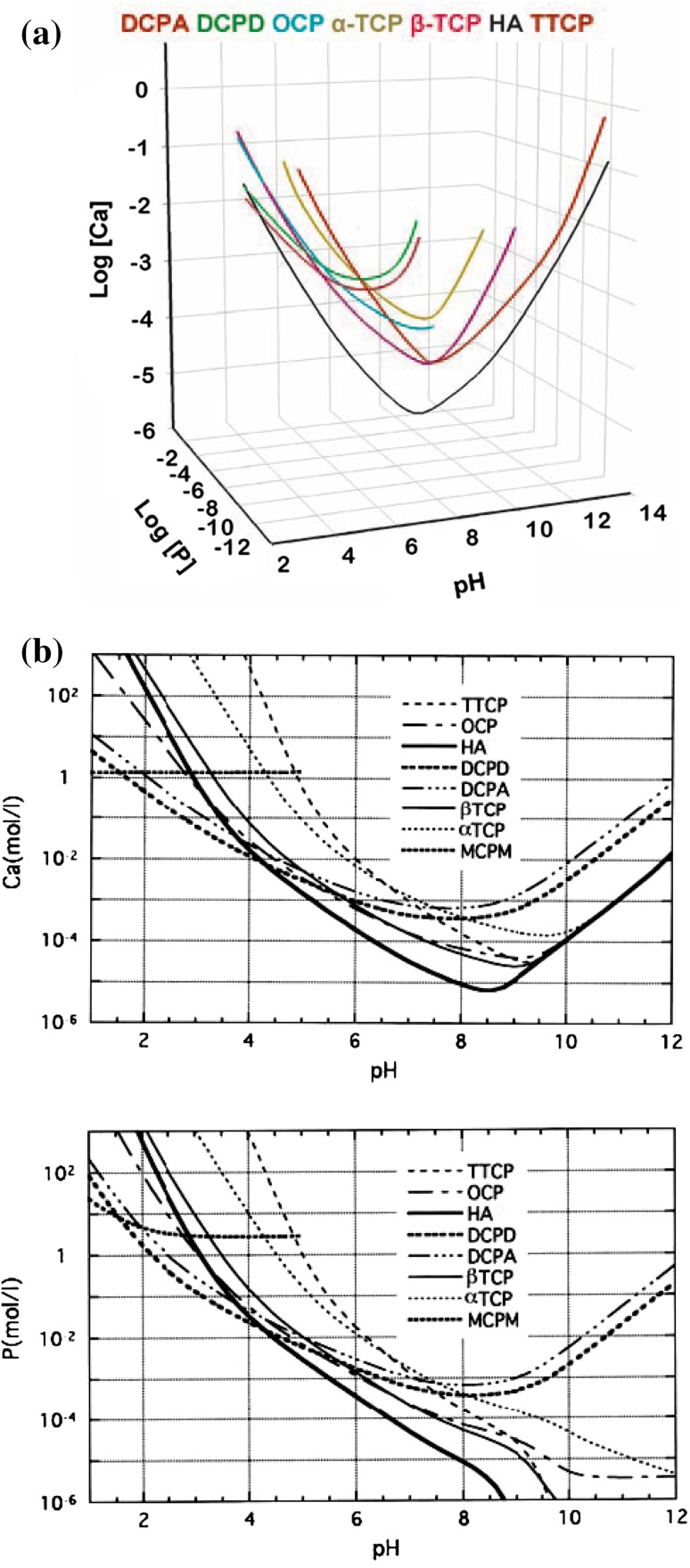
*Top*: a 3D version of the classical solubility phase diagrams for the ternary system Ca(OH)_2_–H_3_PO_4_–H_2_O. Reprinted from Ref. (Chow 2009) with permission. *Middle* and *bottom*: solubility phase diagrams in two-dimensional graphs, showing two logarithms of the concentrations of (**a**) calcium and (**b**) orthophosphate ions as a function of the pH in solutions saturated with various salts. Reprinted from Ref. (Ishikawa 2010) with permission

Due to the triprotic equilibrium that exists within orthophosphate-containing solutions, variations in pH alter the relative concentrations of the four types of anionic species of orthophosphoric acid (Fig. 4; Lynn and Bonfield 2005) and thus both the chemical composition (Fig. 5; León and Jansen 2009) and the amount of the CaPO_4_ that are formed by a direct precipitation. The solubility isotherms of different CaPO_4_ are shown in Fig. 6 (Elliott 1994; Amjad 1997; Brown 1992; Martin and Brown 1997; McDowell et al. 1977; Chow 2009; Ishikawa 2010). However, in 2009, the classic solubility data of CaPO_4_ (Elliott 1994; Amjad 1997; Brown 1992; Martin and Brown 1997; McDowell et al. 1977; Chow 2009; Ishikawa 2010) were mentioned to be inappropriate (Pan and Darvell 2009a). According to the authors of the latter study, all previous solubility calculations were based on simplifications, which were only crudely approximate. The problem lies in incongruent dissolution, leading to phase transformations and lack of the detailed solution equilibria. Using an absolute solid-titration approach, the true solubility isotherm of HA was found to lie substantially lower than previously reported. In addition, contrary to a wide belief, DCPD appeared not to be the most stable phase below pH ~4.2, where CDHA was less soluble (Pan and Darvell 2009a).

A brief description of all known CaPO_4_ (Table 1) is given below.

### MCPM

Monocalcium phosphate monohydrate [Ca(H_2_PO_4_)_2_·H_2_O; the IUPAC name is calcium dihydrogen orthophosphate monohydrate] is both the most acidic and water-soluble CaPO_4_. Although acidic CaPO_4_ in general were known by 1795 as “super-phosphate of lime” (Fourcroy 1804), their differentiation started in 1800s. Namely, by 1807, researchers first prepared a calcium phosphate, which could be attributed to MCPM (Aikin and Aikin 1807).

MCPM crystallizes from aqueous solutions containing dissolved ions of H_2_PO_4_^−^ and Ca^2+^ at the Ca/P ratio ~0.5 and solution pH below ~2.0. Besides, MCPM might be precipitated from aqueous solutions containing organic solvents (Boonchom 2009; Kongteweelert et al. 2013). At temperatures above ~100 °C, MCPM releases a molecule of water and transforms into MCPA but at temperatures >~500 °C MCPA further transforms into Ca(PO_3_)_2_ (Tynsuaadu 1990). The results of the spectroscopic investigations of MCPM are available in literature (Xu et al. 1998).

Due to high acidity and solubility, MCPM is never found in biological calcifications. Moreover, pure MCPM is not biocompatible with bones (Köster et al. 1977). However, in medicine MCPM is used as a component of several self-hardening CaPO_4_ formulations (Huan and Chang 2009; Dorozhkin 2013b). In addition, MCPM is used as a nutrient, acidulant and mineral supplement for food, feed and some beverages (Budavari et al. 1996; Stein et al. 2008). Coupled with NaHCO_3_, MCPM is used as a leavening agent for both dry baking powders and bakery dough. MCPM might be added to salt-curing preserves, pickled and marinated foods. In addition, MCPM might be added to tooth pastes and chewing gums (Dorozhkin 2013c). Besides, MCPM might be added to ceramics and glasses, while agriculture is the main consumer of a technical grade MCPM, where it is used as a fertilizer, triple superphosphate (Nasri et al. 2015).

### MCPA (or MCP)

Monocalcium phosphate anhydrous [Ca(H_2_PO_4_)_2_; the IUPAC name is calcium dihydrogen orthophosphate anhydrous] is the anhydrous form of MCPM. Although MCPM has been known since 1807 (Dorozhkin 2012a, 2013a), MCPA was differentiated as “tetra-hydrogen calcium phosphate, H_4_Ca(PO_4_)_2_” by 1879 (Roscoe and Schorlemmer 1879). It crystallizes under the same conditions as MCPM but at temperatures above ~100 °C (e.g., from concentrated hot mother liquors during fertilizer production). In addition, MCPA might be prepared from MCPM by dehydration. Furthermore, it might be also prepared at ambient temperatures by crystallization in water-restricted or non-aqueous systems. Like MCPM, MCPA never appears in calcified tissues and is not biocompatible due to its acidity. There is no current application of MCPA in medicine. Due to the similarity with MCPM, in many cases, MCPA might be used instead of MCPM; however, highly hydroscopic properties of MCPA reduce its commercial applications (Becker 1989; Budavari et al. 1996).

### DCPD

Dicalcium phosphate dihydrate [CaHPO_4_·2H_2_O; the IUPAC name is calcium hydrogen orthophosphate dihydrate; the mineral brushite] has been known since, at least, 1804 (Fourcroy 1804). As a mineral, brushite was first discovered in phosphatic guano from Avis Island (Caribbean) in 1865 (Moore 1865) and named to honor an American mineralogist Prof. George Jarvis Brush (1831–1912), Yale University, New Haven, Connecticut, USA.

DCPD can be easily crystallized from aqueous solutions containing dissolved ions of HPO_4_^2−^ and Ca^2+^ at the Ca/P ratio ~1 and solution pH within ~2.0 < pH < ~6.5 (Hamai et al. 2013). Other preparation techniques such as neutralization of H_3_PO_4_ and/or MCPM solutions by CaO, CaCO_3_ or more basic CaPO_4_ (α- or β-TCP, CDHA, HA, TTCP) are also known. Interestingly, that precipitation of DCPD by mixing a Ca(OH)_2_ suspension and a H_3_PO_4_ solution in the equimolar quantities was found to occur in five stages, being HA the first precipitated phase (Ferreira et al. 2003; Oliveira et al. 2007). Besides, DCPD might be prepared in gels (Sivkumar et al. 1997; Madhurambal et al. 2009). DCPD transforms into DCPA at temperatures above ~80 °C and this transformation is accompanied by ~11 % decrease in volume (MacDowell et al. 1971) and structural changes (Landin et al. 1994). The value for Δ_*r*_ *G*° for DCPD → DCPA transformation is −1.032 kJ/mol (Landin et al. 1994). Briefly, DCPD crystals consist of CaPO_4_ chains arranged parallel to each other, while lattice water molecules are interlayered between them (Curry and Jones 1971). Liquid ordering at the {010} DCPD/water interface was determined (Arsic et al. 2004). In 2009, data on DCPD solubility were updated by solid titration technique (Pan and Darvell 2009b). The optical properties of DCPD were described (Lundager-Madsen 2008), while many additional data on DCPD including a good drawing of its atomic structure might be found in Ref. (Qiu and Orme 2008).

DCPD is of biological importance because it is often found in pathological calcifications (dental calculi, crystalluria, chondrocalcinosis and urinary stones) and some carious lesions (LeGeros 1991, 2001; O’Neill 2007). It was proposed as an intermediate in both bone mineralization and dissolution of enamel in acids (dental erosion) (LeGeros 1991, 2001; O’Neill 2007). In medicine, DCPD is used in self-setting CaPO_4_ formulations (Dorozhkin 2013b) and as an intermediate for tooth remineralization. DCPD is added to toothpaste both for caries protection (in this case, it is often coupled with F-containing compounds such as NaF and/or Na_2_PO_3_F) and as a gentle polishing agent (Dorozhkin 2013c). Other applications include a flame retardant (Mostashari et al. 2006), a slow release fertilizer, using in glass production, as well as calcium supplement in food, feed and cereals. In food industry, it serves as a texturizer, bakery improver and water retention additive. In diary industry, DCPD is used as a mineral supplement. In addition, plate-like crystals of DCPD might be used as a non-toxic, anticorrosive and passivating pigment for some ground coat paints (Budavari et al. 1996).

### DCPA (or DCP)

Dicalcium phosphate anhydrous (CaHPO_4_; the IUPAC name is calcium hydrogen orthophosphate anhydrate; the mineral monetite) is the anhydrous form of DCPD. Although DCPD has been known since, at least, 1804 (Dorozhkin 2012a, 2013a), DCPA was differentiated as “mono-hydrogen CaPO_4_, HCaPO_4_” by 1879 (Roscoe and Schorlemmer 1879). As a mineral, monetite was first described in 1882 in rock-phosphate deposits from the Moneta (now Monito) Island (archipelago of Puerto Rico), which contains a notable occurrence (Shepard 1882).

Due to the absence of water inclusions, DCPA is less soluble than DCPD (Table 1). Like DCPD, DCPA can be crystallized from aqueous solutions containing Ca/P ratio ~1 at solution pH within ~2.0 < pH < ~6.5 but at temperatures >~90 °C. In addition, DCPA might be prepared by dehydration of DCPD. Furthermore, it might be also prepared at ambient temperatures in water-restricted or non-aqueous systems, such as gels (Sivkumar et al. 1997), ethanol (Tas 2009), as well as in the oil-in-water and water-in-oil systems (Chen et al. 2005). DCPA is physically stable and resisted hydration even when dispersed in water for over 7 months in the temperature range of 4–50 °C (Miyazaki et al. 2009). A calcium-deficient DCPA was also prepared. It might be sintered at ~300 °C (Eshtiagh-Hosseini et al. 2008). Unlike DCPD, DCPA occurs in neither normal nor pathological calcifications. It is used in self-setting CaPO_4_ formulations (Dorozhkin 2013b). Besides, DCPA might be implanted as bioceramics (Tamimi et al. 2010). Other applications include using as a polishing agent, a source of calcium and phosphate in nutritional supplements (e.g., in prepared breakfast cereals, enriched flour and noodle products), a tableting aid (Takami et al. 1996) and a toothpaste component (Dorozhkin 2013c). In addition, it is used as a dough conditioner in food industry (Budavari et al. 1996). DCPA of a technical grade of purity might be used as a fertilizer (Habashi 2011).

### OCP

Octacalcium phosphate [Ca_8_(HPO_4_)_2_(PO_4_)_4_·5H_2_O; the IUPAC name is tetracalcium hydrogen orthophosphate diorthophosphate pentahydrate, another name is octacalcium bis(hydrogenphosphate) tetrakis(phosphate) pentahydrate] is often found as an unstable transient intermediate during the precipitation of the thermodynamically more stable CaPO_4_ (e.g., CDHA) in aqueous solutions. To the best of my findings (Dorozhkin 2012a, 2013a), OCP has been known since, at least, 1843, when Percy published a paper (Percy 1843), in which he described formation of “a new hydrated phosphate of lime” with a chemical formula 2CaO + PO5 + 6HO, in which “1 equiv. water being basic and 5 constitutional”. However, according to Bjerrum (Bjerrum 1958), a CaPO_4_ with the OCP composition was first described by Berzelius in 1836.

The preparation techniques of OCP are available in literature (LeGeros 1985; Nakahira et al. 2001; Arellano-Jiménez et al. 2009; Suzuki 2010a). Briefly, to prepare OCP, Ca- and PO_4_-containing chemicals must be mixed to get the supersaturated aqueous solutions with the Ca/P ratio equal to 1.33. Typically, OCP crystals are smaller if compared to DCPD ones, extremely platy and almost invariably twinned. However, OCP might be non-stoichiometric and be either Ca-deficient (down to Ca/P = 1.26) or include excessive calcium (up to Ca/P = 1.48) in the structure (Suzuki 2010a). It has been proposed that the structure of a non-stoichiometric OCP contains an excess of hydrogen, resulting in a non-stoichiometric chemical formula Ca_16_H_4+*x*_(PO_4_)_12_(OH)_*x*_·(10 − *x*)H_2_O, which resembles the structure of HA even more closely than previously anticipated (Mathew et al. 1988). Furthermore, a partially hydrolyzed form of OCP with Ca/P molar ratio of 1.37 might be prepared (Suzuki 2010a; Miyatake et al. 2009). At solution pH = 7.2 and temperature 60 °C, the full hydrolysis of OCP into CDHA occurs within ~6 h (Arellano-Jiménez et al. 2009). Ion-substituted OCP might be prepared as well (Matsunaga 2008a; Boanini et al. 2010a).

The triclinic structure of OCP displays apatitic layers (with atomic arrangements of calcium and orthophosphate ions similar to those of HA) separated by hydrated layers (with atomic arrangements of calcium and orthophosphate ions similar to those in DCPD) (LeGeros 1991; Elliott 1994; Amjad 1997; Suzuki 2010a). A similarity in crystal structure between OCP and HA (Brown 1962; Brown et al. 1962) is one reason that the epitaxial growth of these phases is observed. It is generally assumed that, in solutions, the hydrated layer of the (100) face is the layer most likely exposed to solution. The water content of OCP crystals is ~20 % that of DCPD and this is partly responsible for its lower solubility. The latest data on OCP structure (Davies et al. 2012) and solubility (Pan and Darvell 2009c) are available.

OCP is of a great biological importance because it is one of the stable components of human dental and urinary calculi (Chow and Eanes 2001; Kakei et al. 2009). OCP was first proposed by W. E. Brown to participate as the initial phase in enamel mineral formation and bone formation through subsequent precipitation and stepwise hydrolysis of OCP (Brown 1962, 1966; Brown et al. 1962). It plays an important role in formation of apatitic biominerals in vivo (Suzuki 2010b). A “central OCP inclusion” (also known as “central dark line”) is seen by transmission electron microscopy in many biological apatites and in synthetically precipitated CDHA (Iijima et al. 1996; Bodier-Houllé et al. 1998; Rodríguez-Hernández et al. 2005). Although OCP has not been observed in vascular calcifications, it has been strongly suggested as a precursor phase to biological apatite found in natural and prosthetic heart valves (Tomazic et al. 1994; Nancollas and Wu 2000). In surgery, OCP is used for implantation into bone defects (Suzuki et al. 2006; Kikawa et al. 2009; Murakami et al. 2010; Suzuki 2013). For the comprehensive information on OCP, the readers are referred to other reviews (Suzuki 2010a; Chow and Eanes 2001; Suzuki 2013).

### β-TCP

β-tricalcium phosphate [β-Ca_3_(PO_4_)_2_; the IUPAC name is tricalcium diorthophosphate beta, other names are CaPO_4_ tribasic beta or tricalcium bis(orthophosphate) beta] is one of the polymorphs of TCP. Although CaPO_4_ with the composition close to that of TCP, CDHA and HA were known in 1770s (Dorozhkin 2012a, 2013a), α- and β-polymorphs of TCP were differentiated only by 1932 (Bredig et al. 1932; Trömel 1932).

β-TCP cannot be precipitated from aqueous solutions. It is a high-temperature phase, which can be prepared at temperatures above ~800 °C by thermal decomposition of CDHA or by solid-state interaction of acidic CaPO_4_, e.g., DCPA, with a base, e.g., CaO. In all cases, the chemicals must be mixed in the proportions to get the Ca/P ratio equal to 1.50. However, β-TCP can also be prepared at relatively low temperatures (~150 °C) by precipitation in water-free mediums, such as ethylene glycol (Tao et al. 2008, 2009). Apart from the chemical preparation routes, ion-substituted β-TCP can be prepared by calcining of bones (Hou et al. 2008; Santos et al. 2012): such type of CaPO_4_ is occasionally called “bone ash” (Lee et al. 2013). At temperatures above ~1125 °C, β-TCP is transformed into a high-temperature phase α-TCP. Being the stable phase at room temperature, β-TCP is less soluble in water than α-TCP (Table 1). Both ion-substituted (Ito and LeGeros 2008; Kannan et al. 2009, 2010; Quillard et al. 2012) and organically modified (Karlinsey and Mackey 2009; Karlinsey et al. 2010a, b) forms of β-TCP can be synthesized, as well. The modern structural data on β-TCP are available in Refs. (Yashima et al. 2003; Yin et al. 2003; Liang et al. 2010; Zhai and Wu 2010), Raman spectra of β-TCP might be found if Ref. (Zhai et al. 2015), while solubility data—in Ref. (Pan and Darvell 2009d). Furthermore, an ability of β-TCP to store an electrical charge by electrical polarization was studied and this material was found to have a suitable composition and structure for both ion conduction and charge storage (Wang et al. 2010).

Pure β-TCP never occurs in biological calcifications. Only a Mg-substituted form [β-TCMP—β-tricalcium magnesium phosphate, β-(Ca,Mg)_3_(PO_4_)_2_, which is often called whitlockite to honor Mr. Herbert Percy Whitlock (1868–1948), an American mineralogist, the curator of the American Museum of Natural History, New York City, New York, USA (Frondel 1941)] is found. Since β-TCMP is less soluble than β-TCP (Li et al. 2009a), it is formed instead of β-TCP in dental calculi and urinary stones, dentineal caries, salivary stones, arthritic cartilage, as well as in some soft-tissue deposits (LeGeros 1991, 2001; O’Neill 2007; Kodaka et al. 1988; Reid and Andersen 1993; Scotchford and Ali 1995; P’ng et al. 2008). However, it has not been observed in enamel, dentine or bone. In medicine, β-TCP is used in the self-setting CaPO_4_ formulations (Dorozhkin 2013b) and other types of bone grafts (Hou et al. 2008; Horch et al. 2006; Ogose et al. 2006; Kamitakahara et al. 2008; Liu et al. 2008; Epstein 2009; Liu and Lun 2012). Dental applications of β-TCP are also known. For example, β-TCP is added to some brands of toothpaste as a gentle polishing agent (Dorozhkin 2013c). Multivitamin complexes with CaPO_4_ are widely available in the market and β-TCP is used as the calcium phosphate there. In addition, β-TCP serves as a texturizer, bakery improver and anti-clumping agent for dry powdered food (flour, milk powder, dried cream, cocoa powder). Besides, β-TCP is added as a dietary or mineral supplement to food and feed (Güngörmüş et al. 2010). Occasionally, β-TCP might be used as inert filler in pelleted drugs. Other applications comprise porcelains, pottery, enamel, using as a component for mordants and ackey, as well as a polymer stabilizer (Budavari et al. 1996). β-TCP of a technical grade (as either calcined natural phosphorites or bone dust) is used as a slow release fertilizer for acidic soils (Becker 1989).

To conclude, one should briefly mention on an existence of γ-TCP polymorph, naturally known as tuite, which was named after Prof. Guangzhi Tu (born in 1920), a founding director of the Guangzhou Institute of Geochemistry, Chinese Academy of Sciences, Guangzhou, China. Tuite appears to be a high-pressure polymorph of whitlockite with an empirical formula ${\text{Ca}}_{ 2. 5 1} {\text{Na}}_{0. 2 8} {\text{Mg}}_{0. 2 7} {\text{Fe}}_{0.02}^{2 + } \left( {{\text{PO}}_{ 4} } \right)_{ 2.0 2}$. Pure γ-TCP polymorph can be synthesized from β-TCP at pressures above ~4 GPa and temperatures above ~1000 °C (Murayama et al. 1986; Xie et al. 2003; Zhai et al. 2009, 2014).

### α-TCP

α-Tricalcium phosphate [α-Ca_3_(PO_4_)_2_; the IUPAC name is tricalcium diorthophosphate alpha, other names are CaPO_4_ tribasic alpha or tricalcium bis(orthophosphate) alpha] is another polymorph of TCP, which was differentiated by 1932 (Bredig et al. 1932; Trömel 1932). α-TCP is also a high-temperature phase; therefore, it cannot be precipitated from aqueous solutions either. Thus, α-TCP is usually prepared by the same techniques as β-TCP (see the previous section) but, since the β-TCP → α-TCP transition temperature is ~1125 °C (Welch and Gutt 1961), calcining is performed at temperatures above ~1200 °C (Jokic et al. 2007). Consequently, α-TCP is often considered as a high-temperature polymorph of β-TCP. However, data are available that α-TCP might be prepared at lower temperatures. Namely, at the turn of the millennium, the previously forgotten data that the presence of silicates stabilized α-TCP at temperatures of 800–1000 °C (Nurse et al. 1959) were rediscovered again. Such type of α-TCP is called “silica stabilized α-TCP” (Sayer et al. 2003; Reid et al. 2005, 2006). Furthermore, sometimes, α-TCP might be prepared at even lower temperatures (~700 °C) by a thermal decomposition of low-temperature ACPs (Kanazawa et al. 1982).

Although α-TCP and β-TCP have exactly the same chemical composition, they differ by the crystal structure (Table 4) and solubility (Table 1). In the absence of humidity, both polymorphs of TCP are stable at room temperatures; however, according to a density functional study, stability of β-TCP crystal lattice exceeds that of α-TCP (Yin et al. 2003). Therefore, of them, α-TCP is more reactive in aqueous systems, has a higher specific energy and in aqueous solutions it can be hydrolyzed to CDHA (TenHuisen and Brown 1998; Durucan and Brown 2000, 2002). Milling was found to increase the α-TCP reactivity even more (Camiré et al. 2005). Although, α-TCP never occurs in biological calcifications, in medicine, it is used as a component of self-setting CaPO_4_ formulations (Budavari et al. 1996). On the other hand, the chemically pure α-TCP has received not much interest in the biomedical field (Kamitakahara et al. 2008). The disadvantage for using α-TCP is its quick resorption rate (faster than formation of a new bone), which limits its application in this area. However, the silicon stabilized α-TCP (more precisely as a biphasic composite with HA) has been commercialized as a starting material to produce bioresorbable porous ceramic scaffolds to be used as artificial bone grafts (Sayer et al. 2003; Reid et al. 2005, 2006). Upon implantation, α-TCP tends to convert to CDHA, which drastically reduces further degradation rate. Theoretical insights into bone grafting properties of the silicon-stabilized α-TCP might be found in Ref. (Yin and Stott 2005). The structure of α-TCP is well described in literature (Yin et al. 2003; Liang et al. 2010), while the surface and adsorption properties are available in Ref. (Yin and Stott 2006). Similar to β-TCP, α-TCP of a technical grade might be used slow release fertilizer for acidic soils (Budavari et al. 1996).

To conclude, one should briefly mention on an existence of α′-TCP polymorph, which was discovered in 1959 (Nurse et al. 1959). However, this TCP polymorph lacks of any practical interest because it only exists at temperatures between ~1450 °C and its melting point (~1756 °C). It reverts to α-TCP polymorph by cooling below the transition temperature. Additional details on α-TCP are available in the topical review (Carrodeguas and de Aza 2011).

### ACP

Amorphous calcium phosphates (ACPs) represent a special class of CaPO_4_ salts, having variable chemical but rather identical glass-like physical properties, in which there are neither translational nor orientational long-range orders of the atomic positions. To the best of my findings (Dorozhkin 2012a, 2013a), ACP was first prepared in 1845 (Jones 1845). Nevertheless, until recently (Dorozhkin 2012c), ACP has often been considered as an individual CaPO_4_ compound with a variable chemical composition, while, in reality, ACP is just an amorphous state of other CaPO_4_. Therefore, in principle, all compounds mentioned in Table 1 might be somehow fabricated in an amorphous state but, currently, only few of them (e.g., an amorphous TCP) are known (Dorozhkin 2012c). Thus, strictly speaking, ACP should be excluded from Table 1. Furthermore, since ACPs do not have the definite chemical composition, the IUPAC nomenclature is not applicable to describe them.

Depending on the production temperatures, all types of ACP are divided into two major groups: low-temperature ACPs (prepared in solutions, usually aqueous ones) and high-temperature ACPs (Dorozhkin 2012c). Low-temperature ACPs (described by the chemical formula Ca_*x*_H_*y*_(PO_4_)_*z*_·*n*H_2_O, *n* = 3–4.5; 15–20 % H_2_O) are often encountered as a transient precursor phase during precipitation of other CaPO_4_ in aqueous systems. Usually, an ACP is the first phase precipitated from supersaturated solutions (the higher supersaturation, the better) prepared by rapid mixing of solutions containing ions of calcium and orthophosphate (Elliott 1994; Dorozhkin 2012c). Such ACP precipitates usually look like spherical particles with diameters in the range 200–1200 Å without a definite structure. Generally, the ACP particles are smaller if prepared under conditions of high supersaturation and/or high pH, while for a given pH, higher temperatures give larger particles (Blumenthal et al. 1972). The freshly precipitated ACPs contain 10–20 % by weight of tightly bound water, which is removed by vacuum drying at elevated temperature (Posner and Betts 1975). The amorphization degree of ACPs increases with the concentration increasing of Ca- and PO_4_-containing solutions, as well as at a high solution pH and a low crystallization temperature. A continuous gentle agitation of as precipitated ACPs in the mother solution, especially at elevated temperatures, results in a slow recrystallization and formation of better crystalline CaPO_4_, such as CDHA (LeGeros 1991; Elliott 1994). In addition, other production techniques of ACPs are known (Dorozhkin 2012c).

The lifetime of ACPs in aqueous solutions was reported to be a function of the presence of additive molecules and ions, pH, ionic strength and temperature. In addition, confinement was found to increase their lifetime (Wang et al. 2014a). Thus, ACPs may persist for appreciable periods and retain the amporphous state under some specific experimental conditions (Termine et al. 1970). The chemical composition of ACPs strongly depends on the solution pH and the concentrations of mixing solutions. For example, ACPs with Ca/P ratios in the range of 1.18 (precipitated at solution pH = 6.6) to 1.53 (precipitated at solution pH = 11.7) (Elliott 1994, 1998) and even to 2.5 (LeGeros 1991, 2001; O’Neill 2007) were described. In deed and not in name, these data mean that various types of CaPO_4_ were prepared in an amorphous state (Dorozhkin 2012c). It should be noted that unsubstituted ACPs are unstable in aqueous solutions and even when stored dry they tend to transform into more crystalline CaPO_4_, such as poorly crystalline CDHA. The presence of poly(ethylene glycol) (Li and Weng 2007), ions of pyrophosphate, carbonate and/or magnesium in solutions during the crystallization promotes formation of ACPs and slows down their further transformation, while the presence of fluoride has the opposite effect (LeGeros 1991; Elliott 1994; Amjad 1997; Daculsi et al. 1997; Tadic et al. 2002). In general, low-temperatures ACPs heated to ~550 °C (so that all volatiles have already escaped) remain amorphous, but further heating above ~650 °C causes their transformation into crystalline CaPO_4_, such as α- or β-TCP, HA, mixtures thereof, depending on the Ca/P ratio of the ACP heated.

High-temperature ACPs might be prepared using high energy processing at elevated temperatures (Dorozhkin 2012c). This method is based on a rapid quenching of melted CaPO_4_ occurring, e.g., during plasma spraying of HA (Carayon and Lacout 2003; Keller and Dollase 2000; Kumar et al. 2004). A plasma jet, possessing very high temperatures (~5000 to ~20,000 °C), partly decomposes HA, which results in formation of a complicated mixture of products, some of which would be ACPs. Obviously, all types of high-temperature ACPs are definitively anhydrous contrary to the precipitated ACPs. Unfortunately, no adequate chemical formula is available to describe the high-temperature ACPs.

**Fig. 7:**
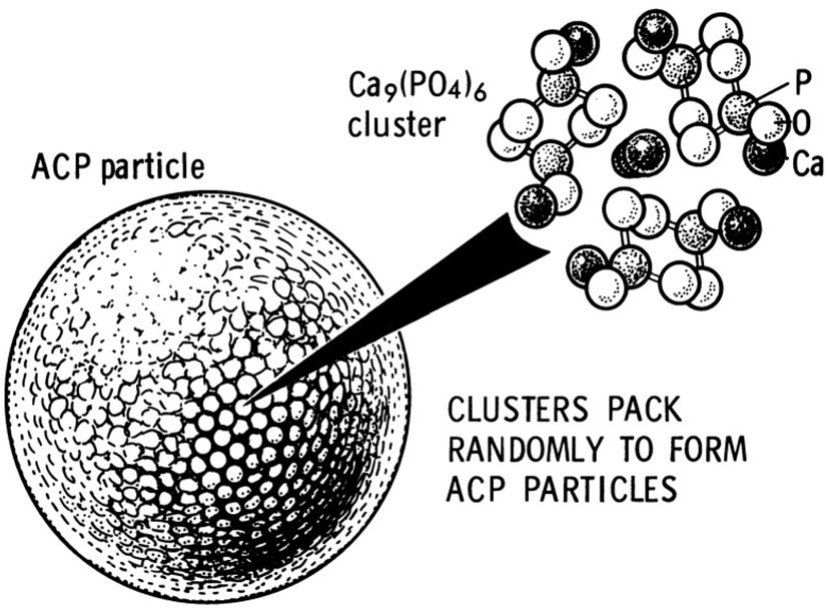
A model of ACP structure. Reprinted from Ref. (Posner et al. 1980) with permission

In general, as all amorphous compounds are characterized by a lack of long-range order, it is problematic to discuss the structure of ACPs (they are X-ray amorphous). Concerning a short-range order (SRO) in ACPs, it exists, just due to the nature of chemical bonds. Unfortunately, in many cases, the SRO in ACPs is uncertain either, because it depends on many variables, such as Ca/P ratio, preparation conditions, storage and admixtures. Infrared spectra of ACPs show broad featureless phosphate absorption bands. Electron microscopy of freshly precipitated ACPs usually shows featureless nearly spherical particles with diameters in the range of 20–200 nm. However, there is a questionable opinion that ACPs might have an apatitic structure but with a crystal size so small, that they are X-ray amorphous. This is supported by X-ray absorption spectroscopic data (EXAFS) on biogenic and synthetic samples (Harries et al. 1986, 1987; Taylor et al. 1998; Peters et al. 2000). On the other hand, it was proposed that the basic structural unit of the precipitated ACPs is a 9.5 Å diameter, roughly spherical cluster of ions with the composition of Ca_9_(PO_4_)_6_ (Fig. 7; Elliott 1994, 1998; Posner et al. 1980; Boskey 1997). These clusters were found experimentally as first nuclei during the crystallization of CDHA and a model was developed to describe the crystallization of HA as a step-wise assembly of these units (Onuma and Ito 1998) [see “HA (or HAp, or OHAp)”]. Biologically, ion-substituted ACPs (always containing ions of Na, Mg, carbonate and pyrophosphate) are found in soft-tissue pathological calcifications (e.g., heart valve calcifications of uremic patients) (LeGeros 1991, 2001; O’Neill 2007).

In medicine, ACPs are used in self-setting CaPO_4_ formulations (Dorozhkin 2013b). Bioactive composites of ACPs with polymers have properties suitable for use in dentistry (Dorozhkin 2012c, 2013c) and surgery (Dorozhkin 2012c; Tadic and Epple 2002). Due to a reasonable solubility and physiological pH of aqueous solutions, ACPs appeared to be consumable by some microorganisms and, due to this reason, it might be added as a mineral supplement to culture media. Non-biomedical applications of ACPs comprise their using as a component for mordants and ackey. In food industry, ACPs are used for syrup clearing. Occasionally, they might be used as inert filler in pelleted drugs. In addition, ACPs are used in glass and pottery production and as a raw material for production of some organic phosphates. To get further details on ACPs, the readers are referred to special reviews (Dorozhkin 2012c; Boskey 1997; Combes and Rey 2010).

### CDHA (or Ca-def HA, or CDHAp)

Calcium-deficient hydroxyapatite [Ca_10-*x*_(HPO_4_)_*x*_(PO_4_)_6-*x*_(OH)_2-*x*_ (0 < *x* < 1)] became known since the earliest experiments on establishing the chemical composition of bones performed in 1770s (Dorozhkin 2012a, 2013a). However, the first appropriate term “subphosphate of lime” appeared by 1819 (Bache 1819). Other chemical formulae such as Ca_10-*x*_(HPO_4_)_2*x*_(PO_4_)_6−2*x*_(OH)_2_ (0 < *x* < 2), Ca_10−*x*−*y*_(HPO_4_)_*x*_(PO_4_)_6-*x*_(OH)_2−*x*−2*y*_ (0 < *x* < 2 and *y* < *x*/2), Ca_10−*x*_(HPO_4_)_*x*_(PO_4_)_6−*x*_(OH)_2−*x*_(H_2_O)_*x*_ (0 < *x* < 1), and Ca_9−*x*_(HPO_4_)_1+2*x*_(PO_4_)_5−2*x*_(OH) were also proposed to describe its variable composition (Elliott 1994). As seen from these formulae, Ca deficiency is always coupled with both OH deficiency and protonation of some PO_4_ groups with simultaneous formation of the ionic vacancies in the crystal structure (Wilson et al. 2005). In addition, CDHA often contains tightly bound water molecules, which might occupy some of these ionic vacancies. For example, there is an approach describing a lack of the hydroxide vacancies in CDHA: to perform the necessary charge compensation of the missing Ca^2+^ ions, a portion of OH^−^ anions is substituted by neutral water molecules (Zahn and Hochrein 2008). This water is removed by vacuum drying at elevated temperature. Concerning possible vacancies of orthophosphate ions, nothing is known about their presence in CDHA. It is just considered that a portion of PO_4_^3−^ ions is either protonated (as HPO_4_^2−^) or substituted by other ions (e.g., CO_3_^2−^) (Ivanova and Frank-Kamenetskaya 2001). Since CDHA does not have any definite chemical composition, the IUPAC nomenclature is not applicable to describe it.

CDHA can be easily prepared by simultaneous addition of Ca- and PO_4_-containing solutions in the proportions to get Ca/P ratio within 1.50–1.67 into boiling water followed by boiling the suspension for several hours (an aging stage). That is why, in literature, it might be called as “precipitated HA (PHA)” (Sinha et al. 2003; Mayer et al. 2003). Besides, it might be prepared by hydrolysis of α-TCP (TenHuisen and Brown 1998; Durucan and Brown 2000, 2002). Other preparation techniques of CDHA are known as well (Vallet-Regí et al. 1997; Siddharthan et al. 2004; Hutchens et al. 2006; Mochales et al. 2011). During aging, initially precipitated ACPs are restructured and transformed into CDHA. Therefore, there are many similarities in the structure, properties and application between the precipitated in alkaline solutions (pH > 8) ACPs and CDHA. Some data indicated on a presence of intermediate phases during further hydrolysis of CDHA to a more stable HA-like phase (Brès et al. 2005). In general, CDHA crystals are poorly crystalline and of submicron dimensions. They have a very large specific surface area, typically 25–100 m^2^/g. On heating above ~700 °C, CDHA with Ca/P = 1.5 converts to β-TCP and that with 1.5 < Ca/P < 1.67 converts into a biphasic composite of HA and β-TCP (see “Biphasic, triphasic and multiphasic CaPO_4_ formulations”) (Dorozhkin 2012d). A solid-state transformation mechanism of CDHA into HA + β-TCP biocomposite was proposed (Dorozhkina and Dorozhkin 2002a; Dorozhkin 2003).

The variability in Ca/P molar ratio of CDHA has been explained through different models: surface adsorption, lattice substitution and intercrystalline mixtures of HA and OCP (Rodríguez-Lorenzo 2005). Due to a lack of stoichiometry, CDHA is usually doped by other ions (Rey et al. 2006). The doping extent depends on the counter-ions of the chemicals used for CDHA preparation. Direct determinations of the CDHA structures are still missing and the unit cell parameters remain uncertain. However, unlike that in ACPs (see “ACP”), a long-range order exists in CDHA. Namely, the following lattice parameters were reported for CDHA with Ca/P = 1.5: *a* = 9.4418 (20) Å and *c* = 6.8745 (17) Å (Mochales et al. 2011).

Systematic studies of defect constellations in CDHA are available in literature (Zahn and Hochrein 2008; Liou et al. 2004). As a first approximation, CDHA may be considered as HA with some ions missing (ionic vacancies) (Brown and Martin 1999). The more amount of Ca is deficient, the more disorder, imperfections and vacancies are in the CDHA structure (Honghui et al. 2007). Furthermore, a direct correlation between the Ca deficiency and the mechanical properties of the crystals was found: calcium deficiency lead to an 80 % reduction in the hardness and elastic modulus and at least a 75 % reduction in toughness in plate-shaped HA crystals (Viswanath et al. 2010). More recently, using first-principles calculations, a reduction in the elastic constants and moduli of CDHA due to vacancies was reported (Sun et al. 2013; Bhat et al. 2014). Theoretical investigations of the defect formation mechanism relevant to non-stoichiometry in CDHA are available elsewhere (Matsunaga 2008b).

Undoped CDHA (i.e., that containing ions of Ca^2+^, PO_4_^3−^, HPO_4_^2−^ and OH^−^ only) does not exist in biological systems. However, the ion-substituted CDHA: Na^+^, K^+^, Mg^2+^, Sr^2+^ for Ca^2+^; CO_3_^2−^ for PO_4_^3−^ or HPO_4_^2−^; F^−^, Cl^−^, CO_3_^2−^ for OH^−^, plus some water forms biological apatite—the main inorganic part of animal and human normal and pathological calcifications (LeGeros 1991; Rey et al. 2006; O’Neill 2007). Therefore, CDHA is a very promising compound for industrial manufacturing of artificial bone substitutes (Bourgeois et al. 2003), including drug delivery applications (Liu et al. 2005). Non-biomedical applications of CDHA are similar to those of ACP and HA. Interesting that CDHA was found to possess a catalytic activity to produce biogasoline (Tsuchida et al. 2008).

### HA (or HAp, or OHAp)

Hydroxyapatite [Ca_5_(PO_4_)_3_(OH), but is usually written as Ca_10_(PO_4_)_6_(OH)_2_ to denote that the crystal unit cell comprises two molecules; the IUPAC name is pentacalcium hydroxide tris(orthophosphate)] is the second most stable and least soluble CaPO_4_ after FA. Apatites were recognized as calcium phosphates by, at least, 1789 (Dorozhkin 2012a, 2013a). Here, it is worth noting that *hydroxylapatite* would be a more accurate abbreviation expansion of HA (perhaps, *hydroxideapatite* would be even better because it relates to calcium hydroxide) while by both the medical and material communities HA is usually expanded as *hydroxyapatite*.

Chemically pure HA crystallizes in the monoclinic space group *P*2_1_/b (Elliott et al. 1973). However, at temperatures above ~250 °C, there is a monoclinic to hexagonal phase transition in HA (space group *P*6_3_/m) (Elliott 1994, 1998; Mathew and Takagi 2001; Rangavittal et al. 2000; Kim et al. 2000a). The structural difference between the two modifications represents the ordered, head-to-tail arrangement of OH groups located in the center of every other Ca_2_ triangle in the monoclinic low-temperature symmetry and the disordered arrangement of OH groups, where the head-to-tail and tail-to-head arrangements alternate throughout the channel in the hexagonal high-temperature symmetry. This induces strains that might be compensated by substitutions and/or ion vacancies. Some impurities, like partial substitution of hydroxide by fluoride or chloride, stabilize the hexagonal structure of HA at ambient temperature. Due to this reason, hexagonal HA is seldom the stoichiometric phase and very rare single crystals of natural HA always exhibit the hexagonal space group. The detailed description of the HA structure was first reported in 1964 (Kay et al. 1964) and its interpretation in terms of aggregation of Ca_9_(PO_4_)_6_ clusters, the so-called Posner’s clusters, has been widely used since publication of the article by Posner and Betts (Posner and Betts 1975).

Due to the exceptional importance of HA for the human beings, its properties have been thoroughly investigated by many research groups and further studies are kept going. Namely, the crystal structure of HA is well described elsewhere (Elliott 1994; White and Dong 2003; Mathew and Takagi 2001), the detailed analysis of the electronic structure, bonding, charge transfer, optical and elastic properties is also available (Calderin et al. 2003; Rulis et al. 2004; Snyders et al. 2007; Ching et al. 2009), while the readers interested in Posner’s clusters are referred to still other papers (Treboux et al. 2000; Yin and Stott 2003; Kanzaki et al. 2001). A shell model was developed to study the lattice dynamics of HA (Calderin et al. 2005), while a cluster growth model was created to illustrate its growth (Onuma and Ito 1998). Polarization characteristics (Tanaka et al. 2010a, b), pyroelectric (Tofail et al. 2009, 2015) and piezoelectric (Tofail et al. 2015; Bystrov 2015) properties of HA as well as diffusion of protons inside the HA crystal lattice (Yashima et al. 2014) were investigated. The thermodynamic properties of HA and other types of orthophosphate-based apatites were summarized (Drouet 2015). First-principles calculations for the elastic properties of doped HA (Kawabata and Yamamoto 2010) and vacancy formation in HA (Matsunaga and Kuwabara 2007) were performed. Other examples of the computer simulations of the structure and properties of HA are available elsewhere (de Leeuw 2010; Corno et al. 2010; Slepko and Demkov 2011; Aquilano et al. 2014, 2015). Finally, an attempt was performed to explain an array of the curious characteristics of HA by referring to the hydroxyl ion channels extending in the direction of the *c*-axis, through a crystallographic column created by the overlapping calcium ion triangles (Uskoković 2015).

Many techniques might be utilized for HA preparation; they can be divided into solid-state reactions and wet methods (Briak-Ben et al. 2008), which include precipitation, hydrothermal synthesis and hydrolysis of other CaPO_4_. However, in all cases, Ca- and PO_4_-containing chemicals must be mixed to get the Ca/P ratio strictly equal to 1.67. Nevertheless, even under the ideal stoichiometric conditions, the precipitates are generally non-stoichiometric, suggesting intermediate formation of precursor phases, such as ACP and CDHA. Usually, unsintered HA is poorly crystalline and often non-stoichiometric, resembling the aforementioned CDHA. However, well crystalline HA can be prepared from aqueous solutions at relatively high (10–11) pH and elevated (>90 °C) temperatures (Markovic et al. 2004). HA with the Ca/P ratio >1.67 (Ca-rich HA) might be prepared as well (Bonel et al. 1988). The detailed information on HA synthesis is available elsewhere (Narasaraju and Phebe 1996; Riman et al. 2002; Koutsopoulos 2002; Norton et al. 2006; Sadat-Shojai et al. 2013). In addition, there are good reviews on HA solubility, crystal growth and intermediate phases of HA crystallization (Rakovan 2002), as well as on HA dissolution (Dorozhkin 2012e).

Pure HA never occurs in biological systems. However, due to the chemical similarities to bone and teeth mineral (Table 3), HA is widely used as coatings on orthopedic (e.g., hip joint prosthesis) and dental implants (Dorozhkin 2013c; Suchanek and Yoshimura 1998; Sun et al. 2001; Ong and Chan 1999; Dey et al. 2009; Dorozhkin 2015a). HA bioceramics is very popular as well (Yuan et al. 2009; Engin and Tas 1999; Mangano et al. 2008). Due to a great similarity to biological apatite, over a long time HA has been used in liquid chromatography of nucleic acids, proteins and other biological compounds (Bernardi 1965; Brand et al. 2008; Hou et al. 2011; Hilbrig and Freitag 2012; Niimi et al. 2014; Pinto et al. 2014) and for drug delivery purposes (Uskoković and Uskoković 2011; Chen et al. 2011a; Li et al. 2013; Long et al. 2013; Feng et al. 2013a). Also, HA is added to some brands of toothpaste as a gentle polishing agent instead of calcium carbonate (Niwa et al. 2001; Kim et al. 2006). Non-biomedical applications of HA include its using as an environmental-friendly filler for elastomers (Pietrasik et al. 2008), a low-temperature sorbent (Bailliez et al. 2004; Corami et al. 2008) and/or stabilizer (Wang et al. 2014b) of poisonous chemical elements, a high-temperature sorbent for carbon dioxide (Landi et al. 2014), both a catalyst (Xu et al. 2010; Rodrigues et al. 2014) and a carrier for other catalysts (Domínguez et al. 2009; Sun et al. 2009; Vukomanović et al. 2012), a material for ultraviolet light protection (Holzmann et al. 2009) and sunscreen filter (Piccirillo et al. 2014), as well as a component of various sensors (Nagai et al. 1988; Petrucelli et al. 1996; Tagaya et al. 2010; Khairnar et al. 2011). Finally, highly flexible and nonflammable inorganic paper could be prepared from HA (Lu et al. 2014a).

### FA (or FAp)

Fluorapatite [Ca_5_(PO_4_)_3_F, but is usually written as Ca_10_(PO_4_)_6_F_2_ to denote that the crystal unit cell comprises two molecules; the IUPAC name is pentacalcium fluoride tris(orthophosphate)] is the only ion-substituted CaPO_4_, considered in this review. Since the presence of 2.5 % of fluorides in natural apatites was established by 1798 (Dobson 1798), this date might be accepted as the earliest hearing of FA.

**Fig. 8:**
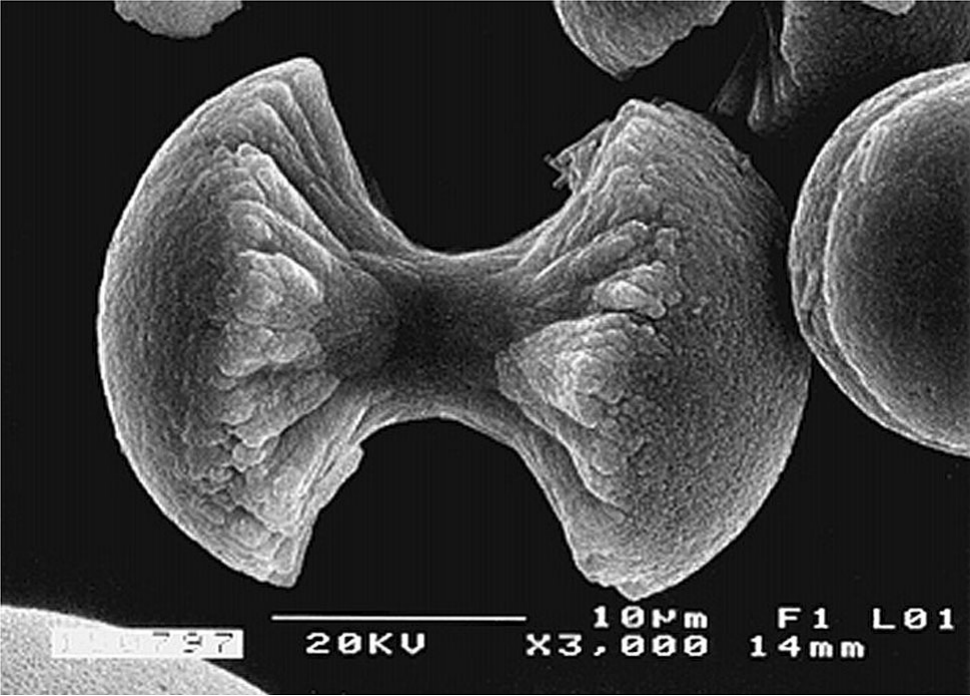
A biomimetically grown aggregate of FA that was crystallized in a gelatin matrix. Its shape can be explained and simulated by a fractal growth mechanism. *Scale bar* 10 μm. Reprinted from Ref. (Busch et al. 1999) with permission

FA is the hardest (five according to the Mohs’ scale of mineral hardness), most stable and least soluble compound among all CaPO_4_ (Table 1). In addition, it is the most thermally stable CaPO_4_ with the melting point at ~1650 °C (Tõnsuaadu et al. 2012). Perhaps, such “extreme” properties of FA are related to the specific position of F^−^ ions in the center of Ca(2) triangles of the crystal structure (Elliott 1994; White and Dong 2003). Due to its properties, FA is the only CaPO_4_ that naturally forms large deposits suitable for the commercial use (McConnell 1973; Becker 1989; Rakovan and Pasteris 2015; see also Fig. 2). Preparation techniques of the chemically pure FA are similar to the aforementioned ones for HA but the synthesis must be performed in presence of the necessary amount of F^−^ ions (usually, NaF or NH_4_F is added). Under some special crystallization conditions (e.g., in presence of gelatin or citric acid), FA might form unusual dumbbell-like fractal morphology that finally are closed to spheres (Fig. 8; Busch et al. 1999; Wu et al. 2010). In addition, FA is the only CaPO_4_, which melts without decomposition; therefore, big (up to 30 cm long and, for shorter lengths, up to 1.9 cm wide) single FA crystals might be grown from FA melts (Mazelsky et al. 1968a; Loutts and Chai 1993). Similar to that for HA (see CDHA), an existence of CaF_2_-deficient FA was also detected but for the crystals grown from the FA melt only (Mazelsky et al. 1968a; Warren 1972). In addition, FA with an excess of CaF_2_ was prepared (Mann and Turner 1972). A hierarchical structure for FA was proposed (Dorozhkin 2007a). The crystal structure of FA for the first time was studied in 1930 (Mehmel 1930; Naray-Szabo 1930) and is well described elsewhere (Elliott 1994; White and Dong 2003; Mathew and Takagi 2001). Computer simulations of the FA structure were performed as well (Li et al. 2015). The detailed analysis of the electronic structure, bonding, charge transfer and optical properties of FA (Rulis et al. 2004), as well as its NMR study under pressure (Pavan et al. 2012) is available as well. In addition, there are reviews on FA solubility (Rakovan 2002) and the dissolution mechanism (Dorozhkin 2012e).

FA easily forms solid solutions with HA with any desired F/OH molar ratio. Such compounds are called fluorhydroxyapatites (FHA) (Nikcevic et al. 2004; Montazeri et al. 2010; Zhu et al. 2012) or hydroxyfluorapatites (HFA) (Rodríguez-Lorenzo et al. 2003; Azami et al. 2012) and described with a chemical formula Ca_10_(PO_4_)_6_(OH)_2−*x*_F_*x*_, where 0 < *x* < 2. If the F/OH ratio is either uncertain or not important, the chemical formula of FHA and HFA is often written as Ca_10_(PO_4_)_6_(F,OH)_2_. The lattice parameters, crystal structure, solubility and other properties of FHA and HFA lay in between of those for the chemically pure FA and HA. Namely, the substitution of F for OH results in a contraction in the *a*-axis with no significant change in the *c*-axis dimensions and greater resolution of the IR absorption spectra.

Similar to pure HA, pure FA never occurs in biological systems. Obviously, a lack of the necessary amount of toxic fluorides (the acute toxic dose of fluoride is ~5 mg/kg of body weight) in living organisms is the main reason of this fact (pure FA contains 3.7 % mass. F). Enameloid of shark teeth (Lowenstam and Weiner 1989; Daculsi et al. 1997; Prostak et al. 1993; Dahm and Risnes 1999; Carr et al. 2006; Enax et al. 2014) and some exoskeletons of mollusks (Leveque et al. 2004) seem to be the only exclusions because they contain substantial amounts of fluoride, with is presented there as ion-substituted, non-stoichiometric FHA or HFA. Among all normal calcified tissues of humans, the highest concentration of fluorides is found in dentine and cementum, while the lowest—in dental enamel (Table 3). Nevertheless, one should stress that the amount of fluorides on the very surface of dental enamel might be substantially increased using fluoride-containing toothpastes and mouthwashes (Schemehorn et al. 2011; Hattab 2013). However, in no case, the total amount of fluorides is enough to form pure FA.

Contrary to the initial expectations (Heling et al. 1981), chemically pure FA is not used for grafting purposes. Presumably, this is due to the lowest solubility, good chemical stability of FA and toxicity of high amounts of fluorides. However, attempts to test FA-containing formulations (Gineste et al. 1999; Agathopoulos et al. 2003; Yoon et al. 2005; Bogdanov et al. 2009; Nordquist et al. 2011), ion-substituted FA (Kheradmandfard et al. 2012; Sharifnabi et al. 2014), FHA (Savarino et al. 2003; Vitkovič et al. 2009) and porous FA bioceramics (Chaari et al. 2009) are kept performing. The effect of fluoride contents in FHA on both osteoblast behavior (Qu and Wei 2006; Bhadang et al. 2010) and leukemia cells proliferation (Theiszova et al. 2008) has been described. Non-biomedical applications of FA include luminescent light tubes (Davis et al. 1971) and thermometers (Fu et al. 2015), laser materials (Mazelsky et al. 1968b; Ohlmann et al. 1968) (in all these cases various dopants are necessary), as well as catalysts (An et al. 2009).

### OA (or OAp, or OXA)

Oxyapatite [Ca_10_(PO_4_)_6_O; the IUPAC name is decacalcium oxide hexakis(phosphate), mineral voelckerite] is the least stable and, therefore, the least known CaPO_4_, which, probably, does not exist at all. Nevertheless, a name “voelckerite” was introduced in 1912 by A.F. Rogers (1887–1957) for a hypothetical mineral with the chemical composition of 3Ca_3_(PO_4_)_2_ + CaO (Rogers 1912, 1914), to honor an English agricultural chemist John Christopher Augustus Voelcker (1822–1884), who, in 1883, first showed an apparent halogen deficiency in some natural apatites (Voelcker 1883). Therefore, 1883 might be accepted as the earliest hearing on OA.

To the best of my findings, phase pure OA has never been obtained at room temperatures; therefore, its properties are not well established. Furthermore, still there are serious doubts that pure OA can exist. Since hydroxyl ions in HA appear to be the most mobile ones and upon exposure to high temperatures are the first to leave the lattice, a mixture (or a solid solution?) of OA and HA (so-called “oxy-HA”, chemical formula: Ca_10_(PO_4_)_6_(OH)_2−2x_O_x_V_x_, where V represents an OH^−^ vacancy) might be prepared by a partial dehydroxylation of HA at temperatures exceeding ~900 °C (e.g., during plasma spray of HA) strictly in the absence of water vapor (Gross et al. 1998; Hartmann et al. 2001; Alberius-Henning et al. 2001; Wang and Dorner-Reisel 2004; Liu and Shen 2012). It also might be crystallized in glass-ceramics (van’t Hoen et al. 2007). OA is very unstable and has no stability field in aqueous conditions (Duff 1972). Namely, data are available that oxy-HA containing less than 25 % HA (i.e., almost OA) during further dehydration decomposes to a mixture of α-TCP and TTCP. In addition, OA is very reactive and transforms to HA in contact with water vapor (HA reconstitution) (Gross et al. 1998). The largest impediment to active research in OA is the non-availability of a user-friendly approach to measure the concentration of hydroxyl ions (Gross and Pluduma 2012).

Therefore, computer-modeling techniques have been employed to qualitatively and quantitatively investigate the dehydration of HA to OA (de Leeuw et al. 2007). OA has the hexagonal space group symmetry *P* $\overline{6}$ (174) of cesanite type (White and Dong 2003), while the space group symmetry for partially dehydrated HA was found to change from hexagonal P63/m to triclinic *P* $\bar{1}$ when more than ca. 35 % of the structurally bound water had been removed (Alberius-Henning et al. 2001). On the *c*-axis, pure OA should have a divalent ion O^2−^ coupled with a vacancy instead of two neighboring monovalent OH^−^ ions.

Due to the aforementioned problems with OA preparation, it cannot be found in biological systems. In addition, no information on the biomedical applications of OA is available either. Plasma-sprayed coatings of CaPO_4_, in which OA might be present as an admixture phase, seem to be the only exception (Dorozhkin 2015a).

To conclude, one should mention that various types of calcium peroxy-HA (oxygenated HA) could be prepared both in the presence of hydrogen peroxide (Rey et al. 1978) and by HA heating in an oxygen-rich atmosphere (Zhao et al. 2000; Yu et al. 2007). Similar to that for OA, none of them was ever prepared as a phase pure individual compound and no examples of the biomedical applications of such types of CaPO_4_ were found.

### TTCP (or TetCP)

Tetracalcium phosphate or tetracalcium diorthophosphate monoxide (Ca_4_(PO_4_)_2_O; the IUPAC name is tetracalcium oxide bis(orthophosphate); the mineral hilgenstockite) is the most basic CaPO_4_, however, its solubility in water is higher than that of HA (Table 1). TTCP has been known since 1883 (Hilgenstock 1883), while the mineral hilgenstockite was named to honor a German metallurgist Gustav Hilgenstock (1844–1913), who first discovered it in Thomas slag from blast furnaces (Hilgenstock 1883, 1887). Its major industrial importance stems from the fact that it is formed by the reactions between orthophosphates and lime in the manufacture of iron, and through these reactions, TTCP has a significant role in controlling the properties of the metal.

TTCP cannot be precipitated from aqueous solutions. It can be prepared only under the anhydrous conditions by solid-state reactions at temperatures above ~1300 °C, e.g., by heating homogenized equimolar quantities of DCPA and CaCO_3_ in dry air, or in a flow of dry nitrogen (Elliott 1994, 1998; Kai et al. 2009). These reactions should be carried out in a dry atmosphere, in vacuum or with rapid cooling (to prevent uptake of water and formation of HA). Easily DCPA might be replaced by ammonium orthophosphates (Romeo and Fanovich 2008; Jalota et al. 2005), while calcium carbonate might be replaced by calcium acetate (Jalota et al. 2005); however, in all cases, Ca/P ratio must be equal to 2.00. Furthermore, TTCP often appears as an unwanted by-product in plasma-sprayed HA coatings, where it is formed as a result of the thermal decomposition of HA to a mixture of high-temperature phases of α-TCP, TTCP and CaO (Moseke and Gbureck 2010). Nevertheless, TTCP might be produced at 900 °C by calcining of a precipitated ACP with Ca/P = 2 in vacuum; the process is suggested to occur via a intermediate formation of a mixture of OA + CaO (Gross and Rozite 2015).

TTCP is metastable: in both wet environment and aqueous solutions it slowly hydrolyses to HA and calcium hydroxide (Elliott 1994, 1998; Martin and Brown 1993). Consequently, TTCP is never found in biological calcifications. In medicine, TTCP is widely used for preparation of various self-setting CaPO_4_ formulations (Dorozhkin 2013b; Moseke and Gbureck 2010); however, to the best of my knowledge, there is no commercial bone-substituting product consisting solely of TTCP. For the comprehensive information on TTCP, the readers are referred to a special review (Moseke and Gbureck 2010), while the spectra (Jillavenatesa and Condrate 1997) and solubility (Pan and Darvell 2009a) of TTCP are well described elsewhere.

To finalize the description of individual CaPO_4_, one should mention on an interesting opinion, that all types of CaPO_4_ listed in Table 1 might be classified into three major structural types (Mathew and Takagi 2001; Chow and Eanes 2001). They comprise: (1) the apatite type, Ca_10_(PO_4_)_6_X_2_, which includes HA, FA, OA, CDHA, OCP and TTCP; (2) the glaserite type, named after the mineral glaserite, K_3_Na(SO_4_)_2_, which includes all polymorphs of TCP and, perhaps, ACP; (3) the Ca-PO_4_ sheet-containing compounds, which include DCPD, DCPA, MCPM and MCPA. According to the authors, a closer examination of the structures revealed that all available CaPO_4_ could be included into distorted glaserite type structures, but with varying degrees of distortion (Mathew and Takagi 2001; Chow and Eanes 2001).

### Biphasic, triphasic and multiphasic CaPO_4_ formulations

CaPO_4_ might form biphasic, triphasic and multiphasic (polyphasic) formulations, in which the individual components cannot be separated from each other. Presumably, the individual phases of such compositions are homogeneously “mixed” at a far submicron level (<0.1 μm) and strongly integrated with each other. Nevertheless, the presence of all individual phases is easily seen by X-ray diffraction technique (Dorozhkin 2012d).

The usual way to prepare multiphasic formulations consists of sintering non-stoichiometric compounds, such as ACP and CDHA, at temperatures above ~700 °C. Furthermore, a thermal decomposition of the stoichiometric CaPO_4_ at temperatures above ~1300 °C might be used as well; however, this approach often results in formation of complicated mixtures of various products including admixtures of CaO, calcium pyrophosphates, etc. (Dorozhkin 2012d; Nakano et al. 2002b). Namely, transformation of HA into polyphasic CaPO_4_ by annealing in a vacuum occurs as this: the outer part of HA is transformed into α-TCP and TTCP, while the α-TCP phase of the surface further transforms into CaO. Besides, in the boundary phase, HA is transformed into TTCP (Nakano et al. 2002b).

Historically, Nery and Lynch with co-workers first used the term biphasic calcium phosphate (BCP) in 1986 to describe a bioceramic that consisted of a mixture of HA and β-TCP (Ellinger et al. 1986). Based on the results of X-ray diffraction analysis, these authors found that the “tricalcium phosphate” preparation material used in their early publication (Nery et al. 1975) was in fact a mixture of ~20 % HA and ~80 % β-TCP. Currently, only biphasic and triphasic CaPO_4_ formulations are known; perhaps, more complicated formulations will be manufactured in future. Furthermore, nowadays, multiphasic and/or polyphasic compositions consisting of high-temperature phases of CaPO_4_, such as α-TCP, β-TCP, HA and, perhaps, high-temperature ACP, OA and TTCP, are known only. No precise information on multiphasic compositions, containing MCPM, MCPA, DCPD, DCPA, low-temperature ACP, OCP and CDHA has been found in literature (Dorozhkin 2012d). Perhaps, such formulations will be produced in future.

All BCP formulations might be subdivided into two major groups: those consisting of CaPO_4_ having either the same (e.g., α-TCP and β-TCP) or different (e.g., β-TCP and HA) molar Ca/P ratios. Among all known BCP formulations, BCP consisting of HA and β-TCP is both the most known and the best investigated (Dorozhkin 2012d). In 1986, LeGeros in USA and Daculsi in France initiated the basic studies on preparation of this type of BCP and its in vitro properties. This material is soluble and gradually dissolves in the body, seeding new bone formation as it releases calcium and orthophosphate ions into the biological medium. Presently, commercial BCP products of different or similar HA/β-TCP ratios are manufactured in many parts of the world as bone graft or bone substitute materials for orthopedic and dental applications under various trademarks and several manufacturers (Dorozhkin 2012d). A similar combination of α-TCP with HA forms BCP as well (Reid et al. 2005; Pan et al. 2006; Kui 2007; Li et al. 2009b).

Recently, the concept of BCP has been extended by preparation and characterization of biphasic TCP (BTCP), consisting of α-TCP and β-TCP phases (Wang et al. 2006a; Li et al. 2007, 2008a). It is usually prepared by heating ACP precursors (Wang et al. 2006a; Li et al. 2007, 2008a), in which the α-TCP/β-TCP ratio can be controlled by aging time and pH value during synthesis of the amorphous precursor (Li et al. 2007). Furthermore, triphasic formulations, consisting of HA, α-TCP and β-TCP (Vani et al. 2009) or HA, α-TCP and TTCP (Nakano et al. 2002b) have been prepared (Dorozhkin 2012d).

It is important to recognize, that the major biomedical properties (such as bioactivity, bioresorbability, osteoconductivity and osteoinductivity) of the multiphasic formulations might be adjusted by changing the ratios among the phases. When compared to both α- and β-TCP, HA is a more stable phase under the physiological conditions, as it has a lower solubility (Table 1) and, thus, slower resorption kinetics. Therefore, due to a higher biodegradability of the α- or β-TCP component, the reactivity of BCP increases with the TCP/HA ratio increasing. Thus, in vivo bioresorbability of BCP can be adjusted through the phase composition. Similar conclusions are also valid for both the biphasic TCP (in which α-TCP is a more soluble phase) and the triphasic (HA, α-TCP and β-TCP) formulations. Further details on this subject might be found in a topical review (Dorozhkin 2012d).

### Ion-substituted CaPO_4_

Last, one should very briefly mention on existence of carbonated HA (Lafon et al. 2008; Tonegawa et al. 2010; Yahia and Jemal 2010; Silvester et al. 2014; Fleet 2015), chlorapatite (Kannan et al. 2007; García-Tuñón et al. 2012; Zhao et al. 2013), as well as on a great number of CaPO_4_ with various ionic substitutions (CaPO_4_ with dopants) (Pan and Fleet 2002; Rey et al. 2006; Kannan et al. 2008; Boanini et al. 2010b; Shepherd et al. 2012; Adzila et al. 2012; Grigg et al. 2014; Šupová 2015). In principle, any ion in CaPO_4_ might be substituted by other ion(s). Usually, the ion-substituted CaPO_4_ are of a non-stoichiometric nature with just a partial ionic substitution and there are too many of them to be described here. Currently, this is a hot investigation topic; therefore, the readers are referred to the special literature (Pan and Fleet 2002; Rey et al. 2006; Kannan et al. 2008; Boanini et al. 2010b; Shepherd et al. 2012; Adzila et al. 2012; Grigg et al. 2014; Šupová 2015). In addition, there is a very good review, in which the structures of more than 75 chemically different apatites have been discussed (White and Dong 2003).

To finalize this brief topic of ion-substituted CaPO_4_, it is important to note that chemical elements not found in natural bones can be intentionally incorporated into CaPO_4_ biomaterials to get special properties. For example, addition of Ag^+^ (Pushpakanth et al. 2008; Zhang et al. 2010b; Kim et al. 1998; Kolmas et al. 2014), Zn^2+^ (Kim et al. 1998; Kolmas et al. 2014; Stanić et al. 2010) and Cu^2+^ (Kim et al. 1998; Kolmas et al. 2014; Stanić et al. 2010; Li et al. 2010) was used for imparting antimicrobial effect, while radioactive isotopes of ^90^Y (Thomas et al. 2008), ^153^Sm (Chinol et al. 1993; Argüelles et al. 1999; O’Duffy et al. 1999) and ^186^Re (Chinol et al. 1993) were incorporated into HA bioceramics and injected into knee joints to treat rheumatoid joint synovitis (Thomas et al. 2008; Chinol et al. 1993; O’Duffy et al. 1999). More to the point, apatites were found to incorporate individual molecules, such as water, oxygen and carbon dioxide (Rey et al. 2006).

Finally, to conclude the description of the known CaPO_4_, one should mention that in spite of the well-defined crystallographic data (Table 4) and, therefore, the well-defined shapes of the single crystals, various types of CaPO_4_ can be prepared with the controllable sizes from nano- to macro-scale (up to centimeter size) and the diverse shapes including zero- (particles), one- (rods, fibers, wires and whiskers), two- (sheets, disks, plates, belts, ribbons and flakes) and three-dimensional morphologies (Sadat-Shojai et al. 2013; Lin et al. 2014). The latter might be of versatile morphologies and shapes (Fig. 8 is an example), including porous and hollow structures.

## Biological hard tissues of CaPO_4_

Biological mineralization (or biomineralization) is the process of in vivo formation of inorganic minerals (so-called, biominerals). One should stress that the term “biomineral” refers not only to a mineral produced by organisms but also to the fact that almost all of these mineralized products are composite materials comprised both inorganic and bioorganic components. Furthermore, having formed in vivo under well-controlled conditions, the biomineral phases often have properties, such as shape, size, crystallinity, isotopic and trace element compositions, quite unlike its inorganically formed counterpart (please, compare Figs. 2, 8, 10 and 15 bottom). Thus, the term “biomineral” reflects all this complexity (Lowenstam and Weiner 1989).

As shown in Table 3 and discussed above, in the body of mammals, the vast majority of both normal and pathological calcifications consist of non-stoichiometric and ion-doped CaPO_4_, mainly of apatitic structure (Pasteris et al. 2008; Palmer et al. 2008). At the atomic scale, nano-sized crystals bone apatite exhibit a variety of substitutions and vacancies that make the Ca/P molar ratio distinct from the stoichiometric HA ratio of 1.67. Their chemical composition is complicated and varies in relatively wide ranges. This depends on what the animal has ingested (Grynpas et al. 1993). Occasionally, attempts are performed to compose chemical formulas of biological apatites. For example, the following formula Ca_8.856_Mg_0.088_Na_0.292_K_0.010_(PO_4_)_5.312_(HPO_4_)_0.280_(CO_3_)_0.407_(OH)_0.702_Cl_0.078_(CO_3_)_0.050_ was proposed to describe the chemical composition of the inorganic part of dental enamel (Elliott 2002).

The presence of impurities in the biological apatite of calcified tissues introduces significant stresses into the crystal structure, which make it less stable and more reactive. Among all substituting ions, the presence of 4–8 % of carbonates instead of orthophosphate anions (so-called, B-type substitution (LeGeros 1991; Elliott 1994; Amjad 1997; Lafon et al. 2008)) and of 0.5–1.5 % of Mg is of the special importance because it leads to large lattice strain and significantly increases the solubility (Palmer et al. 2008; Elliott 2002; Boskey 2006). Higher concentrations of Mg and carbonates in bone or dentine compared to those in enamel (Table 3) may explain a higher solubility, a lower crystallinity and smaller crystal dimensions of bone or dentine compared to enamel.

**Fig. 9:**
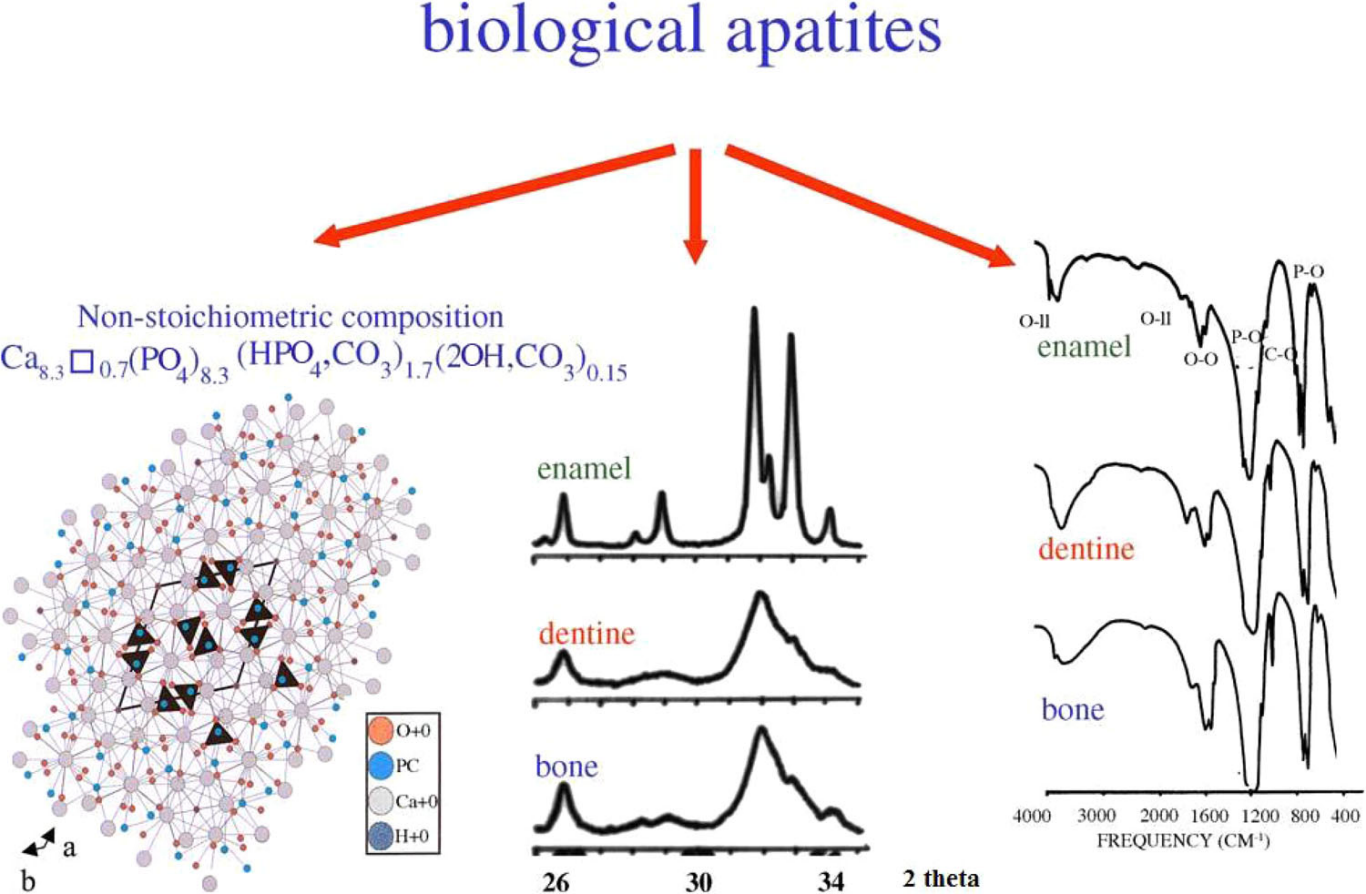
*Left* crystal structure of a biological apatite. Powder X-ray diffraction patterns (*center*) and infrared spectra (*right*) of human enamel, dentine and bone. Reprinted from Ref. (Vallet-Regí and González-Calbet 2004) with permission

In addition, the crystals of biological apatite are always very small which also increases its solubility when compared with that for the chemically pure HA and even CDHA (Rey et al. 2006). However, biologic apatites of enamel have considerably larger both crystal sizes (about 2000 nm) and crystallite dimensions compared to those of either bone or dentine apatite. The substantial differences in crystallite dimensions of biological apatites of different origin are clearly seen as the well-defined X-ray diffraction peaks of enamel apatite and much broader diffraction peaks of either bone or dentine apatites (Fig. 9, center). Small dimensions and a low crystallinity are two distinct features of biological apatites, which, combined with their non-stoichiometric composition, inner crystalline disorder and presence of other ions in the crystal lattice, allow explaining their special behavior. For example, the small crystal size means that a large percentage of the atoms are on the surface of the crystals, providing a large specific surface area for sorption of ions, proteins and drugs (Boskey 2006; Vallet-Regí and González-Calbet 2004). The major physical properties of biological apatite are summarized in Fig. 9. It is interesting to note, that the solubility and equilibrium phenomena of CaPO_4_ related to the calcification process have been studied, at least, since 1925 (Holt et al. 1925a, b).

To the best of my findings (Dorozhkin 2012a, 2013a), the first attempts to mimic the CaPO_4_ nature of bones were performed in 1913 (Gassmann 1913). This discovery was clarified afterwards, suggesting that the bone mineral could be carbonated apatite (de Jong 1926; Bredig 1933). Further optical and X-ray analysis of bones and other mineralized tissues matched analyses of two apatites: FA and dahllite (Taylor and Sheard 1929). Additional historical data on this point are available in literature (Dorozhkin 2012a, 2013a). In 1998, Weiner and Wagner wrote the following: “the term bone refers to a family of materials, all of which are built up of mineralized collagen fibrils” (Weiner and Wagner 1998; Weiner et al. 1999). Therefore, for mammals, this family of materials includes dentine—the material that constitutes the inner layers of teeth, cementum—the thin layer that binds the roots of teeth to the jaw, deer antlers and some other materials. It is worth noting, that bones and teeth contain almost 99 % of the total body calcium and about 85 % of the total body phosphorus that amounts to a combined mass of approximately 2 kg in an average person. In addition, it is important to recognize that CaPO_4_ of bones are by no means inert; they play an important role in the metabolic functions of the body. The data on the physico-chemical and crystallographic study of biological apatite are available elsewhere (Elliott 2002).

### Bone

Bone, also called osseous tissue (Latin: *os*), is a type of hard endoskeletal connective tissue found in many vertebrate animals. All bones of a single animal are, collectively, known as the skeleton. True bones are present in bony fish (osteichthyes) and all tetrapods. Bones support body structures, protect internal organs and, in conjunction with muscles, facilitate movement (Loveridge 1999). In addition, bones are also involved with blood cell formation, calcium metabolism and act for mineral storage. For a material scientist, bone is a dynamic, highly vascularized solid tissue that is formed from a complicated biocomposite containing both inorganic (Table 3) and bioorganic (chiefly, collagen) compounds, in which nanodimensional crystals of the inorganic phases are dispersed in the meshes of the bioorganic ones (Palmer et al. 2008; Nightingale and Lewis 1971; Currey 2002; Rho et al. 1998; Tzaphlidou 2008). More than 20 types of collagen have been reported in the human body, among which type I collagen is the most abundant protein and provides much of the structural integrity for connective tissue, particularly in bones, tendons and ligaments. Furthermore, for a physiologist, bone is a living organ populated by living cells (mainly, osteoblasts, osteoclasts and osteocytes); however, that is another story. The inorganic to bioorganic ratio is approximately 75–25 % by dry weight and about 65–35 % by volume. This ratio not only differs among animals, among bones in the same animal and over time in the same animal but also it exerts a major control on the material properties of bone, such as its toughness, ultimate strength and stiffness. In general, load-bearing ability of bones depends on not only architectural properties, such as cortical thickness and bone diameter, but also intrinsic, size-independent, material properties such as porosity, level of mineralization, crystal size and properties derived from the organic phase of bone (Davison et al. 2006). A higher mineral to collagen ratio typically yields stronger, but more brittle, bones (Turner and Burr 1993; Currey 2004; Currey et al. 2004). For example, bones from the leg of a cow have a relatively high concentration of CaPO_4_ (for support), whereas bones from the antler of a deer have a relatively high concentration of collagen (for flexibility) (Pan and Darvell 2009a). It is interesting to note that bones exhibit several physical properties such as piezoelectricity (Anderson and Eriksson 1970) and pyroelectricity (Lang 1966).

Stability of the mineral composition of bones has a very long history: CaPO_4_ were found in dinosaur fossils (Elorza et al. 1999; Eagle et al. 2010; Haynes 1968; Rensberger and Watabe 2000; Kolodny et al. 1996; Trueman and Tuross 2002). Therefore, organisms have had a great deal of time to exploit the feedback between composition and structure in apatite, on the one hand, and benefit from its biological functionality, on the other. Bones of the modern animals are a relatively hard and lightweight porous composite material, formed mostly of biological apatite (i.e., poorly crystalline CDHA with ionic substitutions). It has relatively high compressive strength but poor tensile strength (Currey and Brear 1990). While bones are essentially brittle, they have a degree of significant plasticity contributed by their bioorganic components.

**Fig. 10:**
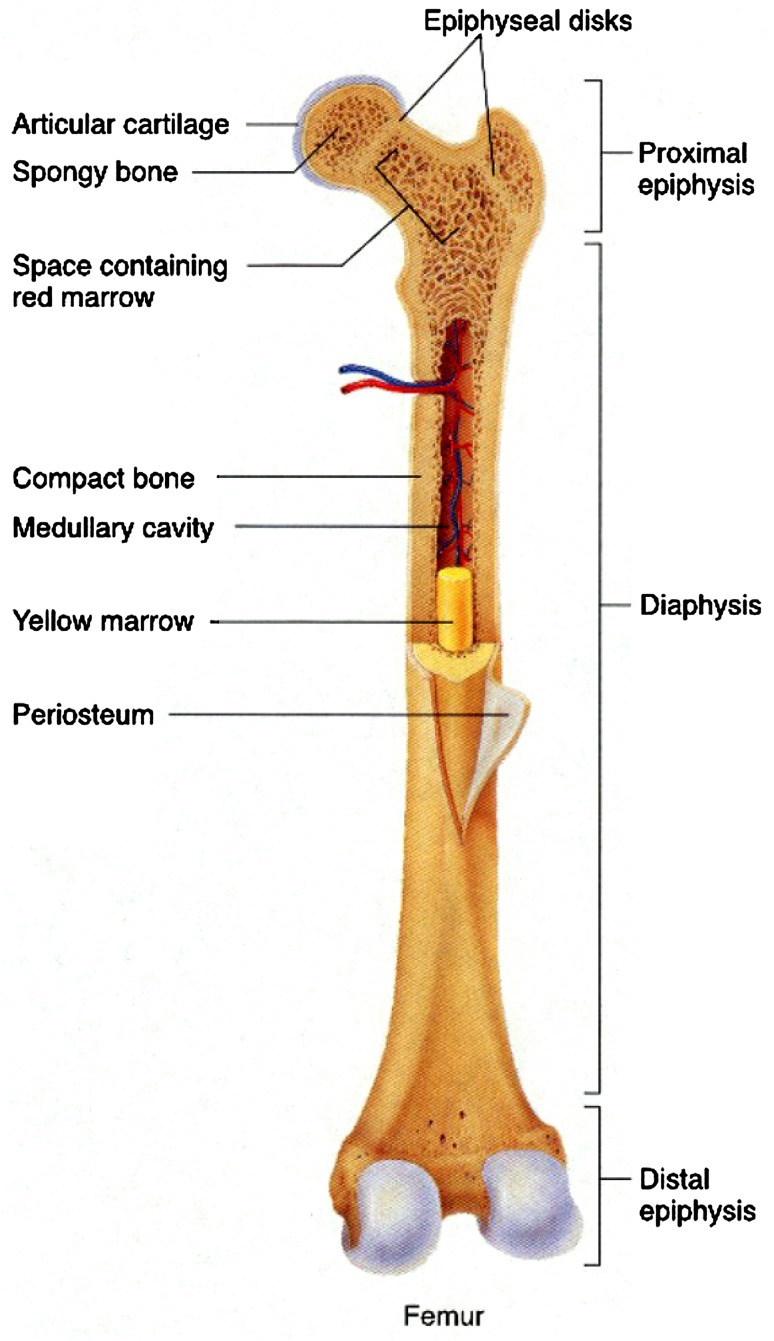
General structure of a mammalian bone. Other very good graphical sketches of the mammalian bone structure are available in Refs. (Pasteris et al. 2008; Vallet-Regí and González-Calbet 2004)

The distribution of the inorganic and bioorganic phases depends on a highly complex process that takes place during bone formation. Each of these components may be assembled in different proportions creating two different architectural structures depending on the bone type and function. They are characterized by different structural features that strongly correlate with the mechanical performance of the tissue. These two types of bones are: the cortical bones (or compact bones) which are dense and the cancellous bones (also known as trabecular or spongious bones) which are less dense and less stiff than the compact ones. Usually, bones are composed of a relatively dense outer layer of cortical bone covering an internal mesh-like structure (average porosity of 75–95 %) of cancellous bone, the density of which is about 0.2 g/cm^3^ but it may vary at different points (Fig. 10). Cortical bones make up a large portion of the skeletal mass; they have a high density (~1.80 g/cm^3^) and a low surface area. Cancellous bones have an open meshwork or honeycomb-like structure. They have a relatively high surface area but form a smaller portion of the skeleton. Bones are a porous material with the pore sizes ranging from 1 to 100 μm in normal cortical bones and 200–400 μm in trabecular ones. 55–70 % of the pores in trabecular bones are interconnected. The porosity reduces the strength of bones but also reduces their weight (LeGeros 1991, 2001; Lowenstam and Weiner 1989; O’Neill 2007; Skinner 2005; Daculsi et al. 1997; Weiner and Wagner 1998; Currey 2002; Rho et al. 1998; Rey et al. 2009; Lerebours et al. 2015).

**Fig. 11:**
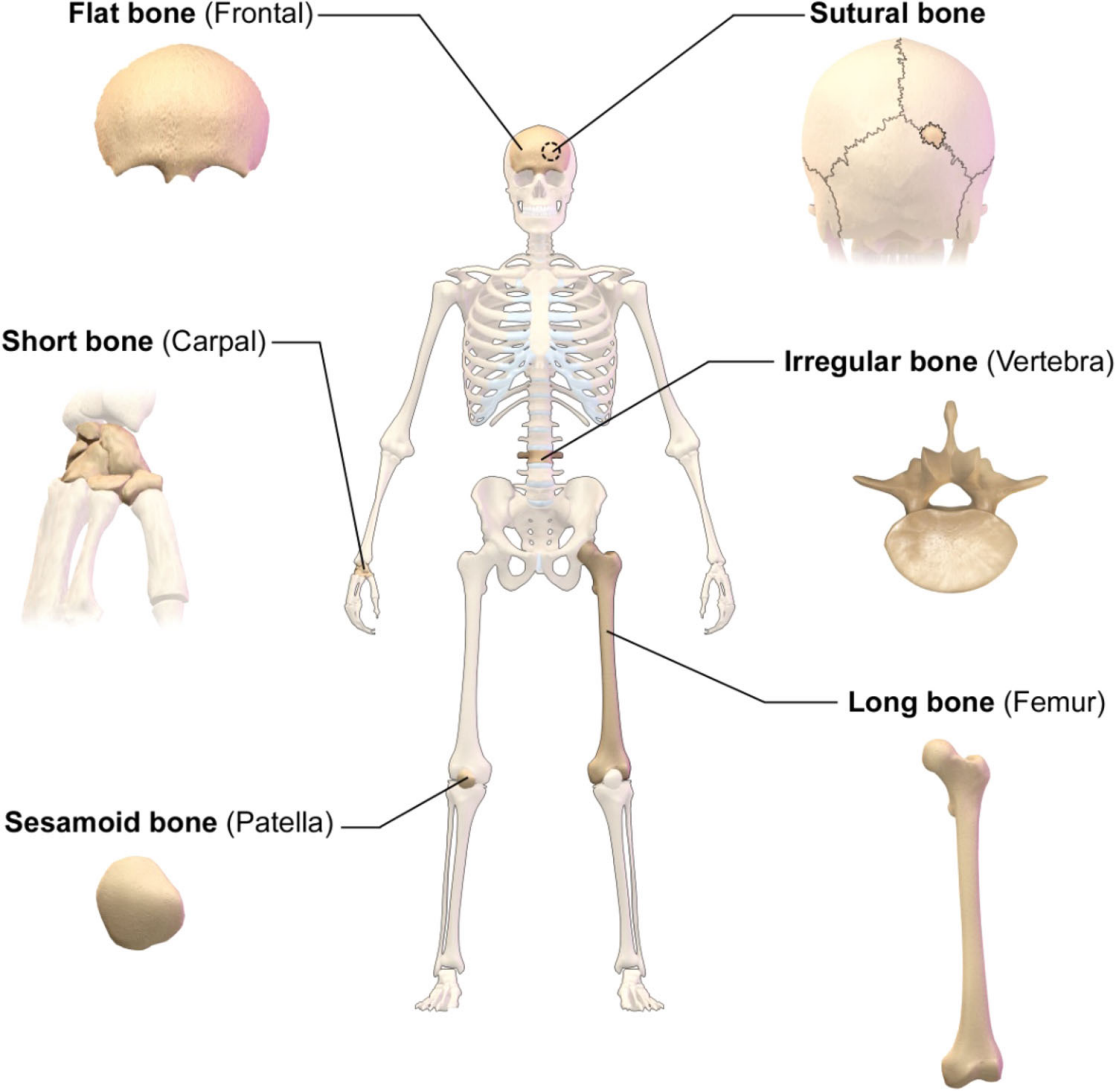
Classification of bones by shape

**Fig. 12:**
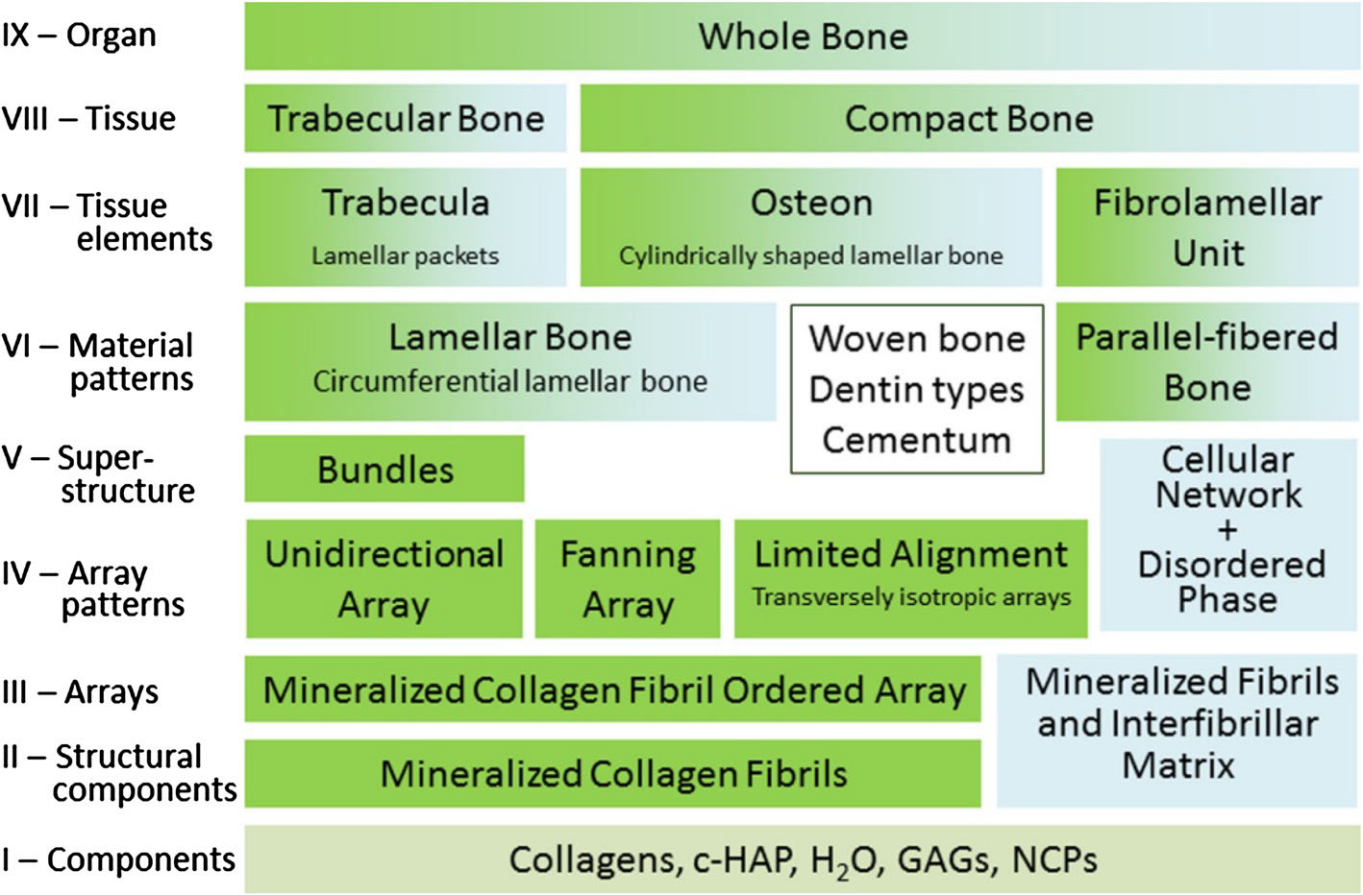
A schematic illustration of the hierarchical organization of bones. Up to level V, the hierarchical levels can be divided into the ordered material (*green*) and the disordered material (*blue*). At level VI, these two materials combine in lamellar bone and parallel fibered bone. Other members of the bone family still need to be investigated with respect to the presence of both material types; hence, they are depicted in a *box* without *color*. Level VII depicts the lamellar packets that make up trabecular bone material and the cylindrically shaped lamellar bone that makes up osteonal bone. The fibrolamellar unit comprises the primary hypercalcified layer, parallel fibered bone and lamellar bone. *c-HAP* carbonated HA, *GAGs* glycosaminoglycans, *NCPs* non-collagenous proteins. Reprinted from Ref. (Reznikov et al. 2014b) with permission. Other good graphical sketches of the hierarchical structure of bones are available in Refs. (Weiner and Wagner 1998; Cui et al. 2007; Boskey and Coleman 2010; Meyers et al. 2008)

Bones can be either woven or lamellar. The fibers of woven bones are randomly aligned and as the result have a low strength. In contrast, lamellar bones have parallel fibers and are much stronger. Woven bones are put down rapidly during growth or repair (Watt 1925) but as growth continues, they are often replaced by lamellar bones. The replacement process is called “secondary bone formation” and described in details elsewhere (Olszta et al. 2007). In addition, bones might be long, short, flat, sutural, sesamoid and irregular (Fig. 11). The sizes and shapes of bones reflect their function. Namely, broad and flat bones, such as scapulae, anchor large muscle masses, flat skull bones protect the brain, ribs protect the lungs, pelvis protects other internal organs, short tubular bones in the digits of hands and feet provide specific grasping functions, hollow and thick-walled tubular bones, such as femur or radius, support weight and long bones enable locomotion (Boskey 2007; Glimcher 2006). Long bones are tubular in structure (e.g., the tibia). The central shaft of a long bone is called the diaphysis and has a medullar cavity filled with bone marrow (Fig. 10). Surrounding the medullar cavity is a thin layer of cancellous bone that also contains marrow. The extremities of the bone are called the epiphyses and are mostly cancellous bone covered by a relatively thin layer of compact bone. Short bones (e.g., finger bones) have a similar structure to long bones, except that they have no medullar cavity. Flat bones (e.g., the skull and ribs) consist of two layers of compact bone with a zone of cancellous bone sandwiched between them. Irregular bones (e.g., vertebrae) do not conform to any of the previous forms. Thus, bones are shaped in such a manner that strength is provided only where it is needed. All bones contain living cells embedded in a mineralized organic matrix that makes up the main bone material (Boskey 2007; Glimcher 2006; Boskey and Roy 2008). The structure of bones is most easily understood by differentiating between seven levels of organization because bones exhibit a strongly hierarchical structure (Palmer et al. 2008; Weiner and Wagner 1998; Nightingale and Lewis 1971; Currey 2002, 2005; Rho et al. 1998; Rensberger and Watabe 2000; Cui et al. 2007; Fratzl and Weinkamer 2007; Boskey and Coleman 2010; Meyers et al. 2008; Reznikov et al. 2014a, b). One should stress that, taking into account the presence of ordered and disordered materials, nine hierarchical levels for lamellar bones have been differentiated (Fig. 12; Reznikov et al. 2014b).

The mechanical properties of bones reconcile high stiffness and high elasticity in a manner that is not yet possible with synthetic materials (Meyers et al. 2008). Cortical bone specimens have been found to have tensile strength in the range of 79–151 MPa in longitudinal direction and 51–56 MPa in transversal direction. Bone’s elasticity is also important for its function giving the ability to the skeleton to withstand impact. Estimates of modulus of elasticity of bone samples are of the order of 17–20 GPa in longitudinal direction and of 6–13 GPa in the transversal direction (Athanasiou et al. 2000). The elastic properties of bone were successfully modeled at the level of mineralized collagen fibrils via step-by-step homogenization from the staggered arrangement of collagen molecules up to an array of parallel mineralized fibrils (Nikolov and Raabe 2008). The investigations revealed that bone deformation was not homogeneous but distributed between a tensile deformation of the fibrils and a shearing in the interfibrillar matrix between them (Gupta et al. 2005; Peterlik et al. 2006). Readers, who are interested in further details, are addressed to a good review on the effects of the microscopic and nano-scale structure on bone fragility (Ruppel et al. 2008).

**Fig. 13:**
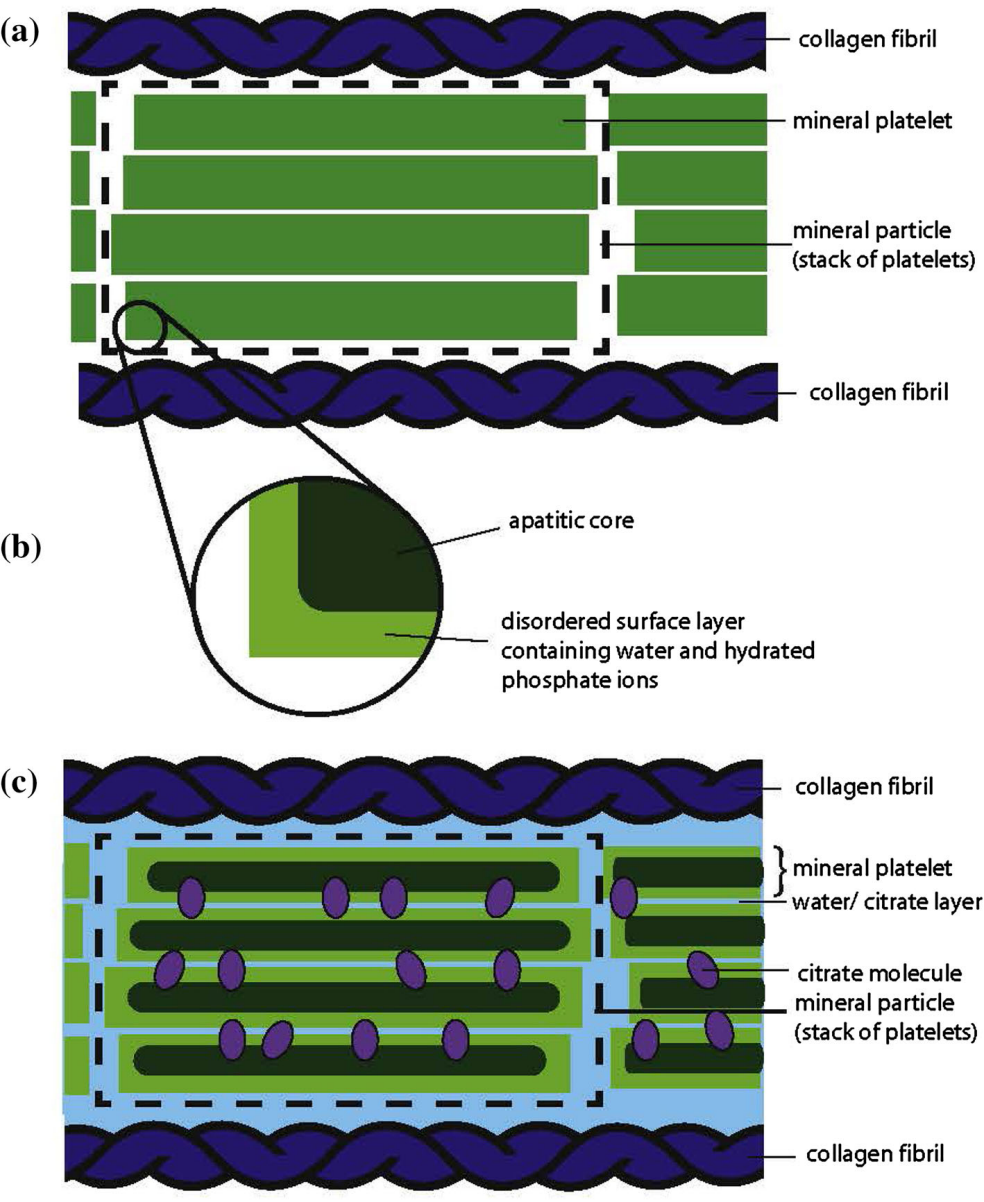
**a** A schematic view of the current model of the organic–inorganic composite nanostructure of bone. Polycrystalline particles of biological apatite, consisting of stacks of (single crystal) mineral platelets are sandwiched into the space between collagen fibrils. The large platelet (100) faces are parallel to each other and the platelet *c*-axis is strongly ordered with the collagen fibril axis. **b** A schematic view of the structure of a single mineral platelet, with an atomically ordered core resembling the CDHA structure (with substitutions) surrounded by a surface layer of disordered, hydrated mineral ions. **c** A schematic view of the detailed structural model of bone mineral showing how citrate anions and water bind the mineral platelets together. Reprinted from Ref. (Duer 2015) with permission

The smallest level of the bone hierarchy consists of the molecular components: water, biological apatite, collagen and other proteins (Fig. 13; Duer 2015). The second smallest hierarchical level is formed by mineralization of collagen fibrils, which are of 80–100 nm thickness and a length of a few to tens of microns (Fig. 12). Thus, biocomposites of biological apatite and molecules of type I collagen are formed (Pasteris et al. 2008; Weiner and Wagner 1998; Nightingale and Lewis 1971; Tzaphlidou 2008; Fratzl et al. 2004). Some evidences for direct physical bonding between the collagen fibers and apatite crystals in bone were found (Marino and Becker 1967). Atomic force microscopy was used to measure the crystals of biological apatite in mature cow bones (Eppell et al. 2001). The crystals are always platelet like (elongated along the crystallographic *c*-axis) and very thin (Wopenka and Pasteris 2005; Clark and Iball 1954; Rubin et al. 2003; Su et al. 2003), with remarkably uniform thicknesses (determined in transmission electron microscopy) of 2–4 nm (i.e., just a few unit cells thick—see Table 3). They exist in bones not as discrete aggregates but rather as a continuous phase, which is indirectly evidenced by a very good strength of bones. This results in a very large surface area facing extracellular fluids, which is critically important for the rapid exchange of ions with these fluids. The nano-sized crystals of biological apatite are inserted in a nearly parallel way into the collagen fibrils, while the latter are formed by self-assembly of collagen triple helices (Weiner and Wagner 1998; Nightingale and Lewis 1971; Cui et al. 2007; Landis et al. 1996; Rosen et al. 2002) using the self-organization mechanism (Sato 2006; Hartgerink et al. 2001). In addition, experimental data from electron diffraction studies revealed that that the mineral plates of biological apatite are not quite as ordered as previously assumed (Olszta et al. 2007). This imperfect arrangement of nearly parallel crystals has been supported by SAXS and transmission electron microscopy studies (Burger et al. 2008). According to the latest data, water, which always present in bone mineral, appears to glue the mineral platelets together in a way akin to how water sticks two glass plates together—it provides firm binding between the plates, but at the same time allows slippage between them—flexibility for a stack of mineral platelets held together in this way (Duer 2015).

The lowest level of hierarchical organization of bone has successfully been simulated by CDHA precipitation on peptide-amphiphile nanodimensional fibers (Hartgerink et al. 2001). However, apatite platelets nucleating on the surface of peptide tubules are not similar to the nanostructure of bone and they are only an example of surface induced nucleation (and not accurately characterized either), while the nanostructure of bone consists of intra-fibrillar platelets intercalated within the collagen fibrils. However, the interface between collagen and biological apatite is still poorly understood; for the available details, the readers are referred to a review devoted to the structure and mechanical quality of the collagen/mineral nanodimensional biocomposite of bones (Fratzl et al. 2004). There is still no clear idea why the crystals of biological apatite are platelet shaped even though dahllite has hexagonal crystal symmetry (Weiner and Wagner 1998; Currey 2002; Rho et al. 1998; Rey et al. 2009). One possible reason is that they grow via an OCP transition phase, in which crystals are plate-shaped (Weiner and Wagner 1998). Another explanation involves the presence of citrates, which strongly bound to (10Ī0) surface of biological apatite because of space matching (Hu et al. 2010; Xie and Nancollas 2010). Therefore, the crystal growth in the [10Ī0] direction becomes inhibited, while the citrate effect on other crystal surfaces of biological apatite appears to be very small owing to poor space matching. Thus, after crystal growth, the (10Ī0) crystal face becomes predominant resulting in plate-like morphology of biological apatite (Xie and Nancollas 2010).

The processes of bone formation (ossification) and growth are very complicated ones and it is difficult to describe them without making a deep invasion into biology. It has been studied for decades (Watt 1925) but still there are missing points. From the standpoint of chemists, formation of bones could be considered as calcification (i.e., deposition or precipitation of CaPO_4_) within the bioorganic matrix of connective tissues (mainly cartilage), resulting in formation of biocomposites (Palmer et al. 2008; Olszta et al. 2007). Cartilage is composed of collagen fibers, cells (chondrocytes and their precursor forms known as chondroblasts) and extracellular matrix (proteoglycans, which are a special class of heavily glycosylated glycoproteins). Very briefly, the initial stage of ossification involves synthesis and extracellular assembly of the collagen matrix framework of fibrils. At the second stage, chondrocytes calcify the matrix before undergoing the programmed cell death (apoptosis). At this point, blood vessels penetrate this calcified matrix, bringing in osteoblasts (they are mononuclear cells primarily responsible for bone formation), which use the calcified cartilage matrix as a template to build bone, and ions of calcium and orthophosphate to be deposited in the ossifying tissue (Hall 2015). Therefore, the role of collagen in the nucleation, growth, structure and orientation of apatite crystals appears to be predominant (Wang et al. 2012). The biomineralization process is controlled to some extent by cells and the organic matrices made by those cells facilitate the deposition of crystals (Boskey and Roy 2008). As bone crystals grow, there is greater association with proteins, such as osteocalcin, that regulate remodeling (George and Veis 2008). Thus, in vivo formation of hard tissues always occurs by mineral reinforcement of the previously formed network of soft tissues (Palmer et al. 2008; Olszta et al. 2007; Boskey 2007; Glimcher 2006; Cui et al. 2007).

Since the bioorganic matrix has control over the size and orientation of CaPO_4_ crystals, the latter grow with a specific crystalline orientation—the *c*-axes of the crystals are roughly parallel to the long axes of the collagen fibrils within which they are deposited (Palmer et al. 2008; Grynpas et al. 1993; Weiner and Wagner 1998; Currey 2002; Rho et al. 1998; Tzaphlidou 2008; Olszta et al. 2007). Earlier, it was believed that this process occurred via epitaxial growth mechanism (Marino and Becker 1970). The same was suggested for dentine and enamel (Jodaikin et al. 1988; Fincham et al. 1995) (see “Teeth”), as well as for more primitive living organisms. For example, in the shell of the fossil marine animal *Lingula brachiopod unguis* that consists of a biological apatite, the crystal *c*-axes are oriented parallel to the β-chitin fibrils (Leveque et al. 2004; Williams et al. 1998; Rohanizadeh and LeGeros 2007; Neary et al. 2011). Therefore, the orientation of biological apatite crystals parallel to the long axes of the organic framework could be a general feature of the CaPO_4_ biomineralization. However, the degree of the crystal orientation appears to be a useful parameter to evaluate an in vivo stress distribution, a nano-scale microstructure and a related mechanical function, a regenerative process of the healed bone, as well as to diagnose bone diseases such as osteoarthritis (Nakano et al. 2007, 2008). It is interesting to note that contrary to what might be expected in accordance with possible processes of dissolution, formation and remineralization of hard tissues, no changes in phase composition of mineral part, crystal sizes (length, width and thickness) and arrangement of crystals on collagen fibers were detected in abnormal (osteoporotic) human bones compared to the normal ones (Suvorova et al. 2007).

Some animals, such as newts, are able to regenerate amputated limbs. This is, of course, of a high interest for the regenerative medicine. Therefore, bone regeneration in the forelimbs of mature newts was studied by noninvasive X-ray microtomography to image regenerating limbs from 37 to 85 days. The missing limb skeletal elements were restored in a proximal-to-distal direction, which reiterated the developmental patterning program. However, in contrast to this proximal–distal sequence, the portion of the humerus distal to the amputation site was found to fail to ossify in synchrony with the regenerating radius and ulna. This finding suggests that the replacement of cartilage with mineralized bone close to the amputation site is delayed with respect to other regenerating skeletal elements (Stock et al. 2003).

Unlike other mineralized tissues, bone continuously undergoes a remodeling process, as it is resorbed by specialized cells called osteoclasts and formed by another type of cells called osteoblasts (so-called “bone lining cells”) in a delicate equilibrium to ensure that there are major net changes in neither bone mass nor its mechanical strength (Palmer et al. 2008; Olszta et al. 2007; Boskey and Roy 2008; Teitelbaum 2000; Rodan and Martin 2000). The purpose of remodeling is the release of calcium and the repair of micro-damaged bones from everyday stress. Osteoblasts are mononuclear cells primarily responsible for bone formation. They contain alkaline phosphatase, which enzymatically produces orthophosphate anions needed for the mineralization. In addition, there is one more type of the cells called osteocytes that originate from osteoblasts, which have migrated into, become trapped and surrounded by bone matrix, which they themselves produce (Palmer et al. 2008; Currey 2002; Olszta et al. 2007; Boskey 2007; Glimcher 2006; Boskey and Roy 2008; Weiner et al. 2005).

If osteoblasts are bone-forming cells, osteoclasts are multinuclear, macrophage-like cells, which can be described as bone-destroying cells because they mature and migrate to discrete bone surfaces (Boskey and Roy 2008; Teitelbaum 2000; Rodan and Martin 2000). Upon arrival, active enzymes, such as acid phosphatase, are secreted to produce lactic acid that causes dissolution of biological apatite. This process, called bone resorption, allows stored calcium to be released into systemic circulation and is an important process in regulating calcium balance (Teitelbaum 2000; Rodan and Martin 2000). The iteration of remodeling events at the cellular level is influential on shaping and sculpting the skeleton both during growth and afterwards. That is why, mature bones always consist of a very complex mesh of bone patches, each of which has both a slightly different structure and a different age (Palmer et al. 2008; Grynpas et al. 1993; Elliott 2002; Weiner and Wagner 1998; Currey 2002; Rho et al. 1998; Olszta et al. 2007). The interested readers are suggested to read a review on the interaction between biomaterials and osteoclasts (Schilling et al. 2006).

Still there is no general agreement on the chemical mechanism of bone formation. It is clear that the inorganic part of bone consists of biological apatite, i.e., CDHA with ionic substitutions but without the detectable amounts of hydroxide (Rey et al. 1995a; Loong et al. 2000; Seo et al. 2007). However, the results of solid-state nuclear magnetic resonance on fresh-frozen and ground whole bones of several mammalian species revealed that the bone crystal OH^−^ was readily detectable; a rough estimate yielded an OH^−^ content of human cortical bone of about 20 % of the amount expected in stoichiometric HA (Cho et al. 2003). Various in vitro experiments on precipitation of CDHA and HA revealed that none of these compounds is directly precipitated from supersaturated aqueous solutions containing calcium and orthophosphate ions: some intermediate phases (precursors) are always involved (LeGeros 1991, 2001; Elliott 1994; O’Neill 2007; Iijima et al. 1996; Bodier-Houllé et al. 1998; Rodríguez-Hernández et al. 2005; Tomazic et al. 1994; Nancollas and Wu 2000; Dorozhkin 2012c). Depending on both the solution pH and crystallization conditions, three types of CaPO_4_ compounds (DCPD, ACP and OCP) have been discussed as possible precursors of CDHA precipitation in vitro. Due to this reason, the same CaPO_4_ are suggested as possible precursors of biological apatite formation in vivo.

The transient nature of the precursor phase of bone, if it exists at all, makes it very difficult to detect, especially in vivo (Grynpas and Omelon 2007). However, in 1966 Brown proposed that OCP was the initial precipitate that then acted as a template upon which biological apatite nucleates (Brown 1966). This idea was extended in his further investigations (Tung and Brown 1983, 1985; Brown et al. 1987; Siew et al. 1992). The principal support for this concept derived from the following: (1) the close structural similarity of OCP and HA (Brown 1962; Brown et al. 1962); (2) formation of interlayered single crystals of OCP and HA (pseudomorphs of OCP); (3) the easier precipitation of OCP compared with HA; (4) the apparent plate- or lath-like habit of biological apatites that does not conform to hexagonal symmetry, but looks like a pseudomorph of triclinic OCP; (5) the presence of HPO_4_^2−^ in bone mineral, particularly in newly formed bones (Elliott 2002). Some evidences supporting this idea were found using high-resolution transmission electron microscopy: computer-simulated lattice images of the “central dark line” in mineralized tissues revealed that it consisted of OCP (Brown 1966; Bodier-Houllé et al. 1998; Rodríguez-Hernández et al. 2005). In addition, Raman spectroscopic indications for an OCP precursor phase were found during intra-membranous bone formation (Crane et al. 2006). Other evidences of OCP to HA transformation, including a mechanistic model for the central dark line formation, might be found in literature (Tseng et al. 2006).

Simultaneously with Brown, the research group led by Posner proposed that ACP was the initially precipitated phase of bone and dentine mineral formation in vivo, thus explaining the non-stoichiometric Ca/P ratio in bones and teeth (Eanes et al. 1965; Termine and Posner 1966a, b). This conclusion was drawn from the following facts: (1) when CaPO_4_ are prepared by rapid precipitation from aqueous solutions containing ions of calcium and orthophosphate at pH > 8.5, the initial solid phase is amorphous; (2) mature bone mineral is composed of a mixture of ion-substituted ACP and poorly crystallized ion-substituted CDHA; (3) early bone mineral has a lower crystallinity than mature bone and the observed improvement in crystallinity with the age of the bone mineral is a result of a progressive reduction in the ACP content (Elliott 2002; Eanes et al. 1965; Termine and Posner 1966a, b; Harper and Posner 1966; Posner 1969, 1973, 1978; Boskey and Posner 1976; Glimcher et al. 1981). Interestingly, thermodynamic data prove that the transition of freshly precipitated ACP into CDHA involves intermediate formation of OCP (Meyer and Eanes 1978a, b). However, the discovery of a stable amorphous calcium carbonate in sea urchin spines (Politi et al. 2004) reawakened the suggestion that a transient amorphous phase might also exist in bones (Olszta et al. 2007; Weiner et al. 2005, 2009; Weiner 2006; Pekounov and Petrov 2008; Gower 2008). Afterwards, evidences of an abundant ACP phase in the continuously forming fin bones of zebrafish were found (Mahamid et al. 2008, 2010). The new bone mineral was found to be delivered and deposited as packages of nanodimensional spheres of ACP, which further transformed into platelets of crystalline apatite within the collagen matrix (Mahamid et al. 2010).

**Fig. 14:**
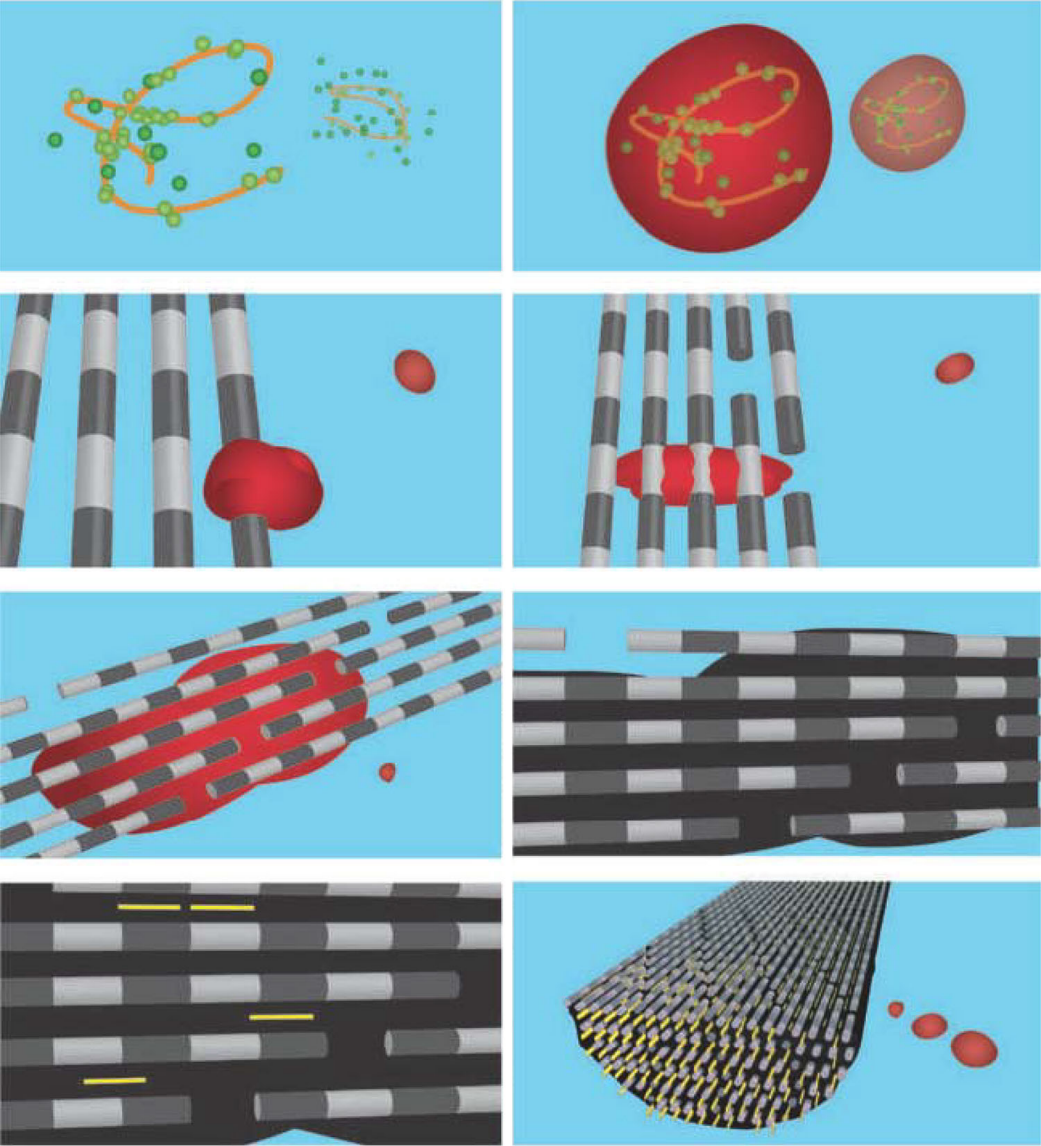
A schematic illustration of in vivo mineralization of a collagen fibril: *top layer*—CaPO_4_ clusters (*green*) form complexes with biopolymers (*orange line*), forming stable mineral droplets; *second top layer*—mineral droplets bind to a distinct region on the collagen fibers and enter the fibril; *second bottom layer*—once inside the collagen, the mineral in a liquid state diffuses through the interior of the fibril and solidifies into a disordered phase of ACP (*black*); *bottom layer*—finally, directed by the collagen, ACP is transformed into oriented crystals of biological apatite (*yellow*). Reprinted from Ref. (Cölfen 2010) with permission

Furthermore, to investigate how apatite crystals form inside collagen fibrils, researchers carried out a time-resolved study starting from the earliest stages of mineral formation (Nudelman et al. 2010). After 24 h of mineralization, CaPO_4_ particles were found outside the fibril, associated with the overlap region, in close proximity to the gap zone. Cryogenic energy-dispersive X-ray spectroscopy confirmed that these precipitates were composed of CaPO_4_, while a low-dose selected-area electron diffraction technique showed a diffuse band characteristic of ACP. After 48 h, CDHA crystals started to develop within a bed of ACP and after 72 h, elongated electron-dense crystals were abundant within the fibril, in many cases still embedded within a less dense matrix. A low-dose selected-area electron diffraction technique demonstrated that the mineral phase consisted of both ACP and oriented apatite, the latter identical to bone apatite (Nudelman et al. 2010). This process is schematically shown in Fig. 14 (Cölfen 2010). Further details on the bone formation mechanisms are available in literature (Olszta et al. 2007; Hall 2015; Driessens et al. 2012; Boonrungsiman et al. 2012), where the interested readers are referred.

In spite of the century-plus long studies (Dorozhkin 2012a, 2013a), the maturation mechanism of bone minerals remains to be not well established, mainly due to the difficulties involved in the nanostructural analyses (Olszta et al. 2007; Sahar et al. 2005). Still, indirect evidences for the in vivo bone mineral maturation are available only. For example, X-ray diffraction patterns of bones from animals of different age show that the reflections become sharper with age increasing (Meneghini et al. 2003; Bilezikian et al. 2008). This effect is more pronounced in the crystallographic *a*-axis [(310) reflections] as compared to the *c*-axis [(002) reflections] (Burnell et al. 1980; Weiner and Traub 1986). The most comprehensive report describing how normal human bone mineral changes in composition and crystal size as a function of age was based on X-ray diffraction analyses by Hanschin and Stern (Hanschin and Stern 1995), who examined 117 homogenized iliac crest biopsies from patients aged 0–95 years. They found that the bone mineral crystal size and perfection increased during the first 25–30 years and then decreased thereafter, slightly increasing in the oldest individuals. The same 117 homogenized biopsy samples were analyzed by wavelength-dispersive X-ray fluorescence to quantify the carbonate substitution in biological apatite as a function of age. Although the changes observed in carbonate substitution were relatively slight (at most 10 %), there was a general increase from 0 to 90 years that is distinct from the absence of a change in crystallinity after age 30 in these samples (Boskey and Coleman 2010). In addition, other changes, like an increase of Ca^2+^ content and a decrease of HPO_4_^2−^, occur in bone mineral with age (Verdelis et al. 2007; Yerramshetty et al. 2006; Kuhn et al. 2008; Donnelly et al. 2009; Li and Pasteris 2014; Lowenstam 1981; Lowenstam and Weiner 1983; Plate et al. 1996; Stratmann et al. 1996; Jahnen-Dechent et al. 1997, 2001; Schinke et al. 1999; Savelle and Habu 2004; Chen et al. 2011b; Delloye et al. 2007; Araújo et al. 2009; Koussoulakou et al. 2009; Jones 2001; Avery 2001; Nanci 2012; Xue et al. 2008; Gaft et al. 1996; Huang et al. 2007; Yin et al. 2013; Ho et al. 2009a). Both the crystal sizes and carbonate content were found to increase during aging in rats and cows (LeGeros et al. 1987; Rey et al. 1995b). The increase in carbonate content with age was also reported in other studies (Yerramshetty et al. 2006; Kuhn et al. 2008; Donnelly et al. 2009; Li and Pasteris 2014). Simultaneously with carbonate content increasing, the content of sodium increased as well (Li and Pasteris 2014). From a chemical point of view, these changes indicate to a slow transformation of poorly crystallized non-apatitic CaPO_4_ into a better-crystallized ion-substituted carbonate-containing CDHA. In addition, during aging, edge areas of bones were found to become less porous, whereas the concentration of organics in the edges was reduced (Li and Pasteris 2014). While there are still many gaps in our knowledge, the researchers seem to be comfortable in stating that in all but the youngest bone and dentine, the only phase present is a highly disordered, highly substituted biological apatite.

In general, the biomineralization process (therefore, bone formation) can happen in two basic ways: either the mineral phase is developed from the ambient environment as it would from a supersaturated solution of the requisite ions, but just requires the living system to nucleate and localize mineral deposition, or the mineral phase is developed under the direct regulatory control of the organism, so that the mineral deposits are not only localized but may be directed to form unique crystal habits not normally developed by a saturated solution of the requisite ions. In a very famous paper (Lowenstam 1981) and two extended elaborations (Lowenstam and Weiner 1983, 1989), the first type of biomineralization was called “biologically induced” mineralization and the second “(organic) matrix-mediated” biomineralization. In some papers, the former process is called “passive” and the latter one—“active” biomineralization (Lowenstam and Weiner 1989). Briefly, an “active process” means an assembly of nano-sized crystals of biological apatite into bones due to an activity of the suitable cells (e.g., osteoblasts), i.e., within a matrix vesicle. Such structures have been discovered by transmission electron microscopy for bone and teeth formation (Plate et al. 1996; Stratmann et al. 1996). A “passive process” does not require involvement of cells and means mineralization from supersaturated solutions with respect to the precipitation of biological apatite. In the latter case, thermodynamically, biomineralization might occur at any suitable nucleus. The collagen fibrils have a specific structure with a 67 nm periodicity and 35–40 nm gaps or holes between the ends of the collagen molecules where bone mineral is incorporated in the mineralized fibril (Weiner and Wagner 1998; Weiner et al. 1999; Tzaphlidou 2008; Boskey 2007; Glimcher 2006). Such a nucleation within these holes would lead to discrete crystals with a size related to the nucleating cavity in the collagen fibril (Fig. 12). It was proposed that a temporary absence of the specific inhibitors might regulate the process of bone formation (Jahnen-Dechent et al. 1997, 2001; Schinke et al. 1999).

To conclude the bone subject, let me briefly mention on the practical application of bones. In the Stone Age, bones were used to manufacture art, weapons, needles, catchers, amulets, pendants, headdresses, etc. In the coastal regions, big bones of whales were used to construct houses (Savelle and Habu 2004). Nowadays, cut and polished bones from a variety of animals are sometimes used as a starting material for jewelry and other crafts. Ground cattle bones are used as a fertilizer (Chen et al. 2011b). Furthermore, in medicine, bones are used for bone graft substitutes, such allografts from cadavers (Delloye et al. 2007) and xenografts (Araújo et al. 2009). However, since recently, bone-derived biomaterials are attempting to avoid due to serious concerns about bovine spongiform encephalopathy (“mad cow disease”) and other possible diseases that might be transmitted even by heated bones.

### Teeth

Teeth (singular: tooth) are dense mineralized hard tissues found in the jaws, mouth and/or pharynx of many vertebrates (Koussoulakou et al. 2009). They have various structures to allow them to fulfill their different purposes. The primary function of teeth is to tear and chew food, while for carnivores it is also a weapon. Therefore, teeth have to withstand a range of physical and chemical processes, including compressive forces (up to ~700 N), abrasion and chemical attack due to acidic foods or products of bacterial metabolism (Jones 2001). The roots of teeth are covered by gums. From the surface teeth are covered by enamel of up to ~2 mm thick at the cutting edges of the teeth, which helps to prevent cavities on the teeth. The biggest teeth of some gigantic animals (elephants, hippopotamuses, walruses, mammoths, narwhals, etc.) are known as tusks or ivory.

Similar to the various types of bones, there are various types of teeth. The shape of the teeth is related to the animal’s food, as well as its evolutionary descent. For example, plants are hard to digest, so herbivores have many molars for chewing. Carnivores need canines to kill and tear and since meat is easy to digest, they can swallow without the need for molars to chew the food well. Thus, the following types of teeth are known: molars (used for grinding up food), carnassials (used for slicing food), premolars (small molars), canines (used for tearing apart food) and incisors (used for cutting food). While humans only have two sets of teeth, some animals have many more: for example, sharks grow a new set of teeth every 2 weeks. Some other animals grow just one set during the life, while teeth of rodents grow and wear away continually through the animal gnawing, maintaining constant length (Avery 2001; Nanci 2012).

Similar to bones, the inorganic part of teeth also consist of biological apatite (Xue et al. 2008). The stability of the mineral composition of teeth also has a very long history: namely, CaPO_4_ were found in fossil fish teeth (Gaft et al. 1996). Similarly, investigations of biological apatite from fossil human and animal teeth revealed its similarity to the modem biological apatite (Huang et al. 2007). The same was found for modern and fossil (mammoth) elephant ivories (Yin et al. 2013).

**Fig. 15:**
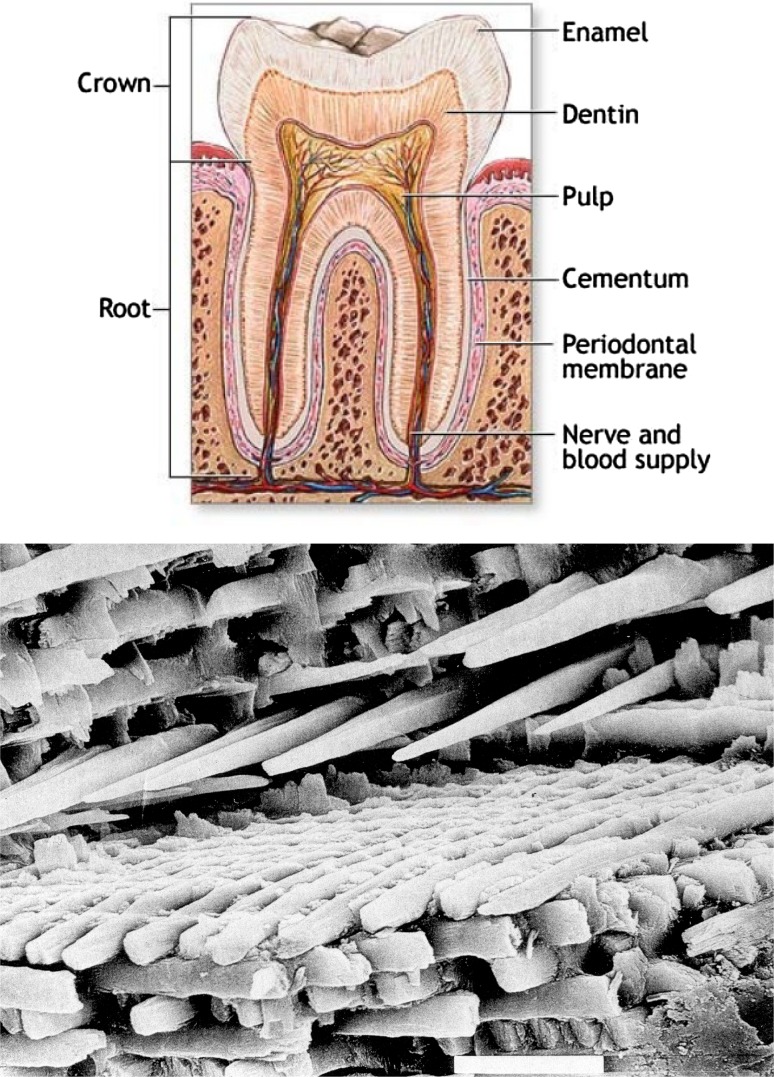
*Top* a schematic drawing of a tooth. Other very good graphical sketches of the mammalian tooth structure, including the hierarchical levels, are available in Refs. (Palmer et al. 2008; Meyers et al. 2008). *Bottom* a scanning electron micrograph of the forming enamel of a continuously growing rat incisor showing ordered rods of CaPO_4_. *Scale bar* 10 μm. Reprinted from Ref. (Lowenstam and Weiner 1989) with permission

The structure of teeth (Fig. 15 top) appears to be even more complicated than that of bones. Unlike bones, teeth consist of at least two different CaPO_4_ materials: enamel, which is a rigid, inert and acellular outer layer, and dentine, which is a bone-like Mg-rich hard tissue that forms the bulk of vertebrate teeth. In addition, there is cementum, which is a thin layer of a bone-like calcified tissue that covers dentine at the roots of teeth and anchors them to the jaw (Ho et al. 2009a, b; Bosshardt and Selvig 2000; Yamamoto et al. 2010). Finally, there is the core called pulp (commonly called “the nerve”)—it is a remnant of the embryologic organ for tooth development and contains nerves and blood vessels necessary for tooth function. All these tissues form a highly organized and complex structure (Fig. 15 top) with ideal functional and structural capabilities, which assist in sustaining mastication-induced mechanical loading and preventing their mechanical failures during function (Boskey 2007; Glimcher 2006; Avery 2001; Nanci 2012). It is interesting to note that the structure of teeth is common to the most species. Namely, fish, reptiles and mammals share the same architecture of a harder external layer and a tougher core.

Both dentine and cementum are mineralized connective tissues with an organic matrix of collagenous proteins, while the inorganic component of them consists of biological apatite. As shown in Table 3, dentine, cementum and bone are quite similar and for general purposes of material scientists they are regarded as being essentially the same material (Elliott 2002; Weiner and Wagner 1998; Currey 2002; Rho et al. 1998; Fratzl et al. 2004; Rubin et al. 2003; Su et al. 2003; Pellegrino and Blitz 1972; LeGeros et al. 1987; Jones 2001). However, strictly speaking, there are some differences among them. For example, the hardness of live dentine is less than that of enamel but is greater than that of bone or cementum (Ho et al. 2009a). When pulp of the tooth dies or is removed by a dentist, the properties of dentine change: it becomes brittle, liable to fracture and looses a reparative capability. In addition, unlike bones, both dentine and cementum lack vascularization. In spite of these minor differences, let us consider that the majority of the statements made in the previous section for bones are also valid for dentine and cementum.

Dental enamel is the outermost layer of teeth. It is white and translucent and its true color might be observed at the cutting edges of the teeth only. Enamel is highly mineralized and acellular, so it is not a living tissue. Nevertheless, it is sufficiently porous for diffusion and chemical reactions to occur within its structure, particularly acidic dissolution (dental caries) and remineralization from saliva (possible healing of caries lesions). Therefore, it acts as a selectively permeable membrane, allowing water and certain ions to pass via osmosis (Avery 2001; Nanci 2012).

Enamel is the hardest substance in the body and forms a solid, tough and wear-resistant surface for malaxation (Chai et al. 2009). In the mature state, it contains up to 98 % of inorganic phase (Table 3). Other components of enamel include remnants of the organic matrix and loosely bound water molecules. Interestingly, from the mechanical point of view, dental enamel was found to be a “metallic-like” deformable biocomposite, because enamel was found to have a similar stress–strain response to that of cast alloy and gold alloy, all of which showed work-hardening effect (He and Swain 2007). The crystals of biological apatite of enamel are much larger as evidenced by higher crystallinity (reflecting greater crystal size and perfection) demonstrated in their X-ray diffraction patterns than those of bone and dentine. Besides, enamel apatite has fewer ionic substitutions than bone or dentine mineral and more closely approximates the stoichiometric HA (Boskey 2007). The organic phase of enamel does not contain collagen. Instead, enamel has two unique classes of proteins called amelogenins and enamelins. While the role of these proteins is not fully understood yet, it is believed that both classes of proteins aid in the enamel development by serving as a framework support (Avery 2001; Nanci 2012; Margolis et al. 2006). The large amount of minerals in enamel accounts not only for its strength but also for its brittleness. Dentine, which is less mineralized and less brittle, compensates for enamel and is necessary as a support (Avery 2001; Nanci 2012). Shark enameloid is an intermediate form bridging enamel and dentine. It has enamel-like crystals of fluoridated biological apatite associated with collagen fibrils (Lowenstam and Weiner 1989; Rey et al. 2006; Prostak et al. 1993; Dahm and Risnes 1999; Carr et al. 2006; Enax et al. 2014). Due to the presence of fluorides, biological apatite of shark enameloid shows both higher crystal sizes and a more regular hexagonal symmetry if compared to non-fluoridated biological apatite of bones and teeth (Daculsi et al. 1997). Similar correlation between the presence of fluorides and crystal dimensions was found for enamel (Vieira et al. 2003).

Like that for bones, seven levels of structural hierarchy have been also discovered in human enamel; moreover, the analysis of the enamel and bone hierarchical structure suggests similarities of the scale distribution at each level (Palmer et al. 2008; Athanasiou et al. 2000; Cui and Ge 2007). On the mesoscale level, there are three main structural components: a rod, an interrod and aprismatic enamel. Among them, the enamel rod (formerly called an enamel prism) is the basic unit of enamel. It is a tightly packed mass of biological apatite in an organized pattern. Each rod traverses uninterrupted through the thickness of enamel. They number 5–12 million rods per crown. The rods increase in diameter (4 up to 8 microns) as they flare outward from the dentine-enamel junction (DEJ). Needle-like enamel rods might be tens of microns long (up to 100 µm) but sometimes only 50 nm wide and 30 nm thick (Fig. 15 bottom; Avery 2001; Nanci 2012; Jandt 2006; Chen et al. 2004; Rönnholm 1962; Nylen et al. 1963; Miake et al. 1993; Daculsi et al. 1984; Jodaikin et al. 1984; Bres and Hutchison 2002). They are quite different from the much smaller crystals of dentine and bone (Table 3), but all of them consist of biological apatite (Schroeder and Frank 1985; Brès et al. 1990). In cross section, an enamel rod is best compared to a keyhole, with the top, or head, oriented toward the crown of the tooth and the bottom, or tail, oriented toward the root of the tooth.

The arrangement of the crystals of biological apatite within each enamel rod is highly complex. Enamel crystals in the head of the enamel rod are oriented parallel to the long axis of the rod. When found in the tail of the enamel rod, the crystals’ orientation diverges slightly from the long axis (Avery 2001; Nanci 2012). The arrangement of the enamel rods is understood more clearly than their internal structure. Enamel rods are found in rows along the tooth (Fig. 15 bottom) and, within each row, the long axis of the enamel rod is generally perpendicular to the underlying dentine (Avery 2001; Nanci 2012; Jandt 2006; Chen et al. 2004; Rönnholm 1962; Nylen et al. 1963; Miake et al. 1993). An AFM study indicated that apatite crystals in enamel exhibited regular sub-domains or subunits with distinct chemical properties related to topographical features and gave rise to patterned behavior in terms of the crystal surface itself and the manner in which it responded to low pH (Robinson et al. 2004). In addition, the results of the investigations of human single apatite crystals of both enamel and dentine revealed an absence of the mirror plane perpendicular to the *c*-axis leading to the *P*6_3_ space group (Mugnaioli et al. 2014) instead of the *P*6_3_/m space group typical for HA and FA (Table 4).

The second structural component of the enamel matrix is the interrod (or interprismatic) enamel, which surrounds and packs between the rods. The difference between the rod and the interrod is the orientation of apatite crystals; the rod contains aligned crystallites, whereas the mineral in the interrod is less ordered. These structures coalesce to form the tough tissue of enamel, which can withstand high forces and resist damage by crack deflection. The third structure, aprismatic enamel, refers to the structures containing apatite crystals that show no meso-scale or macro-scale alignment (Palmer et al. 2008).

The in vivo formation and development of teeth appears to be even more complicated when compared with the aforedescribed process of bone formation. It is a very complex biological process, by which teeth are formed from embryonic cells, grow and erupt into the mouth (Boskey and Roy 2008). For human teeth enamel, dentine and cementum must all be developed during the appropriate stages of fetal development. Primary (baby) teeth start to form between the sixth and eighth weeks in utero, while the permanent teeth begin to form in the twentieth week in utero (Avery 2001; Nanci 2012). Experimental data confirmed the necessity of CaPO_4_ in the diet of pregnant and nursing mother to prevent early childhood dental caries (Warf and Watson 2008).

As teeth consist of at least two materials with different properties (enamel and dentine), the tooth bud (sometimes called “the tooth germ”—that is an aggregation of cells that eventually forms a tooth) is organized into three parts: the enamel organ, the dental papilla and the dental follicle. The enamel organ is composed of at least four other groups of cells [for the biological details see Refs. (Avery 2001; Nanci 2012)]. Altogether, these groups of cells give rise to ameloblasts, which secret enamel matrix proteins. The protein gel adjacent to ameloblasts is supersaturated with CaPO_4_, which leads to the precipitation of biological apatite. Similarly, the dental papilla contains cells that develop into odontoblasts, which are dentine-forming cells. The dental follicle gives rise to three important entities: cementoblasts, osteoblasts and fibroblasts. Cementoblasts form the cementum of a tooth (Bosshardt and Selvig 2000). Osteoblasts give rise to the alveolar bone around the roots of teeth (see bone formation above). Fibroblasts develop the periodontal ligaments that connect teeth to the alveolar bone through cementum (Boskey 2007; Glimcher 2006; Boskey and Roy 2008; Hall 2015; Avery 2001; Nanci 2012).

The first detectable crystals in enamel formation are flat thin ribbons (Rönnholm 1962; Nylen et al. 1963; Miake et al. 1993) that were reported to be OCP (Brown and Chow 1976; Simmer and Fincham 1995; Diekwisch et al. 1995; Aoba 1996), β-(Ca,Mg)_3_(PO_4_)_2_ (Diekwisch et al. 1995), DCPD (Rey et al. 1995a; Bonar et al. 1991) or ACP (Beniash et al. 2009). The formation process of enamel is different from that for bone or dentine: amelogenin being hydrophobic self-assembles into nano-sized spheres that guide the growth of the ribbon-like dental enamel crystals. During maturation of enamel, the mineral content increases from initially ~45 wt.% up to ~98–99 wt.% (Avery 2001; Nanci 2012; Bonar et al. 1991). The enamel crystal rods widen and thicken by additional growth (Rey et al. 1995b; Bonar et al. 1991; Smith 1998) with a simultaneous increase of the Ca/P molar ratio (Smith 1998) and a decrease in carbonate content (Sydney-Zax et al. 1991; Rey et al. 1991; Takagi et al. 1998), finally resulting in the most highly mineralized and hardest substance produced by vertebrates. It is interesting to note that in the radular teeth of chitons, ACP was found to be the first-formed CaPO_4_ mineral, which over a period of weeks was transformed to dahllite (Lowenstam and Weiner 1985).

The crystal faces expressed in enamel are always (100) face and at the ends presumably (001) (Selvig 1970, 1973), which are the ones usually found in HA. The centers of enamel crystals contain a linear structure known as the “central dark line” (this line was also observed in bone and dentine), which consists of OCP (Iijima et al. 1996; Bodier-Houllé et al. 1998; Rodríguez-Hernández et al. 2005; Tseng et al. 2006). However, data are available that the “central dark line” appears to be the calcium richest part of the human tooth enamel crystallites (Gasga et al. 2008). As described above for bones, X-ray diffraction studies revealed that the biological apatite of younger teeth was less crystalline than that of more mature ones, while the maximum crystallinity was found for teeth belonged to people of 31–40 years old (Pankaew et al. 2012). Therefore, maturation of teeth also means a slow transformation (re-crystallization?) of biological CaPO_4_ from ion-substituted ACP to a better-crystallized ion-substituted CDHA. However, crystallinity of teeth belonged to older people (41–50 and 51–60 years old) was found to decrease with age. Simultaneously, thermogravimetric studies revealed bigger weight loss for teeth of the older people (Pankaew et al. 2012), which could be due to a greater amount of carbonates.

The development of individual enamel and dentine crystals was studied by high-resolution transmission electron microscopy. Both processes appear to be roughly comparable and were described in a four-step process. The first two steps include the initial nucleation and formation of nano-sized particles of biological apatite. They are followed by ribbon-like crystal formation, which until recently was considered as the first step of biological crystal formation (Cuisinier et al. 1993; Houllé et al. 1997). These complicated processes, starting with the heterogeneous nucleation of inorganic CaPO_4_ on an organic extracellular matrix, are controlled in both tissues by the organic matrix and are under cellular control (Bodier-Houllé et al. 1998; Mann 1993). To complicate the process even further, regular and discrete domains of various charges or charge densities on the surface of apatite crystals derived from the maturation stage of enamel development were discovered by a combination of atomic and chemical force microscopy (Kirkham et al. 2000). Binding of organic molecules (e.g., amelogenin (Kirkham et al. 2000)) at physiological solution pH appears to occur on the charged surface domains of apatite. The recent visions on dental tissue research are available elsewhere (Smith and Tafforeau 2008).

As teeth consist of several materials, there are mutual junctions among them. For example, a dentine–enamel junction (DEJ) is a thin [2.0 ± 1.1 μm (Habelitz et al. 2001)] but gradual interface with characteristics transiting from those of dentine to those of enamel. Namely, the collagen fibers in dentine were found to enter into the enamel side of DEJ and terminate in a region in which crystals of biological apatite begin to show enamel characteristics (Chan et al. 2011). In addition, the average contents of organic matrix and carbonate ions were found to increase in the order: enamel < DEJ < dentin. Furthermore, HPO_4_^2−^ ions were not detected in the DEJ, while in dentin their content was higher than in enamel (Kolmas et al. 2010). Comparable results were obtained in another study (Desoutter et al. 2014). Besides, both chemical and molecular structure of DEJ appeared to be dependent on the intratooth location (Xu et al. 2009). Genetically, it is a remnant of the onset of enamel formation because enamel grows outwards from this junction (Nanci 2012). Mechanically, DEJ plays an important role in preventing crack propagation from enamel into dentine (Imbeni et al. 2005). Hierarchically, DEJ has a three-level structure: 25–100 μm scallops with their convexities directed toward the dentin and concavities toward the enamel, 2–5 μm microscallops and a smaller scale structure (Marshall et al. 2003). Therefore, high-resolution elastic modulus mapping has indicated that the DEJ is a band with a graded mechanical property rather than a discrete interface (Sui et al. 2014). The major steps of enamel crystal growth at the junction have been described above but the mechanism of the junction formation is still debatable. Some authors claim that enamel crystals grow epitaxially on the pre-existing dentine crystals because of a high continuity between enamel and dentine crystals (Arsenault and Robinson 1989; Hayashi 1992, 1993). Others have shown that enamel crystals are formed at a given distance from the dentine surface (Simmer and Fincham 1995; Diekwisch et al. 1995; Aoba 1996; Bodier-Houllé et al. 2000) and could either reach dentine crystals by a subsequent growth (Takano et al. 1996) or remain distant (Bodier-Houllé et al. 2000; Dong and Warshawsky 1996). In addition, there are both a cementum–enamel junction (CEJ) (Wang et al. 2006b), which is quite similar to DEJ, and a cementum–dentine junction (CDJ) (Ho et al. 2004, 2005; Jang et al. 2014).

Enamel formation, or amelogenesis, is a highly regulated process involving precise genetic control as well as protein–protein interactions, protein–mineral interactions and interactions involving the cell membrane. Much is still unknown about the interactions among proteins present in enamel matrix and the final crystalline phase of biological apatite (Palmer et al. 2008; Paine et al. 2001). At some point before a tooth erupts into the mouth, the ameloblasts are broken down. Consequently, unlike bones, enamel has no way to regenerate itself using the process of “active mineralization” (see bone formation) because there is no biological process that repairs degraded or damaged enamel (Avery 2001; Nanci 2012). In addition, certain bacteria in the mouth feed on the remains of foods, especially sugars. They produce lactic acid, which dissolves the biological apatite of enamel in a process known as enamel demineralization that takes place below the critical pH of about 5.5. Similar process called enamel erosion occurs when a person consumes acid-containing (citric, lactic, phosphoric, etc.) soft drinks (Jandt 2006; Barbour and Rees 2004; Low and Alhuthali 2008; White et al. 2010). Evidences exist that there is a preferential loss of carbonates and Mg during acidic dissolution of mineral in dental caries. Luckily, saliva gradually neutralizes the acids that cause pH on teeth surface to rise above the critical pH. This might cause partial enamel remineralization, i.e., a return of the dissolved CaPO_4_ to the enamel surface. Until recently, it was generally agreed that if there was sufficient time between the intake of foods (generally, 2–3 h) plus a damage was very limited, teeth could repair themselves by the “passive mineralization” process (LeGeros 1999). Data on increased remineralization of dental enamel by CaPO_4_-containing compounds (Cochrane et al. 2008; Langhorst et al. 2009; Weir et al. 2012) are in support of this hypothesis.

However, studies performed using atomic force microscopy nano-indentation technique revealed that previously demineralized samples of dental enamel further exposed to remineralizing solutions did show a dense crystalline layer of CaPO_4_ formed on their surface. Unfortunately, the re-precipitated deposits of CaPO_4_ always consisted of loosely packed crystals and did not protect the underlying enamel from a subsequent acid attack. Furthermore, these surface deposits were completely removed by either a toothbrush or a short exposure to an erosive acidic solution (Jandt 2006; Lippert et al. 2004a, b, c). In this context, it should be emphasized that the term “remineralization”, which is often misused in the literature, should imply the process of mineral growth that goes hand in hand with a strengthening effect of the weakened enamel surface. Since no strengthening of an exposure to remineralizing solutions was observed, it might be considered that no “passive mineralization” was found (in spite of the real evidence of the re-precipitated surface deposits of CaPO_4_) (Jandt 2006; Lippert et al. 2004b, c).

An interesting hypothesis that nano-sized apatite crystallites occur in the oral cavity during extensive physiological wear of the hierarchical structured enamel surface due to dental abrasion and attrition has been published (Hannig and Hannig 2010). These nano-scaled apatite enamel crystallites might promote remineralization at the tooth surface. However, this idea should be verified experimentally. Thus, according to the current knowledge, the enamel self-repairing ability by a passive remineralization appears to be doubtful, while an active remineralization is impossible. Nevertheless, investigations in this field keep going (Karlinsey and Mackey 2009; Karlinsey et al. 2010a, b; Busch 2004; Onuma et al. 2005; He and Feng 2007; Li et al. 2008b, 2014a; Wang et al. 2009; Roveri et al. 2009a, b; Peters et al. 2010; Orsini et al. 2010; Uysal et al. 2010; Niu et al. 2014). For example, ACP-containing orthodontic biocomposite resins might reduce the enamel decalcification found in patients with poor oral hygiene (Uysal et al. 2010). Further details on CaPO_4_ application in dentistry are available in a topical review (Dorozhkin 2013c).

A content of fluoride added to either toothpaste or mouthwash lowers the solubility of CaPO_4_ (by formation of FHA on the surface) and therefore improves the acid resistance of dental enamel (Schemehorn et al. 2011; Hattab 2013; Driessens 1973; Moreno et al. 1974; McClendon 1966). Furthermore, fluorides also reduce production of acids by bacteria in the mouth by reducing their ability to metabolize sugars. However, dental treatment by fluorides must be used with care because an improper treatment results in formation of CaF_2_ globules deposited on the enamel surface (Wang et al. 2008a).

To conclude the teeth subject, let me briefly mention on the practical application of teeth. Due to relatively small dimensions of normal teeth, only tusks and ivory of giant animals are widely used. For example, both the Greek and Roman civilizations used large quantities of ivory to make high value works of art, precious religious objects and decorative boxes for costly objects. Ivory was often used to form the whites of the eyes of statues. Prior to introduction of plastics, it was used for billiard balls, piano keys, buttons and ornamental items. The examples of modern carved ivory objects are small statuary, netsukes, jewelry, flatware handles and furniture inlays.

### Antlers

**Fig. 16:**
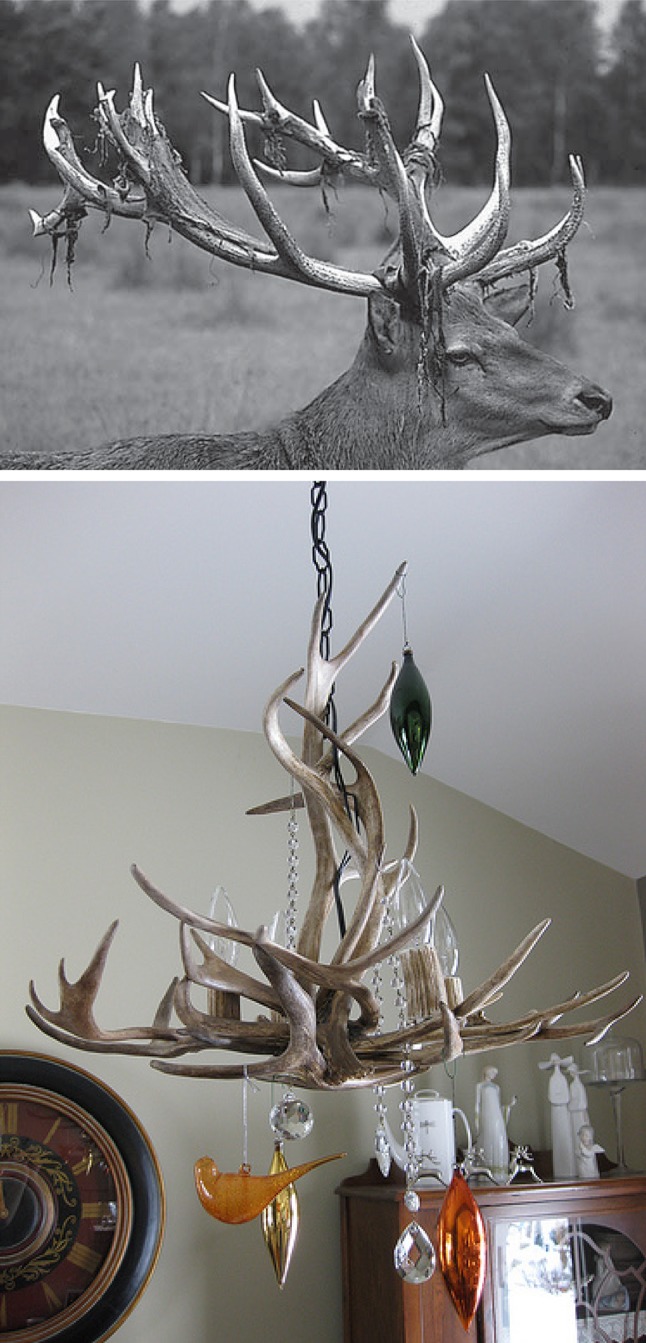
*Top* *red* deer stag at velvet shedding. The bare bone of the hard antlers is exposed. Reprinted from Ref. (Kierdorf and Kierdorf 2011) with permission. A good cross-sectional image of a deer antler is available in Ref. (Meyers et al. 2008). *Bottom* fallen antlers used to make a chandelier

Deer antlers (Fig. 16 top) are unique biological structures since their growth rate is without parallel in vertebrates and because they are the only bony appendages in mammals capable of complete regeneration. This allows for basic research in bone biology without the interference of surgical procedures and their adverse effects in animals where samples are obtained. In addition, antlers also allow for the gathering of a large amount of samples from different populations to assess nutritional and ecological effects on bone composition and structure (Yue et al. 2005; Zhao et al. 2006; Landete-Castilleijos et al. 2007a). They are costly sexual secondary characters of male deer and constitute 1–5 % of the body weight (Huxley 1931). Recent studies suggest that antler regeneration is a stem cell-based process and that these stem cells are located in the pedicle periosteum (Kierdorf et al. 2009; Kierdorf and Kierdorf 2011).

Antlers are not true horns; they are a simple extension of bone. Both antlers and bones utilize the same basic building blocks, namely the type I collagen in the protein phase and the carbonated CDHA in the mineral phase, which was verified by amino acid analysis, X-ray diffraction and TEM observations (Chen et al. 2009). Antlers are large and complex horn-like appendages of deer consisting of bony outgrowths from the head with no covering of keratin as found in true horns. Usually, they begin growing in March and reach maturity in August. In winter, antlers fall off; this is known as shedding. Similar to bones, antlers contain pores and can withstand applied stresses of over 300 MPa (Landete-Castilleijos et al. 2007b; Evans et al. 2005; Akhtar et al. 2008; Currey et al. 2009), which is even higher than that of bones (Table 3). Therefore, antlers are occasionally considered an almost unbreakable bone (Currey et al. 2004). However, there are several distinct differences between them. Namely, antlers and skeletal bones have different functions. The primary functions of antlers are social display, defense against predators and combat between male species. Skeletal bones contain bone marrow, whereas antlers have no marrow. There exists a transition zone between cortical and cancellous bones in antlers, whereas there is no such transition zone in skeletal bones. The cancellous bone is well aligned and uniformly distributed through the entire antler. In bovine femur, the cancellous bone is mainly located in the femur head and its density decreases progressively toward the central region of the femur, which correlates to the external loading conditions. In addition, antlers have a lower mineral content and consequently lowest elastic modulus among mineralized calcified tissues with a mineral content of ~50 wt% (or ~30 vol. %). Interesting that antlers may act as large hearing aids; namely, moose with antlers have far more sensitive hearing than moose without them (Bubenik and Bubenik 2008).

Since antlers are accessible, shed after mating season and cast every year, they appear to be a good model to study bone biology (Price et al. 2005; Landete-Castilleijos et al. 2007c; Li et al. 2014b). Each antler grows from an attachment point on the skull called a pedicle. While an antler is growing, it is covered with highly vascular skin called velvet, which supplies oxygen and nutrients to the growing bone. Antlers bud and branch as they grow. Once they have achieved the proper dimensions, the velvet starts to dry out, cracks and breaks off, while the antler’s bone dies. Therefore, fully developed antlers consist of dead bone only (Kierdorf and Kierdorf 2000, 2005; Pathak et al. 2001; Yuxia et al. 2002; Li et al. 2005). However, the formation and mineralization process of antlers is still not fully understood. To clarify this, researchers used oxytetracycline injections to label different stages of bone formation in antlers of 14 red deer between days 28 and 156 of antler growth. The results revealed that initially a trabecular scaffold of woven bone was formed which largely replaced a pre-existing scaffold of mineralized cartilage. Lamellar bone was then deposited and from about day 70 onwards, primary osteons filled in the longitudinal tubes lined by the scaffold in a proximal to distal sequence (Gomez et al. 2013). In addition, it was found that food processing cannot supply the mineral needs required for antler growth and thus, male deer must temporarily resorb CaPO_4_ minerals from their own skeleton for antler growth (Meister 1956; Muir et al. 1987; Baxter et al. 1999). Detailed studies revealed that daily food intake provided between 25 and 40 % of calcium needed for antler mineralization, which resulted in a temporary skeleton demineralization (Muir et al. 1987; Baxter et al. 1999).

One should note that people seldom come across the antlers in the woods. Rabbits and rodents such as mice and chipmunks eat antlers (and bones of wild animals after they die) for calcium. Rodents and rabbits also gnaw bones and antlers to sharpen their incisors. Due to an extremely high growth rate, which can achieve 2–4 cm per day, combined with a very fast biomineralization, these unique appendages might be a well-suited animal model for studying the disturbances of bone formation induced by additives (e.g., by excess of fluoride) (Kierdorf and Kierdorf 2005). Antler size and external characteristics were found to be influenced by nutrition, climatic variability and other factors. Thus, since antlers are periodically replaced, the analysis of naturally cast antlers offers the opportunity for a continuous and a noninvasive monitoring of the environmental pollution by these additives (Kierdorf and Kierdorf 2005).

To conclude this part, let me briefly mention on the practical application of antlers. Associated with aristocracy, antlers have adorned European castles and hunting lodges for centuries. Today, furnishings and accessories made from antlers are featured in fine homes throughout the world and are a reflection of grace and elegance (Fig. 16 bottom). Concerning biomedical applications, the initial attempts to evaluate a potential use of deer antlers as xenogenic bone grafts have been already performed (Baciut et al. 2007; Hasan et al. 2012; Zhang et al. 2012, 2013).

## Pathological calcification of CaPO_4_

In the body of mammals, osteoblasts and odontoblasts fix ions of calcium and orthophosphate and then precipitate biological apatite onto an organic matrix. This is the process of physiological biomineralization that is restricted to the specific sites in skeletal tissues, including growth plate cartilage, bones, teeth and antlers (Lowenstam and Weiner 1989; Daculsi et al. 1997). Normally, mammals are supposed to die with CaPO_4_ located in bones and teeth (and antlers for male deer) only and nowhere else, because under the normal conditions soft tissues are not mineralized. Unfortunately, owing to aging, various diseases and under certain pathological conditions blood vessels, muscles, extracellular matrix of articular cartilaginous tissues of the joints and some internal organs are calcified as well. This process is called pathological calcification or ectopic (bio)mineralization and leads to a morbidity and a mortality (Lowenstam and Weiner 1989; Daculsi et al. 1997; Block et al. 1998). In general, any type of abnormal accumulation of CaPO_4_ in wrong places is accounted for by a disruption of systemic defense mechanism against calcification (Kazama et al. 2006).

To the best of my findings (Dorozhkin 2012a, 2013a), the earliest paper on a negative influence of unwanted depositions of CaPO_4_ in the body was published as early as in 1798 (Pearson 1798). According to the data available (Poloni and Ward 2014), the unwanted depositions could consist of many substances and all of them always lead to various diseases. Regarding the unwanted depositions of CaPO_4_, they are found in soft tissue calcification (in damaged joints, blood vessels, dysfunctional areas in the brain, diseased organs, scleroderma, prostate stones) (Reid and Andersen 1993; Scotchford and Ali 1995; P’ng et al. 2008; Brancaccio and Cozzolino 2005; Goff and Reichard 2006; Bittmann et al. 2003; Molloy and McCarthy 2006; Giachelli 2004; Kazama et al. 2007), kidney (Giannossi and Summa 2012; Mukherjee 2014) and urinary (Zhu et al. 2014; Huo et al. 2015; Selvaraju et al. 2015) stones, dental pulp stones and dental calculus (Kakei et al. 2009; Kodaka et al. 1988, 1998; Çiftçioglu et al. 1998; Hayashizaki et al. 2008), salivary stones (Zelentsov et al. 2001; Luers et al. 2014), gall stones (Qiao et al. 2013; Hussain and Al-Jashamy 2013), pineal gland calcifications (Güney et al. 2013), atherosclerotic arteries and veins (Ortlepp et al. 2004; Tomazic 2001; Marra et al. 2006; Kurabayashi 2013), coronary calcification (Fitzpatrick et al. 2003; Matsui et al. 2015), damaged cardiac valves (Suvorova and Buffat 2005), calcification on artificial heart valves (Giachelli 2001; Pettenazzo et al. 2001; Schoen and Levy 2005; Delogne et al. 2007), carpal tunnel (Sensui et al. 2003; Namba et al. 2008) and tumoral (Namba et al. 2008; Carlson et al. 2007; Sprecher 2010; Slavin et al. 2012; Kim et al. 2013; Burns et al. 2013) calcifications, cataracts (Kim and Choi 2007; Koinzer et al. 2009), malacoplakia (Ho 1989), calcified menisci (Katsamenis et al. 2012; Dessombz et al. 2013), dermatomyositis (Stock et al. 2004; Pachman and Boskey 2006) and still other places (Daculsi et al. 1997; Bazin et al. 2014b). In addition, there is a metastatic calcification of non-osseous viable tissue occurring throughout the body (Hale 2003; Alkan et al. 2009), but it primarily affects the interstitial tissue of the blood vessels, kidney, lungs and gastric mucosa. A metastatic calcification is defined as a deposition of CaPO_4_ in previously normal tissue due to an abnormal biochemistry with disturbances in the calcium or phosphorus metabolism (Grech et al. 1998). Common causes of the metastatic calcification include hyperparathyroidism, chronic renal disease, massive bone destruction in widespread bone metastases and increased intestinal calcium absorption. One author has mentioned on “apatite diseases” which are characterized by the appearance of needle-like crystals comparable to those of bone apatite in the fibrous connective tissue (Mohr 2003). All these cases are examples of a calcinosis (Sprecher 2010; Slavin et al. 2012; Kim et al. 2013; Burns et al. 2013), which might be described as a formation of undesired CaPO_4_ deposits in any soft tissue. In dentistry, a calculus or a tartar refers to a hardened plaque on the teeth, formed by the presence of saliva, debris and minerals (White 1997). Its rough surface provides the ideal medium for bacterial growth, threatening the health of the gums and absorbing unaesthetic stains far more easily than natural teeth (LeGeros 1991).

Calcifying nanodimensional particles are the first CaPO_4_-containing particles isolated from human blood and were detected in numerous pathologic calcification related diseases (Ciftçioğlu and McKay 2010). Interestingly, contrary to the mineral phases of normal calcifications (bone, dentine, enamel, cementum, antlers), which consist of only one type of CaPO_4_ (namely, biological apatite), the mineral phases of abnormal and/or pathological calcifications are found to occur as single or mixed phases of other types of CaPO_4_ (ACP, DCPD, OCP, β-(Ca,Mg)_3_(PO_4_)_2_) and/or other phosphate and non-phosphate compounds (e.g., magnesium orthophosphates, calcium pyrophosphates, calcium oxalates, etc.) in addition to or in place of biological apatite (Table 5; LeGeros 1991, 2001; Amjad 1997; Daculsi et al. 1997; Qiu and Orme 2008; Kodaka et al. 1988; Reid and Andersen 1993; Scotchford and Ali 1995; P’ng et al. 2008; Bazin et al. 2014b; Lee et al. 2006; Rosenthal 2007; Lagier and Baud 2003; Wesson and Ward 2007). However, precipitation of biological apatite in wrong places is also possible; this is the so-called “HA deposition disease” (Burns et al. 2013; Hayes and Conway 1990; Best et al. 1997; Garcia et al. 2003; Melrose et al. 2009).

**Table 5:** Occurrence of calcium phosphates in biological systems (human) (LeGeros 2001)

Calcium phosphate	Occurrence
Biological apatite	Enamel, dentine, bone, dental calculi, stones, urinary stones, soft-tissue deposits
OCP	Dental calculi and urinary stones
DCPD	Dental calculi, crystalluria, chondrocalcinosis, in some carious lesions
β-(Ca,Mg)_3_(PO_4_)_2_	Dental calculi, salivary stones, arthritic cartilage, soft-tissue deposits
Ca_2_P_2_O_7_·2H_2_O	Pseudo-gout deposits in synovium fluids
ACP	Heart calcifications in uremic patients, kidney stones

Occurrence of non-apatite phases in the pathological calcifications may indicate that they were crystallized under the conditions different from homeostasis or crystallization of the apatite structures was inhibited and less stable phases crystallized instead, without further change to the more stable one. Furthermore, in the places of pathological calcifications the solution pH is often relatively low. Given that nucleation and crystal growth is not a highly regulated process in any pathological deposits, there is not likely just one fundamental formation mechanism for all possible calcification types. Furthermore, various bioorganic impurities in the local environment undoubtedly influence the crystallization process, resulting in a great variety of pathological deposits. Thus, it is a highly complex problem. In some cases, the chemical composition of an unwanted inorganic phase might depend on the age of the pathological calcification and its location. For example, DCPD is more frequently found in young (3 months or younger) calculus, biological apatite is present in all ages of calculus, while β-(Ca,Mg)_3_(PO_4_)_2_ occurs more frequently in sub-gingival calculus. In mature calculus, the relative abundance of OCP, β-(Ca,Mg)_3_(PO_4_)_2_ and biological apatite also differ between the inner and outer layers (LeGeros 2001). It is interesting to note that the mineral phases of animal calculus (e.g., from dog) was found to consist of calcium carbonate and biological apatite, while human calculi do not contain calcium carbonate (LeGeros 2001; LeGeros et al. 1988).

The nucleation process is the main step in both normal and pathological calcifications. In vitro experiments performed to simulate the formation of sedimentary urinary stones demonstrated that in the absence of organic matter no CaPO_4_ crystallized in cavities with scarce liquid renovation, but regular CDHA layers appeared on the wall around the cavity (Grases and Llobera 1998). Visible deposits of calcified organic materials (mixtures of organic matter and spherulites of CDHA) were formed when a glycoprotein (mucin) was present. In this case, the walls of the cavity as well as the glycoproteins had the capacity to act as heterogeneous nucleators of CaPO_4_. CDHA microcrystal nucleation on the surface of epithelial cells can be a critical step in the formation of kidney stones (Lieske et al. 1997) and identical mechanisms can be thought for unwanted calcifications in other soft tissues of the body, such as cardiac valves or vascular ducts. Monolayers of CDHA crystals can bind to epithelial cells. A large amount of kidney stones contains CDHA as the crystallization nuclei.

In general, formation of crystals in pathological mineralizations follows the same principles as normal calcifications (Kirsch 2006, 2007). Namely, local conditions for nucleation require a certain degree of local supersaturation induced by biochemical processes, which can be promoted by deficiency of inhibitors (like diphosphate, Mg^2+^ or even citrate ions) and/or the presence of matrix of a bioorganic material (such as cholesterol) or other crystals of different solids, those might act as heterogeneous nuclei. In addition, other regulators (activators and inhibitors) of physiological biomineralization have been identified and characterized (Kirsch 2006, 2007; Speer and Giachelli 2004; Giachelli 2005; Giachelli et al. 2005; Schmitt et al. 2006; Azari et al. 2008). What is more, the biological fluids (e.g., serum, saliva, synovial fluids) are normally supersaturated with respect to biological apatite precipitation (LeGeros 1991, 2001; Lowenstam and Weiner 1989); therefore, in principle, calcification is thermodynamically feasible in any part of the body. However, normally it is not the case. Therefore, in the healthy body, the appropriate inhibitory mechanisms must be at work to prevent a superfluous calcification of soft tissues. These inhibition mechanisms are a hot research topic in molecular medicine but this subject is beyond the scope of current review.

**Fig. 17:**
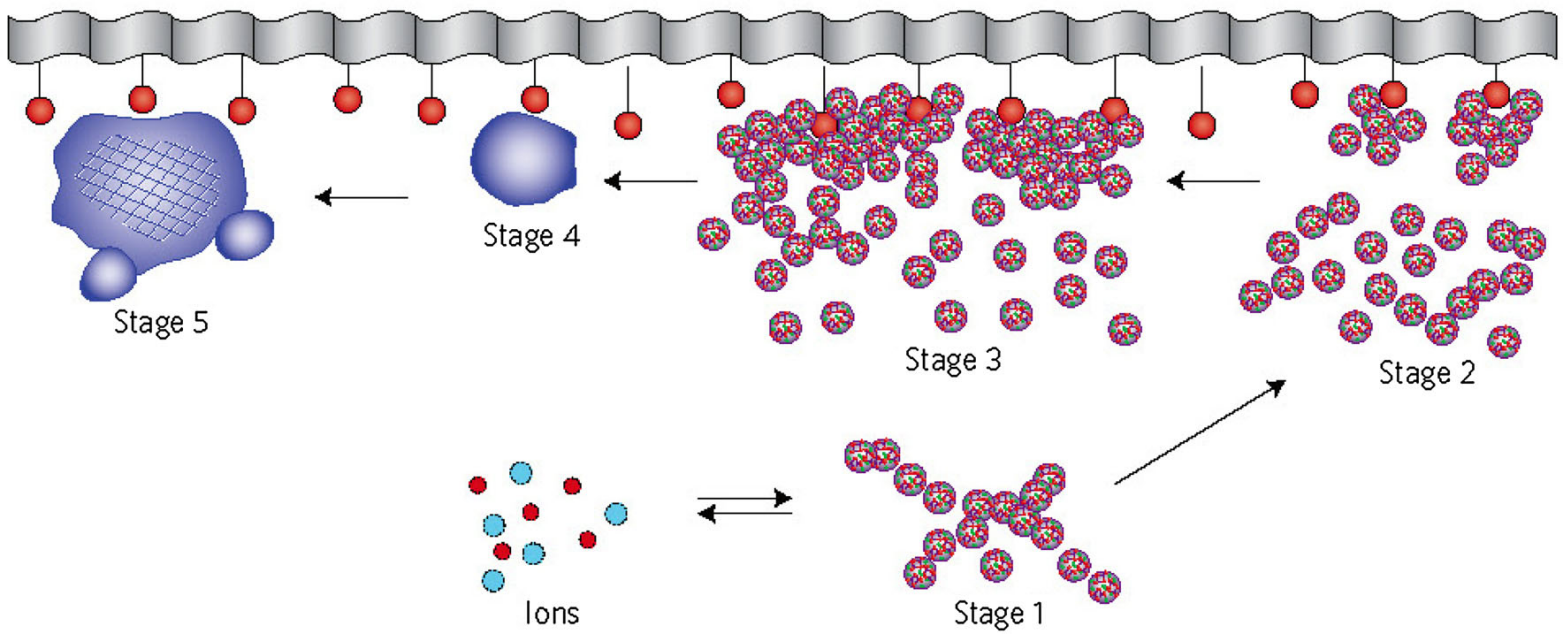
A schematic representation of the different stages of a surface-directed mineralization of CaPO_4_. In stage 1, aggregates of pre-nucleation clusters are in equilibrium with ions in solution. The clusters approach a surface with chemical functionality. In stage 2, pre-nucleation clusters aggregate near the surface, with loose aggregates still in solution. In stage 3, further aggregation causes densification near the surface. In stage 4, nucleation of spherical particles of ACP occurs at the surface only. In stage 5, crystallization occurs in the region of the ACP particles directed by the surface. Reprinted from Refs. (Cölfen 2010; Dey et al. 2010) with permission

In 2010, an arachidic acid Langmuir monolayer system was reported as a model for pathological mineralization of ion-substituted carbonate apatites from simulated body fluid (Dey et al. 2010). The authors demonstrated that the surface-induced formation of carbonateapatite started from aggregation of pre-nucleation clusters of yet unknown CaPO_4_ leading to nucleation of ACP before further development of oriented apatite crystals. This process is schematically shown in Fig. 17 (Cölfen 2010; Dey et al. 2010).

To conclude this part, it is worth reminding that CaPO_4_ of the biological origin are sparingly soluble in aqueous solutions. Therefore, removing them from the places of unwanted deposition would be an equivalent of bone demineralization; that is a challenge. Thus, the majority of therapeutic approaches are directed at preventing the progression of pathological calcifications. Among them, a chelation therapy might be of some interest to chemists and materials researchers because it deals with chemical processes (Lamas and Ackermann 2000; Knudtson et al. 2002). The general principles of demineralization and decalcification (i.e., removing the mineral Ca-containing compounds (phosphates and carbonates) from the bioorganic matrix) have been extensively reviewed (Ehrlich et al. 2008, 2009), where the interested readers are referred to.

## Biomimetic crystallization of CaPO_4_

The term “biomimetics” (“the mimicry of life”) was coined by an American inventor, engineer and biophysicist Otto Herbert Schmitt (1913–1998) in the 1950s. Biomimetics (also known as bionics, biognosis and/or biomimicry) might be defined as application of the methods and systems found in nature to the study, design and construction of new engineering systems, materials, chemical compounds and modern technology. Another definition describes biomimetics as a micro-structural process that mimics or inspires the biological mechanism, in part or as a whole (Green et al. 2002). This biological process generates highly ordered materials with a hybrid composition, a complex texture and ultra-fine crystallites through a hierarchical self-assembly and begins by designing and synthesizing molecules that have an ability to self-assemble or self-organize spontaneously to higher order structures.

Biomimetism of synthetic materials for biomedical applications can be carried out at different levels in view of composition, structure, morphology, bulk and surface chemical-physical properties. Chemists, biologists, physicists and engineers interested in material science are amazed by the high degree of sophistication, miniaturization, hierarchical organization, hybridizing, reliability, efficiency, resistance and adaptability characterizing the natural materials. These properties, which biogenic materials have achieved through specific building principles selected by evolution, can be only partially obtained in manmade materials by present synthetic processes. For this reason, Nature is a school for material science. Biomimetism and bioinspiration represent important tools for the design and the synthesis of innovative materials and devices (Roveri et al. 2009b).

Historically, the biomimetic concept is very old (e.g., the Chinese wanted to make artificial silk ~3000 years ago; Daedalus’ wings was one of the early design failure), but the implementation is gathering momentum only in the 20th century. The first papers with the term “biomimetics” in the title were published in 1972 (Burke et al. 1972; Breslow 1972). In spite of the tremendous achievements of modern science and technology, the nature’s ability to assemble inorganic compounds into hard tissues (shells, spicules, teeth, bones, antlers, skeletons, etc.) is still not achievable by the synthetic procedures. This is not surprising—designs found in nature are the result of millions of years of evolution and competition for survival. The models that failed are fossils; those that survived are the success (Benyus 1997). In the frames of this review, biomimetics is considered as mimicking natural manufacturing methods to generate artificial calcified tissues (grafts, implants, prostheses) those might be used as temporary or permanent replacements of the missing, lost, injured or damaged bones and teeth. It is important to notice that precipitation of CaPO_4_ and calcium carbonates has been considered to correlate with bone formation, at least, since 1923 (Watt 1923).

A key step in the biomimetic bone graft production is attributed to the crystal growth of apatite phase onto a collagen matrix. Therefore, the matter of choosing the correct experimental conditions and well-mimicking solutions is of the primary importance. The easiest way to perform the crystallization would be mixing of aqueous solutions containing the ions of calcium and orthophosphate (LeGeros 1991; Elliott 1994; Amjad 1997). Unfortunately, such type of crystallization provides precipitates with the properties (chemical composition, Ca/P ratio, crystallinity level, particle size distribution, etc.) far different from those of biological apatite. This can be explained by the following paramount differences between the in vivo biological and in vitro chemical crystallization conditions (Dorozhkin et al. 2004):

1. In vitro crystallization normally occurs at permanently depleting concentrations of calcium and orthophosphate ions, while the concentrations of all ions and molecules are kept strictly constant during biological mineralization (the same is valid for the solution pH);
2. Chemical crystallization is a fast process (time scale of minutes to days), while the biological process is a slow one (time scale of weeks to years);
3. Many inorganic, bioorganic, biological and polymeric compounds are present in biological liquids (blood plasma, serum, saliva). Each of these compounds might act as an inhibitor, promoter, nucleator or even as a template for the growth of biological apatite. In addition, each of them somehow influences the crystallization kinetics and might be either incorporated into the solid structure or co-precipitated with CaPO_4_ (Jahromi et al. 2013).
4. Chemical crystallization is, by all means, a “passive” process, while the biological mineralization is strongly influenced by cells and occurs by the self-organization mechanisms (Boskey and Roy 2008; Sato 2006; Hartgerink et al. 2001). Still there are no good ways to overcome this difference.

The first and the second differences might be overcome using the appropriate crystallization techniques. The details are available elsewhere (Dorozhkin et al. 2004) but, briefly, the first problem might be overcome by either a continuous flow of a supersaturated solution (Izquierdo-Barba et al. 2000; Vallet-Regí et al. 2000) or using a constant-composition (CC) technique (Nancollas and Wu 2000; Koutsoukos et al. 1980; Tomson and Nancollas 1978). The second difference might be surpassed by a restrained diffusion of calcium and orthophosphate ions from the opposite directions in, for example, a double-diffusion (DD) crystallization device or in viscous gels (Busch et al. 1999; Manjubala et al. 2006; Cai et al. 2010; Yokoi et al. 2010; Sadjadi et al. 2010). The CC and DD techniques have been combined into a single constant-composition double-diffusion (CCDD) device, which currently seems to be the most advanced experimental tool to perform biomimetic crystallization (Dorozhkin et al. 2003, 2004; Dorozhkin and Dorozhkina 2003, 2005; Dorozhkina and Dorozhkin 2003a; Dorozhkin 2007b). However, in no case, the CCDD device should be considered as the final construction; it still has much room for further improvement, e.g., by upgrading the design of the crystallization chamber (Becker and Epple 2005). Other constructions, e.g., to study calcification of biological heart valve prostheses (Krings et al. 2006), are also possible. In addition, one should keep in mind that the potential of the standard CC technique has not reached its limit yet: for example, a good mimicking of the self-organized microstructure of tooth enamel has been achieved (Wang et al. 2008b).

The third major difference between the in vivo and in vitro crystallization conditions might be overcome using the appropriate crystallization solutions (Dorozhkin et al. 2004). To the best of my findings (Dorozhkin 2012a, 2013a), the presence of calcium and orthophosphate ions in the biological fluids (blood, saliva, lymph, etc.) has been known since, at least, 1804 (Fourcroy 1804). Therefore, the best way would be to perform experiments using these natural liquids, but this is not easy due to both a great variability of their chemical and biochemical compositions and problems with their collection and storage. As stated before, using supersaturated aqueous solutions containing only the ions of calcium and orthophosphate appears to be unable to mimic the crystallization of biological apatite; therefore, more advanced solutions have been elaborated. According to the topical review on the subject (Tas 2014), a formulation developed by Tyrode in 1910 (Tyrode 1910) was the first simulating medium, containing dissolved ions of calcium and orthophosphate together with other inorganic ions; however, no information was found on applicability of that solution for CaPO_4_ crystallization. Therefore, the earliest solution, suitable for CaPO_4_ crystallization was introduced 1943 by Earle (Earle 1943) and called Earle’s balanced salt solution (EBSS). EBSS contains inorganic salts (NaCl, KCl, CaCl_2_, MgSO_4_, NaHCO_3_, NaH_2_PO_4_) and was successfully used for CaPO_4_ crystallization (Termine and Eanes 1974). Other popular physiological solutions comprise Hanks’ balanced salt solution (HBSS), which contains almost similar inorganic salts and glucose (Hanks and Wallace 1949; Shibata et al. 2004; Marques et al. 2003a; Mareci et al. 2010), Eagle’s minimum essential medium (MEM) and its variation Dulbecco’s modified Eagle’s medium (DMEM), which contain numerous bioorganic (alanine, aspartic acid, glycine, biotin, vitamin C, folic acid, riboflavin) and inorganic (CaCl_2_, KCl, NaCl, NaH_2_PO_4_) components (Meuleman et al. 2006; Touny et al. 2011; Coelho et al. 2000; Mandel and Tas 2010; Rohanová et al. 2014), phosphate-buffered saline (PBS) that contains only inorganic (CaCl_2_, MgCl_2_, KCl, KH_2_PO_4_, NaCl, NaH_2_PO_4_) components (Gao et al. 2006; Lichtenauer et al. 2011). Furthermore, artificial saliva (Sato et al. 2006; Ionta et al. 2014; Okulus et al. 2014), synthetic urine (Assimos 2013; Dbira et al. 2015) and simulated milk ultrafiltrate (SMUF) (Jenness and Koops 1962; Spanos et al. 2007; Gao et al. 2010a, b) solutions are available. They contain both bioorganic (e.g., xanthan gum or sodium carboxymethylcellulose, sorbitol, etc.) and inorganic (e.g., CaCl_2_, MgCl_2_, KCl, KH_2_PO_4_, NaCl, KH_2_PO_4_) compounds. Additional information on the media and physiological solutions used for mineralization studies is available elsewhere (Boskey and Roy 2008; Tas 2014). The majority of the aforementioned simulating solutions are commercially available.

However, the most popular biomimetic solution is a protein-free acellular simulated body fluid (SBF). It was introduced in 1990 by Kokubo et al., (Kokubo et al. 1990) and occasionally named as Kokubo’s SBF. It is a metastable aqueous solution with pH ~ 7.40, supersaturated with respect to the precipitation of OCP, β-TCP, CDHA and HA (Lu and Leng 2005), containing only inorganic ions in concentrations nearly equal to those in human blood plasma. However, the standard SBF formulation, first, contains the tris/HCl buffer, and, second, the concentration of hydrogencarbonate (4.2 mM) is only a fraction of that in blood plasma (27 mM) (Kokubo et al. 1990). The problem of a low concentration of hydrogencarbonate ions has been overcome by first introducing a “synthetic body fluid” (Tas 2000; Landi et al. 2005; Jalota et al. 2008) and later a revised SBF (rSBF) (Kim et al. 2001; Oyane et al. 2003). Due to the chemical similarity with human blood plasma, rSBF currently seems to be the best simulating solution. However, it contains Hepes buffer, loses CO_2_ in open vessels and does not contain any organic and/or biological molecules (Kim et al. 2001; Oyane et al. 2003). Other types of SBF are also available (Müller and Müller 2006; Hu et al. 2006; Wen et al. 2009; Gemelli et al. 2010) and the interested readers are referred to a leading opinion co-authored by the SBF inventor (Kokubo and Takadama 2006), where the entire history and the preparation techniques of various SBF formulations are well described. Later, another leading opinion on the suitability of SBF for the in vitro bioactivity tests was published (Bohner and Lemaitre 2009). The authors demonstrated that (1) there is presently no enough scientific data to support the SBF suitability and (2) even though bioactivity tests with SBFs are valid, the way the tests are generally conducted leaves room for further improvements. In addition, the preparation protocol of SBF solutions was reconsidered and a new procedure was suggested to improve the reproducibility of bioactivity tests (Bohner and Lemaitre 2009). However, the situation with SBF appears to be not so straightforward. Namely, further studies revealed that the actual behavior of osteoblasts was likely to provide the primary measures of biocompatibility and bioactivity, rather than oversimplified and essentially irrelevant tests with SBF (Pan et al. 2010), while the in vitro bioactivity experiments performed with SBF did not always predict the relative in vivo performance of the same implants (Zadpoor 2014).

The application of SBF for the surface mineralization of various materials in vitro has been reviewed (Kim 2003), while the theoretical analysis of CaPO_4_ precipitation (the driving force and the nucleation rate based on the classical crystallization theory) in SBF is also available (Lu and Leng 2005). It is important to note that nanometer-sized pre-nucleation clusters in SBF solutions have been discovered (Dey et al. 2010); those clusters are believed to be the initial building blocks of crystallized CaPO_4_ [e.g., CDHA (Onuma and Ito 1998)], while the crystallization process itself occurs via intermediate formation of ACP (Fig. 17).

Further attempts to improve the biomimetic properties of SBF and rSBF have been performed (Kokubo and Takadama 2006; Bohner and Lemaitre 2009). Efforts were made to replace artificial buffers (tris/HCl, Hepes) with simultaneously increasing the concentration of hydrogencarbonates for SBF (Marques et al. 2003b, 2004; Dorozhkina and Dorozhkin 2002b) or avoiding losses of CO_2_ from open vessels for rSBF (Dorozhkin et al. 2003, 2004; Dorozhkin and Dorozhkina 2003, 2005; Dorozhkina and Dorozhkin 2003a; Dorozhkin 2007b) by means of permanent bubbling of gaseous CO_2_ through the solutions. Addition of the most important organic and biological compounds like glucose (Dorozhkin et al. 2003), albumin (Dorozhkin and Dorozhkina 2003; Marques et al. 2004), lactates (Pasinli et al. 2010) and collagen (Sun and Wang 2010) is another direction to improve biomimetic properties of various types of SBF. Once a cow milk-based rSBF has been prepared (Dorozhkin and Dorozhkina 2007). Further improvements of all biomimetic solutions are to be made in future. Occasionally, condensed solutions of SBF [e.g., 1.5-fold, twofold (Sun and Wang 2010; Miyaji et al. 1999; Kim et al. 2000b), fivefold (Barrere et al. 2002a, b) and even tenfold (Tas and Bhaduri 2004; Demirtaş et al. 2015)] are used to accelerate precipitation and increase the amount of precipitates. However, whenever possible this should be avoided because the application of condensed solutions of SBF leads to changes in the chemical composition of the precipitates; namely, the concentration of carbonates increases, while the concentration of orthophosphates decreases (Dorozhkina and Dorozhkin 2003b).

To conclude this part, one should note on the difficulties in mimicking the calcification process that occurs in bones and teeth. A reasonable mechanism of the induction of CDHA nucleation and crystallization by carboxylate groups on the bioorganic matrices looks as this. At first, calcium and orthophosphate ions are combined with carboxylate groups. By using this as seeds, CDHA crystals then grow to generate interfaces that contain the most stable structure of the {100} faces. Such a crystallization mechanism explains why the *c*-axes of biological apatite are parallel to the organic matrices. Collagen fibers can be regarded as axis-like organic matrices: when CDHA is formed on the surface of collagen fibers parallel to the *c*-axes, the *c*-axes are oriented parallel to the fiber orientation (Sato 2007). A step further would be to perform the precipitation from the simulating solutions on templates of biomineralization proteins for the control of crystal organization and properties. For example, there are successful attempts to crystallize CaPO_4_ on collagen to obtain bone-like composites (Nassif et al. 2010; Li et al. 2011; Xia et al. 2012, 2013, 2014; Antebi et al. 2013). Such collagen/CaPO_4_ biocomposites are currently under investigation for clinical use. Other popular matrixes to perform biomimetic CaPO_4_ crystallization comprise gelatin (Busch et al. 1999; Rosseeva et al. 2008; Liu et al. 2009; Raz et al. 2014), chitosan (Raz et al. 2014; Wang et al. 2011; Lu et al. 2014b), the surface of metals and alloys (Wang et al. 2004; Bigi et al. 2005; Liu et al. 2010, 2013; Dorozhkin 2014), polymers (Iwatsubo et al. 2006), cellulose (Bodin et al. 2006), self-assembled monolayers (Toworfe et al. 2006) and many other materials. Such biomimetically prepared CaPO_4_ precipitates are occasionally called “organoapatites” (Spoerke and Stupp 2003; Storrie and Stupp 2005).

## Conclusions and outlook

By the end of the XX-th century, it became clear that CaPO_4_-based biomaterials and bioceramics by themselves could not give a complete response to the clinical needs for artificial implants. Biomaterials with more demanding properties were required. Namely, in 1998, Prof. Larry L. Hench published a forecast for the future of biomaterials development (Hench 1998), where he noted that available that time bioactive materials (CaPO_4_, bioactive glasses and glass ceramics) had already improved prostheses lifetime but, unfortunately, any type of prosthesis had mechanical limitations. As the solution, he proposed that biomaterial researchers would need to focus on tissue regeneration instead of tissue replacement. A working hypothesis was announced: “Long-term survivability of prosthesis will be increased by the use of biomaterials that enhance the regeneration of natural tissues” (Hench 1998). One path to follow is the regeneration of bone using CaPO_4_-based scaffolds that mimic the structure of biological apatite, bond to bone and in some cases activate the genes within bone cells to stimulate new bone growth (Jones and Hench 2003; Griffith and Naughton 2002; Hench and Polak 2002). Thus, in 2002, Hench predicted a rapid development of tissue engineering field, where CaPO_4_ play an auxiliary role. The practice reveals that tissue engineering, indeed, is a very rapidly developed field of science and research (Ratner and Bryant 2004).

However, what can be said about CaPO_4_ themselves? The major questions on chemistry, crystallization, ion-substitution, crystallography, thermodynamics and phase relationships for the chemically pure CaPO_4_ have been answered in the XXth century. Some important topics for DCPD and CDHA have been additionally investigated in the field of self-setting CaPO_4_ formulations (Dorozhkin 2013b). Conversely, CaPO_4_ of biological origin, including the control of their morphology and interaction of CaPO_4_-based bioceramics with various bioorganic compounds are not well investigated yet. The same is valid for the nanocrystalline and amorphous samples of CaPO_4_. Small amounts of bone-like apatite might be easily prepared by crystallization from simulating solutions, such as SBF and rSBF, but what can be said about larger quantities? A standard way of the concentration increasing causes chemical changes in the precipitates (Dorozhkina and Dorozhkin 2003b). After a necessary technology is developed, one will have to think on scaffold preparation from this material, keeping in mind that any thermal treatment would destroy this material. A spark plasma sintering approach based on the use of pulsed current and enabling very fast heating and cooling rates seemed to be a first hint to achieve this goal (Zhang et al. 2008; Grossin et al. 2010). However, a rapid development of the self-setting CaPO_4_ formulations, which can be easily doped by the necessary chemical elements, seems to be a better solution of this problem (Dorozhkin 2013b). Furthermore, the existence of OA remains to be questionable, as well as the bioactivity mechanism of CaPO_4_ requires better understanding.

To date, although CaPO_4_-based biomaterials and bioceramics have been extensively studied for over 50 years, their ability to trigger bone formation is still incomparable with other biomaterials. Nowadays, the biomaterials’ field is shifting towards biologically active systems to improve their performance and to expand their use (Kolk et al. 2012; Garg and Goyal 2014; Tang and Diehl 2014). Therefore, tissue engineering is the strongest direction of current research, which, in the case of CaPO_4_, means fabrication of proper substrates and/or scaffolds to carry cells, hormones and biochemical factors to be further used in surgery and medicine (Zhou and Lee 2011; Zakaria et al. 2013; Shao et al. 2015). Presumably, a synthesis of various types of CaPO_4_-based biocomposites and hybrid biomaterials occupies the second important place (Dorozhkin 2015b). The third important place is occupied by investigations devoted to the synthesis and characterization of various nano-sized particles and nanodimensional crystals of CaPO_4_ (Dorozhkin 2013d), ACP (Dorozhkin 2012c), as well as CaPO_4_ with controlled particle geometry and shapes (Lin et al. 2014; Galea et al. 2013; Kalia et al. 2014). In general, the geometry of crystal phases can be varied by controlling the precipitation conditions, such as temperature, solution pH, concentration of the reagents, hydrodynamics, presence of various admixtures, inhibitors or promoters and ultrasonication. All these approaches might be useful in preparation of CaPO_4_ fibers, whiskers, hollow microspheres, etc. In addition, a great attention is paid to manufacturing of the self-setting CaPO_4_ formulations (Dorozhkin 2013b) and multiphase formulations (Dorozhkin 2012d) mimicking as closely as possible the mineral component of biological apatite. A work along the ecological ways of synthesis of CaPO_4_ might be of a great importance as well (Dorozhkin 2008). A deeper study of the fascinating growth rate of deer antlers and the ability of some animals, such as newts, to regenerate amputated limbs might provide new and unexpected approaches to the bone-healing concept, as well as this will be important for further development of both biomimetics and biomineralization fields. Unfortunately, no currently available grafting biomaterials can substitute the bones’ mechanical function, illustrating yet unmet medical need that would entirely substitute and regenerate a damaged tissue or organ. In a close future, the foreseeable application of CaPO_4_ will be as an integrated component of the third generation of biomaterials (Hench 1998; Hench and Polak 2002), where they will support cells and/or other biologically active substances (peptides, growth factors, hormones, drugs, etc.) to guide regeneration of hard tissues (Daculsi et al. 2013; Feng et al. 2013b; Miura et al. 2013; Wang et al. 2014c).

To finalize this review, one should note that in spite of almost the 250-year long history of the CaPO_4_ research (Dorozhkin 2012a, 2013a) and many important discoveries, still many gaps remain in our knowledge to be investigated in future.
